# Normalization and Selecting Non-Differentially Expressed Genes Improve Machine Learning Modelling of Cross-Platform Transcriptomic Data

**DOI:** 10.53941/tai.2025.100005

**Published:** 2025-05-25

**Authors:** Fei Deng, Catherine H. Feng, Nan Gao, Lanjing Zhang

**Affiliations:** 1 Department of Chemical Biology, Ernest Mario School of Pharmacy, Rutgers University, Piscataway, NJ 08854, USA; 2 Department of Molecular and Cellular Biology, Harvard University, Cambridge, MA 02138, USA; 3 Department of Biological Sciences, School of Arts & Sciences, Rutgers University, Newark, NJ 08854, USA; 4 Department of Pharmacology, Physiology, and Neuroscience, New Jersey Medical School, Rutgers University, Newark, NJ 07103, USA; 5 Department of Pathology, Princeton Medical Center, Plainsboro, NJ 08536, USA; 6 Rutgers Cancer Institute of New Jersey, New Brunswick, NJ 08901, USA

**Keywords:** machine learning, feature selection, normalization, transcriptomics, breast cancer

## Abstract

Normalization is a critical step in quantitative analyses of biological processes. Recent works show that cross-platform integration and normalization enable machine learning (ML) training on RNA microarray and RNA-seq data, but no independent datasets were used in their studies. Therefore, it is unclear how to improve ML modelling performance on independent RNA array and RNA-seq based datasets. Inspired by the house-keeping genes that are commonly used in experimental biology, this study tests the hypothesis that non-differentially expressed genes (NDEG) may improve normalization of transcriptomic data and subsequently cross-platform modelling performance of ML models. Microarray and RNA-seq datasets of the TCGA breast cancer were used as independent training and test datasets, respectively, to classify the molecular subtypes of breast cancer. NDEG (*p* > 0.85) and differentially expressed genes (DEG) (*p* < 0.05) were selected based on the *p* values of ANOVA analysis and used for subsequent data normalization and classification, respectively. Models trained based on data from one platform were used for testing on the other platform. Our data show that NDEG and DEG gene selection could effectively improve the model classification performance. Normalization methods based on parametric statistical analysis were inferior to those based on nonparametric statistics. In this study, the LOG_QN and LOG_QNZ normalization methods combined with the neural network classification model seem to achieve better performance. Therefore, NDEG-based normalization appears useful for cross-platform testing on completely independent datasets. However, more studies are required to examine whether NDEG-based normalization can improve ML classification performance in other datasets and other omic data types.

## 1. Introduction

Normalization is a critical step in quantitative analyses of biological processes, but very difficult yet important in cross-platform comparison [1–3]. Independent dataset is required for rigorous testing of any quantitative biomedical analyses [4,5], while high-throughput transcriptomic data can be obtained using two different platforms, namely RNA microarray and recently RNA-sequencing (RNA-seq) [6–8]. The cross-platform difference makes direct cross-platform testing in an independent dataset challenging, if not impossible. Therefore, this study aimed to improve performance of machine learning (ML) modelling of transcriptomic data in the two commonly used high-through RNA quantification platforms.

Advances in genome sequencing technology have given researchers a whole new perspective on fighting a variety of complex diseases [9–11]. Cancer is a complex genetic disease involving multiple subtypes. In order to better understand this disease to improve the accuracy and reliability of diagnosis, treatment and prediction prognosis, researchers have collected massive amounts of gene expression data in different biological environments and through different assays. Analyzing these data and mining the important relationship between them and the disease also puts brand new requirements on algorithms for data processing, prediction and classification. To rationally and adequately apply these data from different platforms, many researchers consider various ways to eliminate or reduce the data differences cross platforms, and then incorporate them into the same framework for analysis [12–20]. It can directly expand the pool of omic data that can be directly compared. However, it also introduces selection biases by selecting and/or removing features/factors. Therefore, we seek to unbiasedly normalize biological data, while the process may be more complex but more rigorous.

ML methods excel at solving complex problems such as tumor subtype classification, and often are trained using large amounts of data to find the hidden patterns needed to make decisions [12,21–25]. However, there are several key issues when classifying tumor subtypes based on gene expression data, such as high dimensionality and class imbalance [26–28]. High dimensionality of the data refers to the presence of an exceptionally large number of features (e.g., genes in transcriptomic data), compared to that of samples. To address the high dimensionality problem, many feature selection methods and techniques have been devised to remove irrelevant features, reduce model training time, and develop generalized and scalable models [27,29–36]. These feature selection algorithms rely on optimization algorithms or statistical methods and are broadly classified into packing, hybrid and filtering methods. However, there is no generalized method that can handle omic datasets for all platforms. Moreover, gene screening strategies play an important role in finding key genes such as housekeeping gene [37,38]. Most studies have used software, such as GeNorm, BestKeeper and NormFinder, to analyze the expression stability of certain genes of interest in disease groups and healthy controls to identify reference genes. There have also been successes in identifying key genes through ML methods [22,37–46].

Normalization before ML modelling is another issue of successful cross-platform (external) validation and thus warrants extensive studies [4,5,47]. It can effectively address the data biases attributable to platform difference by reducing data variances associated with platform difference, yet retaining the meaningful biological differences. Indeed, the importance of data normalization for constructing predictive models has been demonstrated before [1–3,12,16,23,48–62]. However, when cross-platform analysis of genetic data is performed, no study has yet delved into how to optimize tumor subtype classification models under the interplay among feature selection methods, normalization methods, and ML algorithms.

Therefore, we here propose a cross-platform data normalization method for tumor subtype prediction based on cross-platform transcriptomic data. We will study how to best select stable genes for normalization and differentially expressed genes (DEG) for classification when models trained on RNA-seq data are used for the prediction of microarray data or vice versa. Then, we will analyze which combined use of normalization methods and supervised ML methods can achieve better tumor subtype prediction. Taking this tumor subtype classification as an example, we hope to provide researchers with a comprehensive normalization strategy for various classification prediction studies based on omic data.

### Dataset Description

To fulfill the experimental requirements, the datasets we chose had to have matched genes present on both microarrays and RNA-seq datasets, and a sufficient number of labeled samples.

The Breast Cancer (BRCA) dataset from The Cancer Genome Atlas (TCGA) include samples examined using both microarray and RNA-seq platforms and well-defined molecular subtypes, which are well suited to be used as class labels for supervised ML models. We restricted the datasets of both platforms to the BRCA tumor samples with corresponding molecular subtype labels. Thus, 520 samples were selected from 597 microarray samples, and 522 samples were selected from 1215 RNA-seq samples. The qualified microarray samples included 96 cases of Basal, 58 cases of Heritage, 231 cases of LumA, 127 cases of LumB, and 8 cases of Normal. The ratio of the number of samples in the largest class to the smallest class is approximated to be 29:1, which is a typical unbalanced dataset. These 520 microarray samples exist in the 522 RNA-seq samples at the same time, and the RNA-seq platform has two more Basal samples.

## 2. Materials and Methods

**Figure 1.**
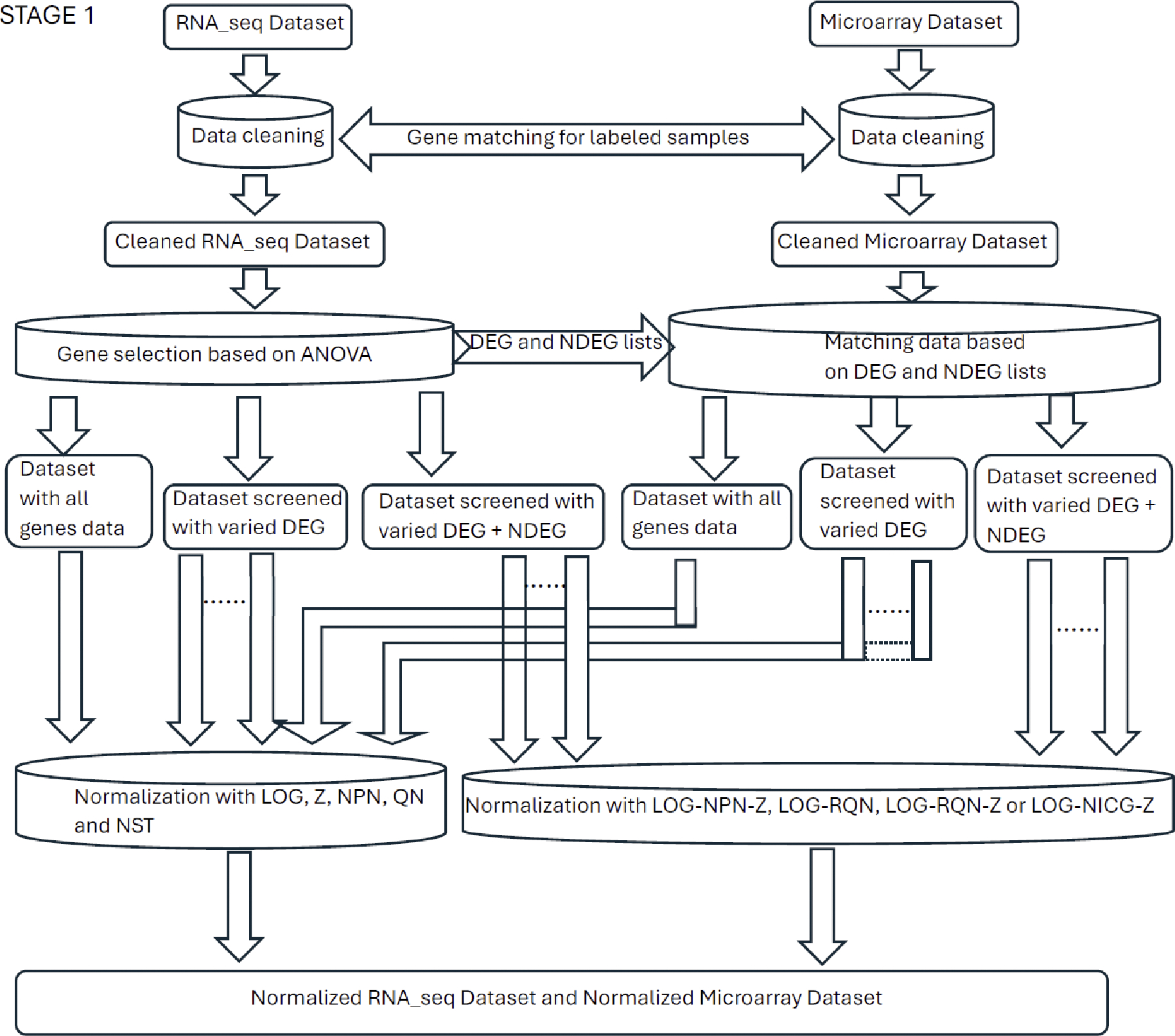
Stage 1 of the framework of the classification strategy: data cleaning, gene selection and normalization (RNA-seq Dataset as training set and Microarray Dataset as testing set).

**Figure 2.**
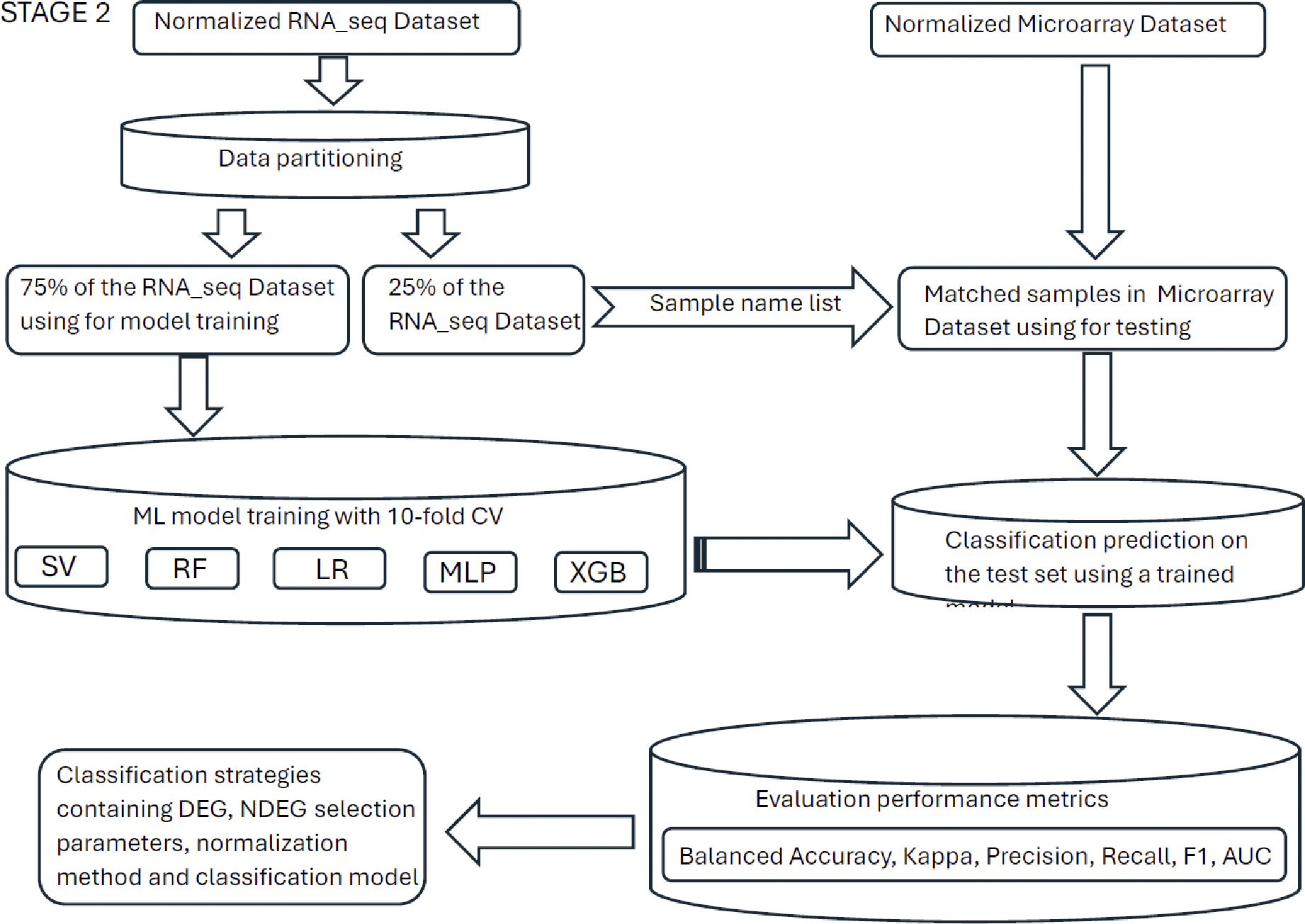
Stage 2 of the framework of the classification strategy: dataset partitioning, classification model training, prediction and classification performance evaluation (RNA-seq Dataset as training set and Microarray Dataset as testing set).

A flowchart for training on RNA-seq data and testing on microarray data has been divided into two stages and shown in Figures 1 and 2 (Supplementary Figures S1 and S2 show a flowchart for training on microarray data and testing on RNA-seq data). The entire process was repeated at least five times. The analysis steps of each process mainly included: data cleaning, gene selection, normalization, dataset partitioning, classification model training, prediction and classification performance evaluation. Python version 3.11.9 64-bit was used for the code implementation. For the convenience of the subsequent narrative, we will refer the model training based on the RNA-seq data and testing based on the microarray data as Model-S, and the model training based on the microarray data and testing based on the RNA-seq data as Model-A.

### 2.1. Data Cleaning

Samples were first screened against the data from both platforms, retaining only those samples with corresponding subtype classification labels. Genes that were present in the datasets of both platforms were retained by gene matching. Then the corresponding genes with missing expression values were removed. After data cleaning, expression values of 15,672 shared genes in the samples with classification labels were subject to analyses.

### 2.2. Gene Selection

Given the challenges posed by high-dimensionality as shown in this study (15,672 genes vs 520 samples), feature selection reduction is often required to improve model performance and interpretability [26,27,29,33,35]. There are several common approaches for feature selection: filtering, wrapping and embedding methods. Here, we performed a one-way ANOVA, a filtering method based on statistical analysis, on the data from each of the two platforms separately.

ANOVA is used to compare between-group variance (differences between category means) and within-group variance (fluctuations within the same category) for data sets with multiple categories to determine if at least one group’s mean is significantly different from the others. The *F*-value is the ratio of two variances and represents the variance of the between-group means compared to the within-group variance. It is used to test the null hypothesis, which states that all group means are equal [45,62,63].

The *F*-value in ANOVA is calculated as follows:

(1) $$F\;=\frac{MSB}{MSW}=\frac{\frac{\sum_{i=1}^{k}n_{i}{\left(\overset{-}{Y_{i}}-\overset{-}{Y}\right)}^{2}}{k-1}}{\frac{\sum_{i=1}^{k}\sum_{j=1}^{n_{i}}{\left(Y_{ij}-\overset{-}{Y_{i}}\right)}^{2}}{N-k}}$$

where MSB (Mean Square Between-group) is the sum of squares between groups divided by k-1, the degrees of freedom between (number of categories minus one), and MSW (Mean Square Within) is the sum of squares within groups divided by N-k, the degrees of freedom within (total sample size minus the number of groups). N is the total number of observations, k is the number of groups, $n_{i}$ is the number of observations in group i, $\overset{-}{Y_{i}}$ is the mean of group i, and $\overset{-}{Y}$ is the overall mean.

A high *F*-value indicates a greater likelihood that the between-group variance is much larger than the within-group variance, suggesting that there are significant differences in means between groups. Genes with high *F*-value are considered suitable for classification and are referred to as differentially expressed genes (DEGs). Conversely, genes with low *F*-value show less significant variation across groups and are considered appropriate reference genes for normalization, termed non-differentially expressed genes (NDEGs).

To follow statistical principles of gene selection, *F*-values are first calculated from gene expression data and sample category labels, then compared with the theoretical values in the *F*-distribution table to determine the *p*-value. The *p*-value represents the probability of observing the current or more extreme *F*-value under the null hypothesis (that all group means are equal). If the *p*-value is less than a preset significance level (e.g., 0.05), the null hypothesis is rejected, indicating that at least one group’s mean is significantly different. By setting different thresholds, the corresponding gene sets can be determined. For example, when the threshold is 0.95, genes with *p* > 0.95 are selected as a set of NDEG for normalization. When the threshold is 0.05, genes with *p* < 0.05 are selected as a set of DEG for classification. The effects of different NDEG and DEG gene sets on the classification prediction results were observed by varying the thresholds.

### 2.3. Data Partitioning

In order to fairly evaluate the prediction performance on data from one platform of a classification model trained on data from another platform, a rational dataset partitioning strategy needs to be designed. Repeated validation and hold-out methods are two commonly used methods for ML model evaluation. Repeated validation refers to evaluating the performance of a model multiple times using different training and test sets, and then taking the average as the final performance estimate. The hold-out method, on the other hand, pre-divides a portion of the dataset as a test set, then uses the training set to train the model and the test set to evaluate the model’s performance. On a small dataset, holding a larger percentage of data for testing may result in insufficient training data, which may affect the model performance, while holding a smaller percentage of data may lead to unstable results, as some important features may not be adequately represented in the test set.

Therefore, we adopt a repeated validation approach here to evaluate the model performance. When we randomly select some samples on the RNA-seq platform for training, then the remaining samples in the microarray dataset that do not overlap with these samples are used for testing, and vice versa. To ensure the fairness of the model evaluation, during the completion of the complete round of analysis shown in the flowchart, the samples constituting the training data and the test set were kept constant throughout the process, regardless of how the gene selection thresholds were varied and how the normalization methods and classifiers were combined. The training data are further randomly divided into training and validation sets to complete the training of the mode.

Since the data itself has five categories (cancer subtypes) with very large non-equilibrium, the data will be divided into training data and test set in the ratio of 75:25 while maintaining the original category ratio. Under Model-S, 75% (390 samples) of the 522 RNA-seq samples were randomly selected. To find the best performing model configuration, the validation was done by *k*-fold cross validation technique with K value considered to be 10. After the training was completed, the samples with the same names as the samples involved in the training were removed from the Microarray data and only the remaining 131 samples that do not overlap constitute the test set for performance evaluation. In Mode-A, 75% (389 samples) of the 520 Microarray data samples were randomly selected to form the training and validation sets, while the corresponding samples in the RNA-seq data were removed, and only the remaining 133 samples that did not overlap were retained to form the test set. When randomly dividing the training data and test set, the proportion of the number of samples in each category was always kept the same as in the raw data set.

### 2.4. Normalization

Among the main steps in the processing of genetic data, normalization is essential and its importance is well recognized. There are many normalization methods, and the choice of which method to use is related to the data and the goal of processing. Here we choose only a few commonly used normalization methods for comparative analysis to refine our processing strategy.

We first investigated the effect of five commonly used normalization methods on data preprocessing on both the full gene data and data screened with DEG selected with different thresholds. These methods include Log2-transformation (LOG), Z-Score transformation (Z), Normal score transformation (NST), Non-parametric normalization (NPN), and Quantile normalization (QN). We then investigated the effects of four reference gene-based normalization methods, including LOG-NPN-Z, LOG-RQN, LOG-RQN-Z, and LOG-NICG-Z (Normalization using internal control genes (NICG)).

- Log2-transformation (LOG) [12,23]
  Genomic data typically exhibit a wide dynamic range and right-skewed distribution. Logarithmic transformation reduces both the dynamic range and skewness, thereby promoting symmetry and approximate normality. This helps the data better meet the assumptions of downstream statistical analyses. To avoid issues with zero or near-zero values, a small constant (e.g., 1) is usually added to each value before applying the logarithm.
- Z-Score transformation (Z) [12,23]
  Z-score transformation is a widely used normalization method that scales features to have a mean of 0 and a standard deviation of 1. For each gene or trait, the mean (μ) and standard deviation (σ) across samples are calculated, and each value (x) is standardized using the formula (x−μ)/σ. This transformation allows different features to be placed on a comparable scale, which is particularly useful in ML and statistical inference.
- Normal score transformation (NST) [64]
  Normal Score Transformation is a specific type of non-parametric method designed to map observed data ranks into values that follow a standard normal distribution. First, data are ranked within each feature across samples. These ranks are then converted into cumulative probabilities (percentiles), which are in turn transformed into z-scores using the inverse cumulative distribution function (CDF) of the standard normal distribution. NST ensures that the transformed data closely approximate a Gaussian distribution, which facilitates the use of statistical tests and models that assume normality.
- Non-parametric normalization (NPN) [65]
  Non-parametric normalization is a distribution-free transformation approach widely used in high-dimensional biological data. It operates by ranking the data within each feature (gene), converting these ranks into percentiles, and then mapping them onto a reference distribution such as the standard normal. Because it does not assume a specific underlying data distribution, NPN is robust to outliers, skewness, and heteroscedasticity. This makes it especially suitable for datasets where technical variation dominates and normality assumptions may not hold. NPN is flexible in that the final mapping step can be adapted to any desired distribution, or even omitted if only rank information is needed.
  While both NST and NPN involve ranking data and applying distributional transformations, NST can be viewed as a specialized form of NPN that explicitly enforces a standard normal output using the inverse normal CDF. This makes NST particularly suitable for parametric statistical analysis. In contrast, NPN provides a more general, distribution-free normalization framework that offers greater flexibility—for example, allowing mapping to any reference distribution or omitting the final transformation step altogether—but with less imposed statistical structure.
- Quantile normalization (QN) [12]
  Quantile normalization assumes that the overall distribution of gene expression is similar across samples. It aligns the expression values across samples by sorting them and replacing each value with the average value for the corresponding quantile across all samples. This reduces the impact of outliers and technical noise, and improves comparability between samples. QN is especially effective when technical variation dominates over biological differences.
- Reference-based Quantile Normalization (RQN)
  Reference-based quantile normalization (RQN) is a variant of QN that uses a selected set of reference genes—often non-differentially expressed genes (NDEGs)—to define the normalization target. The expression values of these stable genes across samples are used to compute quantile averages, which are then used to normalize all genes. RQN is particularly useful in cross-platform or cross-batch studies, where relying on the full set of genes might introduce unwanted bias.
- LOG-RQN
  LOG-RQN will further do RQN on the LOG-processed data.
- LOG-RQN-Z
  LOG-RQN-Z will further do RQN on the LOG-processed data before doing a Z transformation.
- LOG-NPN-Z
  LOG-NPN-Z further applies a Non-Parametric Normalization (NPN) step to log-transformed data before performing Z-score normalization. Unlike the conventional NPN method, this approach uses a preselected set of reference genes (NDEGs) to define a standard expression distribution. Each sample’s gene expression values are then percentile-mapped against this reference distribution, effectively reducing systematic variation across samples.
- Normalization using internal control genes (NICG) [66]
  This method uses endogenous control genes, also known as housekeeping genes, to normalize gene expression data. These genes are assumed to be stably expressed across different biological conditions. The average expression level of the internal control genes in each sample is computed and used as a scaling factor to normalize the expression levels of all genes. This approach compensates for technical variability and enhances data comparability, particularly when global normalization assumptions are not appropriate.
- LOG-NICG-Z
  LOG-NICG applies NICG processing to log-transformed data before performing Z-score normalization. During the NICG step, the selected NDEGs are used as endogenous control genes.

After applying the same normalization methods to the training data and test set, different classification learning models are used for training and testing. The impact of different normalization methods during data analysis was evaluated by comparing these results with that of direct classification prediction on the raw data.

### 2.5. ML Models

Based on different training sets, we trained five common classifiers based on common ML algorithms: Multilayer Perceptron (MLP), Extreme Gradient Boosting (XGB), Logistic Regression (LR), linear Support Vector Machine (SVM), and Random Forest (RF). The five classification models presented here are all commonly used in practice, but each has different characteristics that make them suitable for comparing the interaction between dataset characteristics and models.

SVM [67] is a supervised ML algorithm that classifies data by finding the optimal hyperplane. It can be used for nonlinear problems by applying kernel tricks. SVM is particularly suitable for classification of small and medium-sized complex datasets, and handles high-dimensional data and nonlinear problems well.

LR [68] is a linear model that effectively reduces the complexity of the model and the risk of overfitting by introducing L1 regularization for feature selection. It is suitable for datasets with a large number of irrelevant features because it can help select the most useful features through sparse solution, thus improving the generalization ability of the model.

RF [69] is an integrated decision tree-based learning model that enhances the generalization ability of the model by introducing random feature selection. It is particularly effective for datasets with nonlinear, outliers and complex interactions between features.

XGB [70,71] is a high-performance model based on gradient boosting decision trees and shares similarities with RF. It optimizes the regularization of the model and effectively prevents overfitting. It is ideally suited for sparse data and excels in both classification and regression problems, and performs particularly well with structured datasets.

In contrast, MLP [72,73] is a forward deep/neural-network learning model containing one or more hidden layers. It is well-suited to the approximation of complex functions in pattern recognition and classification tasks, and exhibits robust learning capabilities for nonlinear relationships and highly complex patterns in data.

To address the issue of class imbalance, we employed stratified 10-fold cross-validation in all experiments to ensure representative class distributions within each fold. GridSearchCV was used for hyperparameter tuning, with the weighted F1 score (f1_weighted) as the evaluation metric to improve performance on minority classes.

For the five models under study, we designed structured hyperparameter grids that covered key dimensions such as model complexity, regularization strength, and training stability.

- LR: We tuned the regularization strength C across a log-spaced range (np.logspace(−2, 1, 5)) and tested two common solvers (liblinear and lbfgs), both of which are suitable for small to medium datasets and compatible with L2 regularization.
- SVM: We explored both linear and RBF kernels. For RBF, we tuned gamma values (including ‘scale’, 0.01, 0.1) to control model flexibility. To ensure training convergence, we set max_iter = 1000 and enabled probability = True to allow probability-based predictions.
- RF: We set n_estimators to [100, 200, 300] to avoid instability in small-tree ensembles. We tuned max_depth ([3, 4, 6]), min_samples_split, min_samples_leaf ([2, 5, 10]), and max_features (‘sqrt’, ‘log2’) to enhance tree diversity and generalization.
- MLP: We tested several network structures ((100,), (100, 30), (100, 50)), multiple solvers (‘adam’, ‘sgd’, ‘lbfgs’), and L2 regularization strengths (alpha = 0.0001, 0.001). We also enabled early_stopping = True to mitigate overfitting and fixed max_iter = 500.
- XGB: We tuned n_estimators ([100, 200, 300]), learning_rate ([0.05, 0.1, 0.3]), max_depth ([3, 4, 6]), subsample ([0.6, 0.8, 1.0]), colsample_bytree ([0.6, 0.8, 1.0]), and gamma ([0, 0.1, 0.5]) to balance convergence speed, regularization, and ensemble diversity.

Overall, the hyperparameter search space was designed to balance flexibility and generalizability while keeping the computational cost feasible.

### 2.6. Evaluation of Classification Performance

Each model was trained on the training set using 10-fold cross-validation and subsequently evaluated on an independent cross-platform test set. The entire process of model training and independent testing was repeated five times, with the data re-partitioned in each iteration according to the aforementioned strategy. Due to the multi-class and unbalanced nature of the data in this study, Balanced Accuracy and the Kappa statistic (Kappa), in addition to F1 Score (F1), Area Under the Curve (AUC), sensitivity (Recall), and specificity, were used to evaluate classification performance based on the test set [32,63,74–77]. Finally, all performance metrics were averaged across the five independent test results to provide a comprehensive assessment of the model’s generalization ability on external datasets. All model performance results presented in the figures and tables of this paper are obtained from the independent test set.

The kappa statistic is a measure of classification accuracy that takes into account unbalanced data and chance agreement. The kappa is a statistic that compares the observed accuracy with the performance of a random classifier. It is calculated as Equation (2).

(2) $$K\;=\;(P_{o}\;-\;P_{e})/(1\;-\;P_{e}),$$

where $P_{o}$ is the observed agreement (actual accuracy) and $P_{e}$ is the expected agreement under random classification. The kappa value typically ranges from −1 to 1, with 0 denoting random accuracy and 1 denoting perfect agreement.

Balanced Accuracy is a metric that accounts for class imbalance and represents the average accuracy for each class. In the case of an unbalanced dataset, the overall accuracy may be high, despite the fact that the predictions for a few classes may be inaccurate. Balanced Accuracy provides a fairer assessment of the model’s performance across all classes. It is calculated as:

(3) $$Balanced\;Accuracy=(1/n)\sum_{i=1}^{n}\left(\frac{\mathrm{True}\;\mathrm{Positives}\;i}{\mathrm{Total}\;\mathrm{Class}\;i}\right),$$

where n is the number of classes.

As a typical genetic dataset, BRCA is an unbalanced dataset. Using only traditional accuracy tends to overemphasize the impact of dominant categories. The Kappa value is a measure of agreement between observed and randomized accuracy, so randomized accuracy is considered in categorical accuracy. Instead of simply calculating the total percentage of correct classifications, Balanced Accuracy is the average of the recall (or true rate) of all categories. This ensures that all categories are equally important regardless of size, thus providing a score for classifiers that performs fairly on each category. Consequently, the Kappa value is more appropriate for scenarios where random guessing performance needs to be considered, whereas BalancedAccuracy is more suitable for datasets with an imbalanced distribution, where each category must be of equal importance. The combination of BalancedAccuracy and Kappa value provides a more balanced and accurate assessment of model performance across all categories. In this way, any potential bias in favor of a particular category can be identified.

Based on the combination of BalancedAccuracy and Kappa value, we design the formula shown in Equation (4) to calculate the model evaluation value ($E_{value}$) for model selection.

(4) $$E_{value}=-100\;\left(Kappa*Balanced\;Accuracy\right)*\log(\sigma_{Kappa}*\;\sigma_{Balanced\;Accuracy})$$

where $\sigma_{Kappa}$ and $\sigma_{Balanced\;Accuracy}\;$are the variance of the corresponding Kappa and BalancedAccuracy obtained from multiple repetitions of the experiment, respectively, which can measure the robustness of the model. A large $E_{value}$ corresponds to a better model performance.

## 3. Results

We repeated the processing flow in 5 times to obtain average performance metrics (Figures 1 and 2, Supplementary Figures S1 and S2). Regardless of the perspective, the model classification performance obtained in Model-S mode is generally better than that obtained in Model-A mode, which stems from some technical methodological, data characterization, and application differences between the datasets obtained by the two platforms. RNA-seq provides more comprehensive and precise transcriptome information.

Examining the performance metrics corresponding to Model-S or Model-A in Supplementary Tables S1 and S2 separately, we find that the classification performance metrics show different trends with the changes of DEG or NDEG genes, regardless of whether we observe the performance of different classifiers under the same normalization method or the performance of different normalization methods under the same classifier. This suggests that gene selection, normalization methods and supervised ML classifiers need to be analyzed together.

### 3.1. Results on the Original Data

**Figure 3.**
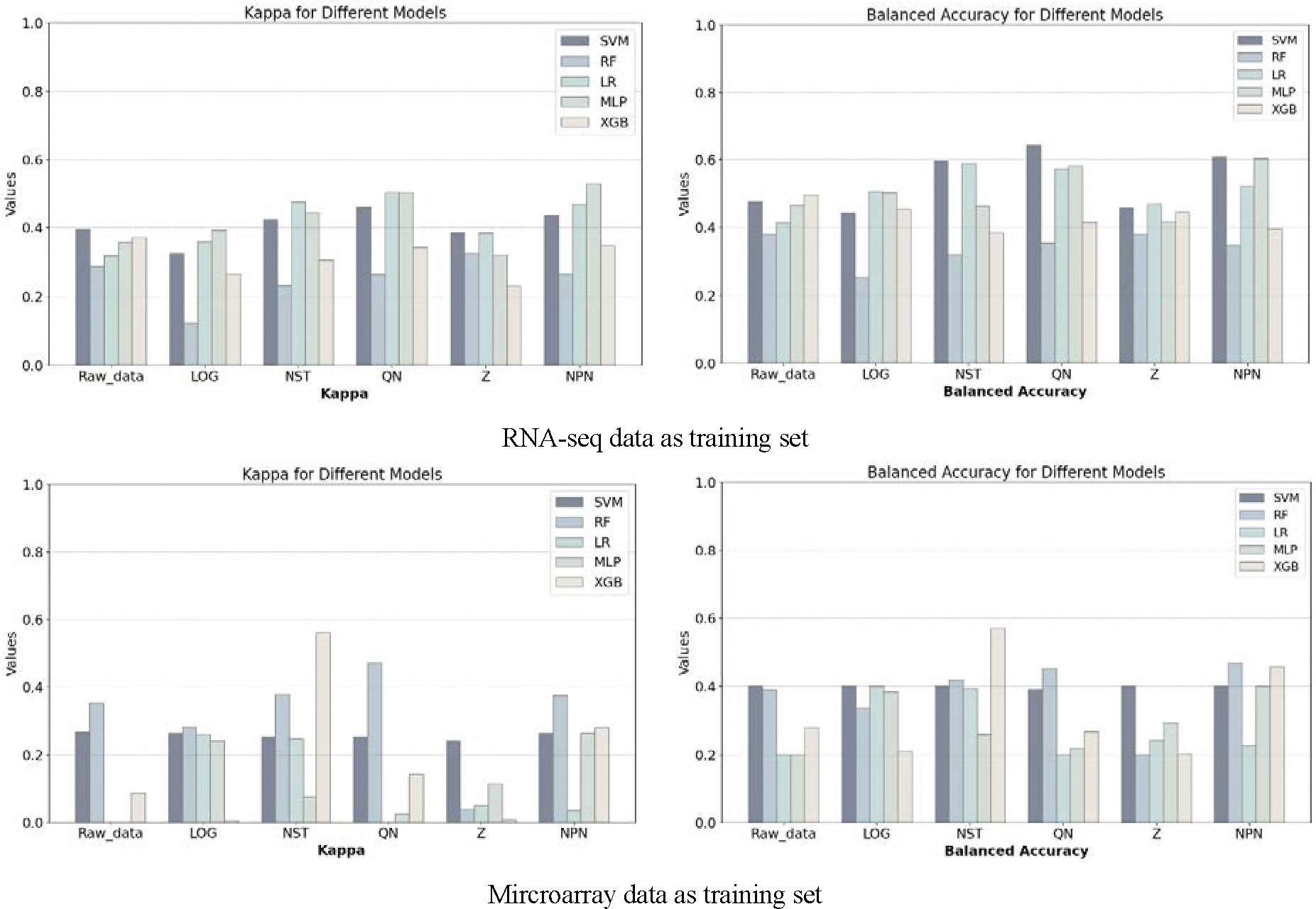
The classification performance results obtained on the original data.

We consider the performance of ML models on Raw_data (i.e., without gene selection) as the baseline. All expression values corresponding to the 15,672 genes shared by the two data platforms are directly used for the analysis to observe the performance of the five classifiers in the Raw_data or the data processed by different normalization methods. The performance results (Figure 3) show that the five different classification models present completely different patterns of change on different datasets. The classifiers do not work at all in some cases. For example, in Model-A, MLP and LR have almost no effect on Raw-data, and the corresponding kappa value is close to 0.

Although MLP, SVM (Model-S) or XGB (Model-A) generally perform better than the others in general, and especially the models sometimes show some classification improvement on data processed by the NPN, QN, and NST normalization methods, from the point of view of practical application, both the kappa and the Balanced Accuracy are not satisfactory. Among them, the $E_{value}$ is calculated according to Equation 4 to evaluate the model performance. Under Model-S, when classifying on Raw_data directly, XGB received a relatively high evaluation due to having the highest $E_{value}$ (99.754), with a corresponding Balanced Accuracy of 0.496 and a Kappa of 0.372. After normalization, the best-performing combination was QN and SVM, with an $E_{value}$ of 198.072, Balanced Accuracy of 0.644 and a Kappa of 0.460. For Model-A on Raw_data, RF performed the best, with an $E_{value}$ of 72.637, Balanced Accuracy of 0.389 and Kappa of 0.352. After normalization and classification, the superior performance was achieved by the combination of NST and XGB, with an $E_{value}$ of 223.410, Balanced Accuracy of 0.571 and Kappa of 0.560.

### 3.2. Results on Data Selected by DEG

**Figure 4.**
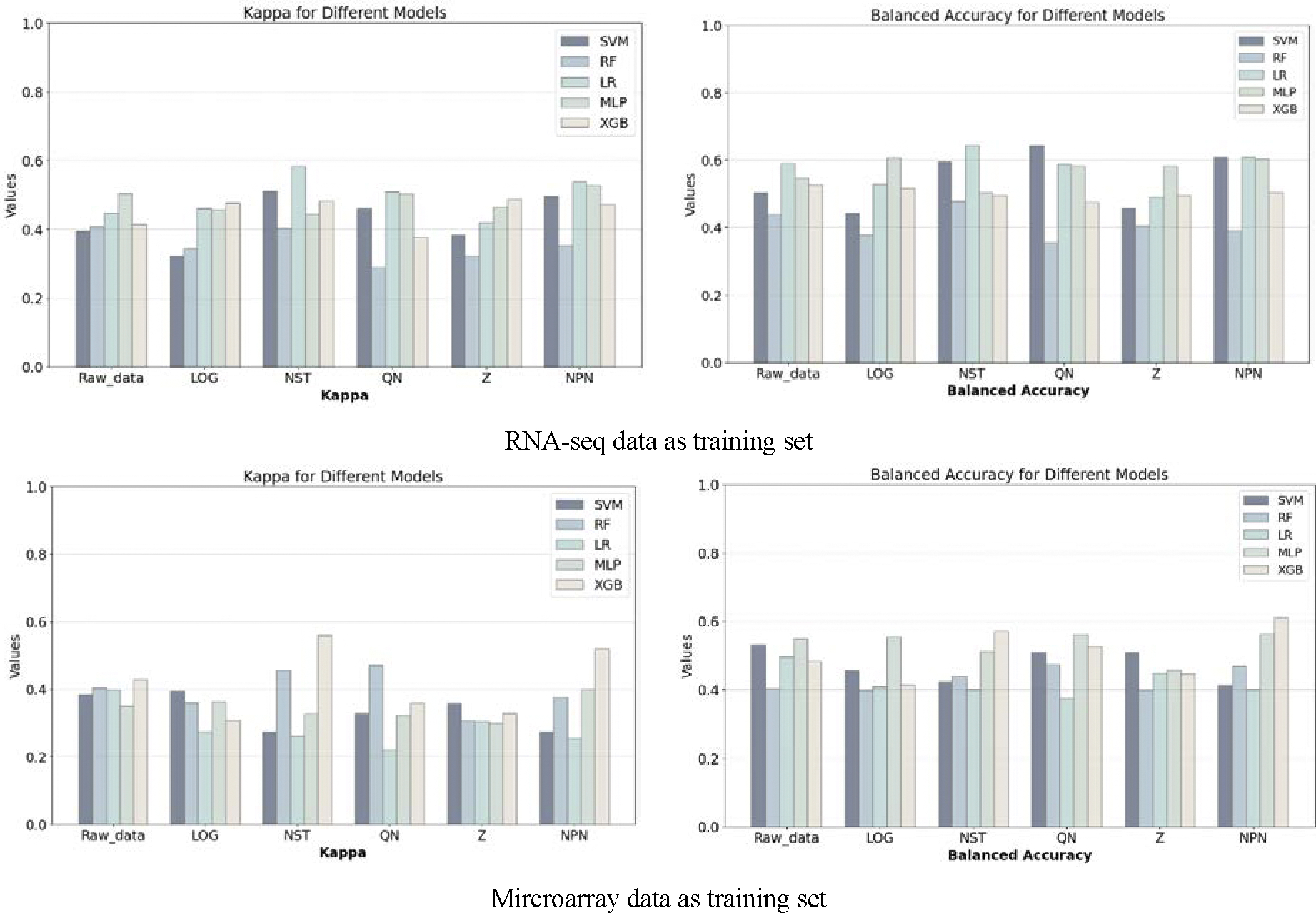
The best classification performance results obtained on data selected by DEG genes. Multilayer Perceptron (MLP), Extreme Gradient Boosting (XGBoost), Logistic Regression (LR), linear Support Vector Machine (SVM), and Random Forest (RF).

Next, we used the gene selection strategy described above to select the expression data corresponding to the DEG with *p*-values below a certain threshold, and analyzed the data after normalization with LOG, NST, QN, Z and NPN, respectively. The DEG gene selection threshold varied gradually from 0.001 to 0.1 (Supplementary Tables S1 and S2). The model classification results obtained at different thresholds were compared, where the optimal performance is shown in Figure 4.

It appears that the classification performance of the five classifiers does not show a monotonous upward or downward trend with increasing DEG thresholds for data processed by any of the normalization methods (Supplementary Tables S1 and S2). In the vast majority of settings, the classification results are not satisfactory. Compared to the setting where normalization and classification are done directly on the Raw_data, most of the settings do not show any improvement, and in some settings, the classifier does not work at all (kappa values are 0 or even negative).

Interestingly, even when randomly dividing the training data and the test set according to the proportion of each category in the original dataset, the imbalance of the samples can lead to very different results in the repeated experiments. For example, when using MLP as a classifier with the DEG selection threshold set to 0.03 and using Z as the normalization method, the highest accuracy is 0.70415 and the lowest accuracy is 0.4623 in repeated experiments, which suggests that repartitioning leads to changes in the data, resulting in large fluctuation in the models’ performance. The finding implies the model is less robust. This is exactly the reason why we designed the $E_{value}$ that combines the mean and the variance of the Kappa and Balance Accuracy obtained from several repetitive experiments when selecting the model based on the evaluation metrics.

For the datasets selected from the DEG with different thresholds, the results of Model-A using SVM seems overall more stable than other ML algorithms (Table 1), as shown by its smaller standard deviation and coefficient of variation. On the other hand, the other classifiers show large fluctuations with the change of gene selection thresholds, and such fluctuations are not consistent across the data processed by various normalization methods. For example, the MLP classifier fluctuates more in Raw_data and NST-processed data, while the RF and XGB models fluctuate more in QN-processed data. Strikingly, DEG selection seems not associated with significant improvement in classification performance as compared with that of Raw_data (i.e., no gene selection). Indeed, better results may be achieved due to comprehensive information when all gene data are involved in model training.

For the Model-S, the performance of each classifier fluctuates dramatically with the threshold. Although the SVM and MLP are slightly better overall, there is also no significant improvement in the classification performance compared to when the gene selection strategy is not used. These data suggest that normalization method and DEG selection alone may not improve the overall performance of ML algorithms.

### 3.3. Results on Data Selected by NDEG and DEG

Subsequently, we used a gene selection strategy to select NDEG with *p* values above a certain threshold. Four reference gene-based normalization methods, including LOG-NPN-Z, LOG-RQN, LOG-RQN-Z, and LOG-NICG-Z, were used to process the corresponding gene expression data jointly selected from the NDEG and the DEG, including the training data and the test set, and then used the five classification models mentioned above to perform classification training and testing (Supplementary Tables S3–S6). Among them, the NDEGs were used as the reference genes required for these normalization methods.

**Figure 5.**
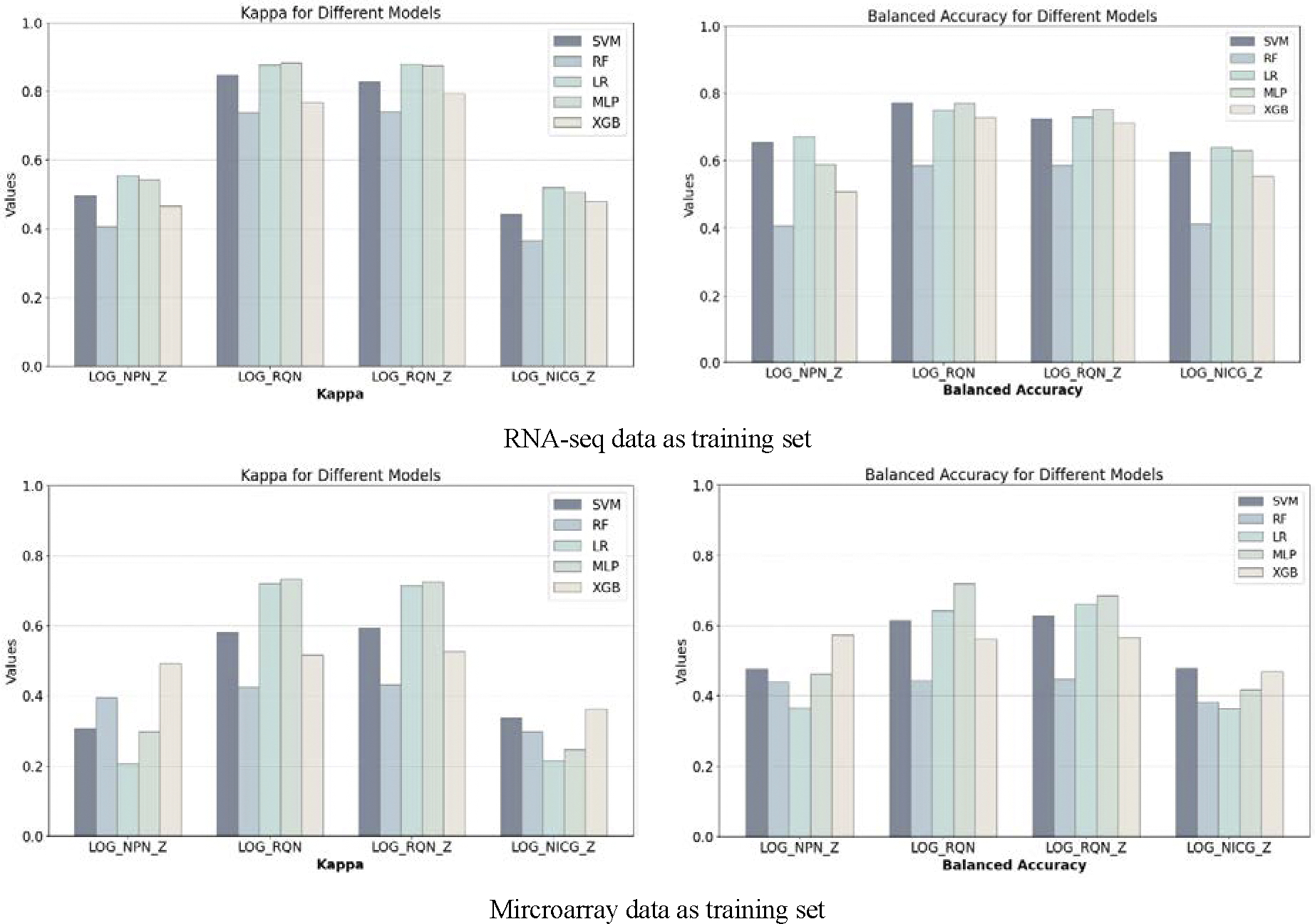
The best classification performance results obtained on data selected by DEG and NDEG genes.

The optimal values in each matrix showed that the performance of both Model-S and Model-A was significantly improved (Figure 5). At the same time, the average performances (reported as Mean ± Standard Deviation) of each model in the corresponding Figure 5 were shown in Supplementary Tables S7–S10. Compared with the classification results on data selected using NDEG and DEG genes, we noted the following findings.

First, for Model-S, using the data normalized with LOG-RQN or LOG-RQN-Z, MLP, LR and SVM classifiers can significantly improve the classification performance within the preset range of NDEG and DEG thresholds. Among them, MLP has a Kappa average of over 0.83 and an accuracy mean of 0.700 (Figure 5). Further observation of the model’s performance on the LOG-RQN and LOG-RQN-Z processed data also reveals that the surfaces corresponding to each of the key metrics in the categorization performance fluctuate considerably as the NDEG or DEG thresholds are altered, with peaks occurring at very different locations in the tuning matrix (Table 2). It is important to note that the NDEG genes or DEG gene thresholds change steps are not consistent here (Table 2). For example, the MLP classifier reaches a maximum classification Balanced Accuracy of 0.771 at a NDEG gene threshold of 0.98 and a DEG gene threshold of 0.07, a maximum classification Kappa of 0.883 at a NDEG gene threshold of 0.90 and a DEG gene threshold of 0.003. The SVM classifier reaches a maximum classification Balanced Accuracy of 0.773 at a NDEG threshold of 0.90 and a DEG gene threshold of 0.005, a maximum classification Kappa of 0.829 at a NDEG threshold of 0.98 and a DEG threshold of 0.008. This indicates that it is more reasonable to determine the optimal model based on the model performance matrices obtained from the NDEG and DEG gene threshold changes. They also suggest that relying solely on a single traditional performance metric to select a model can be biased.

Second, for Model-A, the results are basically similar. MLP, LR and SVM classifiers perform better on data processed with LOG-RQN or LOG-RQN-Z, but the RF performance is poorer, even worse than the case without the NDEG group. The overall effect of MLP is relatively better and more stable, with the highest kappa value of 0.734 and the highest Balanced Accuracy of 0.718, and the fluctuation of the classification effect with the change of NDEG gene thresholds and the change of DEG gene thresholds is not large (Standard Deviation less than 0.04). The effect of LR is more stable, but the optimal performance is not as prominent as that of MLP. The fluctuation of SVM is relatively large, and the Standard Deviation value seems to be greater than 0.6. For the data under the action of LOG-NPN-Z and LOG-NICG-Z, the overall effect is unsatisfactory, in which XGB outperforms the others.

Third, the data-normalization method may influence ML performance. For example, ML performance on the data normalized with LOG-RQN and LOG-RQN-Z has obvious improvements, but that on the data normalized with LOG-NPN-Z and LOG-NICG-Z does not (Supplementary Tables S3–S6).

## 4. Discussion

### 4.1. Comparison of Kappa and Balanced Accuracy

Balanced Accuracy and Kappa statistic can show similar trends in this study, but sometimes not. When the Kappa value is very low yet the Balanced Accuracy is relatively high, an ML algorithms’ overall performance is not significantly improved over random sampling despite the model’s improved performance on each category. For example, when the NDEG threshold is 0.85 and the DEG threshold is 0.03, the normalization method is LOG-RQN, and XGB is the classification model, the Kappa value obtained averages 0.354 in Model-A, and the Balanced Accuracy averages 0.531. The reason for this may be that we randomly split the training, validation, and test sets by keeping the number of samples in each of the five categories the same as the raw data, which still leaves the data severely unbalanced. Once the model performs well on the main categories, which pushes up the Balanced Accuracy, the overall consistency prediction (as measured by the Kappa value) decreases due to poor performance on the categories with fewer samples. Overall, performance metrics in this case are generally not particularly impressive and the results obtained in repeated experiments vary relatively widely.

We also observed the scenarios of high Kappa values but low balanced accuracies (e.g., in Model-S, when the NDEG threshold was 0.92, the DEG threshold was 0.03, the normalization method was LOG-RQN, and the classification model was LR, the obtained Kappa values averaged 0.814 and the Balanced Accuracies averaged 0.667), which may also stem from the extreme lack of data balancing. Balanced Accuracy reflects the average of the accuracies for each category. If the model performs poorly on any of the categories, it can significantly reduce the Balanced Accuracy, which includes cases where predictions are correct on categories with small sample sizes and can be poorly predicted on major categories with large sample sizes. In this case, the overall consistency (*Po*) may still be high, and the model’s overall predictions perform better than the random predictions, thus improving the Kappa value.

Therefore, when comparing the classification performance of various models, one should not rely solely on a single performance metric. We recommend using the $E_{value}$ calculated using Equation 4. Models with higher mean and lower variance of Balanced Accuracy and Kappa obtained from multiple repetitive experiments should be regarded as high-performing models. Based on the $E_{value}$, we can identify the combination conditions associated with these high-performing models. In Model-S, the highest $E_{value}$ (405.492) corresponds to the model built with an NDEG threshold of 0.85, a DEG threshold of 0.07, the normalization method LOG-RQN-Z, and the MLP classifier. The classification results show an average Balanced Accuracy of 0.752 and an average Kappa value of 0.875. As shown in Supplementary Tables S7 and S8, neither the average Kappa or the average Balanced Accuracy is the highest in this case. In Model-A, the highest $E_{value}$ (311.003) corresponds to the model defined by an NDEG threshold of 0.90, a DEG threshold of 0.005, the LOG-RQN normalization method, and the MLP classifier. This model achieved an average Balanced Accuracy of 0.707 and an average Kappa value of 0.734. Supplementary Tables S9 and S10 shows that the average Kappa is the highest, while the Balanced Accuracy is not.

### 4.2. Thresholds in Gene Selection Strategies

We used the F-values from the ANOVA to determine the *p*-values according to the F-distribution table correspondingly and used this as a threshold to achieve the selection of DEG and NDEG. The gene selection strategy allows for narrowing down the range of DEG used for classification and identifying the core NDEG for normalization. Interestingly, as the range of DEG is narrowed, the performance of the classification model may not improve. Even when all gene data are used for classification, better results can still be achieved. In contrast, addition of NDEG could significantly improve classification performance. Therefore, NDEG may play a more significant role than DEG in improving ML performance. However, since there may be considerable redundancy in (or association among) DEG and our gene selection strategy may not be best optimized, it is probably premature to completely exclude the benefits of using DEG.

Essentially, hypothesis testing is a statistical method that calculates the probability of the strength of evidence for or against the null/original hypothesis (i.e., no difference or no change) based on the sample data, which is ultimately summarized into a single value, the *p* value. A cut-off value (cut-off) of 0.05 and 0.95 is often chosen in various studies, which seems arbitrary and merely an empirically generated convention. In fact, these values are not universal. For example, a stricter cut-off value, such as 0.01, should be used to reach the best ML performance. Indeed, our study show that the thresholds of NDEG and DEG selection for the best model corresponding to Model-A are 0.90 and 0.005, respectively, and the thresholds of NDEG and DEG selection for the best model corresponding to Model-S are 0.85 and 0.07, respectively. Therefore, it seems necessary to find the proper thresholds on the basis of the data and the model in the course of the study.

We also find that when LOG-RQN or LOG-RQN-Z is selected as the normalization method and MLP is selected as the classification method, the classification performance corresponding to different combinations of thresholds for NDEG and DEG shows a relatively stable effect. This suggests that under the premise of optimal selection of normalization methods and classification models, changes in the thresholds of NDEG and DEG selection have relatively limited effects on the final classification performance. Among the three approaches of normalization method, classification model and gene selection strategy in this experiment, the normalization method and classification model currently appear to play a more decisive role than gene selection strategy.

The number of DEGs selected based on *p*-values in our experiments is very large. First, the gene screening strategy in this paper only considered the variability of the features in the category but did not account for the correlation of the features, which contributed to the large number of selected DEGs. Additionally, this partly stems from the high-dimensional nature of the raw data itself (i.e., the number of genes is much larger than the number of samples), which increases the probability of false positives in statistical testing. It may also result from the potential technical variation, noise, or batch effect in the dataset, all of which can affect the outcome of statistical tests. More importantly, it reflects the skewed distribution characteristics of the data and reaffirms that the distribution of gene expression data often do not follow a normal distribution. Therefore, when using traditional methods such as Student *t*-test or ANOVA, the assumption of normality may not hold, leading to erroneous results.

Feature dimensionality reduction is indeed a critical component of our work. In our previous studies, depending on research goals, we have explored various dimensionality reduction methods, including feature importance from RF and XGB, LR, principal component analysis (PCA), and reference-based gene filtering [33,78–80]. Each method shows strengths and limitations depending on the specific task. In this study, we adopted a one-way ANOVA-based gene screening strategy. Its advantage lies in its ability to improve model performance while preserving the original identity of each gene feature, facilitating subsequent biological interpretation. In contrast, although PCA performs well in dimensionality reduction tasks, it transforms the original feature space into principal components through linear mapping, where each principal component is a linear combination of the original features. As a result, the retained new features no longer correspond to specific genes, making it difficult to interpret the biological significance of individual features.

We also attempted to explore the impact of alternative feature selection methods on model performance, including RF, XGB and PCA. Compared to LR, RF and XGB provide feature importance rankings and require manually setting a threshold to select top-ranked features, while PCA also requires manual determination of the number of dimensions to retain during dimensionality reduction. In contrast, LR with elastic net regularization performs automatic feature selection during training by shrinking coefficients through regularization. Only features with non-zero coefficients are retained. This feature selection process is entirely determined by the model itself. Therefore, we conducted an exploratory analysis on Model_S using LR with elastic net regularization for feature selection, and then trained and tested models based on the DEG and NDEG matrices generated by this method to evaluate the method’s feasibility and performances. Performance metrics of these top-performing models—those achieving the highest Balanced Accuracy—for each DEG and NDEG matrix are summarized in Supplementary Table S11. Although this method significantly reduced the number of selected features, model performance also declined (with the best Kappa around 0.734 and the best Balanced Accuracy around 0.707). This finding aligns with the general understanding of dimensionality reduction: while reducing the number of features can effectively lower computational burden and sometimes improves performance, it also inevitably decreases the amount of information available to the model, potentially compromising classification performance. These results further highlight our motivation to balance model interpretability and predictive performance through gene selection strategies. They also support the validity of the gene selection approach we proposed.

To address concerns about inflated false discovery rates (FDR) in high-dimensional settings, we conducted an exploratory study in Model-S by applying FDR-based multiple testing correction to the results of the one-way ANOVA. When applying an FDR threshold of 0.05, the absolute change in the number of DEGs and NDEGs was relatively small (see Supplementary Table S12 for comparison). Due to computational constraints, we performed only a single round of experiments using the FDR-adjusted DEG and NDEG sets across all classifiers presented before. The performance metrics of the top-performing models (based on highest Balanced Accuracy) are reported in Supplementary Table S13. Although the absolute changes in DEG and NDEG numbers were modest, the reduction in NDEGs may have had a disproportionate effect on normalization quality and classification performance. We observed a corresponding decrease in classification performance with the best Kappa around 0.724 and the best Balanced Accuracy around 0.679. This suggests that moderately relaxing the significance threshold to retain weakly informative features may be more beneficial for model performance than strictly enforcing statistical significance, at least with the use of FDR-based correction.

Therefore, in future work, we plan to enhance our gene screening strategy using two methods. First, for multiple testing correction, we will consider more robust FDR control methods that account for inter-gene correlation, such as the Benjamini–Yekutieli procedure (suitable for dependency structures) or the q-value approach, to more accurately control the false discovery rate. Second, considering that some data may not conform to any explicit (finite-parametric) distributional form, we also plan to introduce non-parametric methods (e.g., the Kruskal–Wallis test or permutation-based inference) to improve the adaptability and robustness of our gene selection process.

### 4.3. Impact of Normalization on Model Performance

When selecting and designing models, the potential impact of data preprocessing steps on the performance of the final model needs to be considered. Appropriate data preprocessing can improve model performance. For the RNA-Seq by Expectation-Maximization (RSEM) counts of BRCA used in this study, we also found significant differences even when using the same classification model for data processed by different normalization methods.

Comprehensively comparing the classification performance of different classification models on data processed by various normalization methods with and without NDEG, we find that the LOG and Z perform relatively poorly, while QN and NPN yield more stable results when NDEG is not used, consistent with prior reports [12]. After incorporating NDEG selection, MLP, LR and SVM all show improved performance on data processed by LOG-RQN and LOG-RQN-Z methods. However, the impact of NDEG and DEG selection appears to be less critical than the choice of normalization methods. We also observe that Z-transformation, when used in conjunction with robust preprocessing, contributes positively to model performance. Conversely, models trained on data processed by LOG-NPN-Z and LOG-NICG-Z underperform, despite the use of reference genes.

We attribute the underperformance of LOG and Z methods to the wide variance and inconsistency of cross-platform gene expression data, as well as the presence of noise and extreme values. NICG, which depends heavily on the stability of the selected internal control genes, may be compromised when those genes are not reliably expressed. NST addresses these challenges by mapping ranked data to a standard normal distribution using the inverse normal CDF. NPN similarly uses rank-based percentile mapping but supports more flexible target distributions, making it robust to outliers, skewness, and heteroscedasticity. QN, a non-parametric method, aligns the expression distribution across samples and is especially effective in handling sparsity and preserving feature relationships—advantages that benefit models like SVM and MLP.

Parametric methods such as Z-score normalization assume that the data fit an approximate Gaussian distribution and apply linear transformations based on the sample mean and standard deviation. These approaches perform well with large, symmetric datasets, but they are highly sensitive to skews and outliers. In contrast, non-parametric methods like QN, NST, and NPN do not rely on distributional assumptions and remain effective under skewed, heavy-tailed, or noisy data—conditions often encountered in transcriptomic analysis. Technically, NST can be viewed as a special case of NPN, where transformation is explicitly mapped to the standard normal distribution via the inverse CDF. NPN, however, offers greater flexibility by supporting custom or omitted target distributions.

In our experiments, parametric methods like Z-score underperformed unless preceded by more robust normalization steps, while non-parametric methods such as QN and LOG-RQN yielded more stable results across models. Taken together, these observations reinforce our methodological choice to prioritize non-parametric normalization strategies such as QN, NPN, and NST, which are more robust to skewness, extreme values, and batch effects commonly found in cross-platform transcriptomic data.

Furthermore, consistent with prior reports [22,45,81–85], our gene selection strategy is also based on ANOVA. Although ANOVA assumes normality of residuals for valid F-test statistics, it is generally robust to modest deviations from this assumption. In large sample settings, the Central Limit Theorem justifies the continued use of such methods, even when the raw data deviate from normality. However, in small-sample, high-dimensional transcriptomic studies, violations of normality may significantly impact the accuracy of statistical inference. We believe this limitation partly explains why the DEG-based filtering strategy did not substantially improve classification performance in our study. Future work will explore non-parametric (distribution-free) feature selection techniques to better capture core biological signals and reduce potential bias.

In summary, while normalization benefits all models, its impact varies substantially depending on the method chosen and the learning model applied. Proper selection of normalization techniques—based on empirical data characteristics rather than theoretical assumptions alone—is essential to maximize model generalizability, robustness, and biological interpretability.

### 4.4. Impact of Classification Models

Different ML models often yield varying performance on the same dataset due to their distinct learning mechanisms and ways of processing data features. In our experimental results, we found a situation where the LR and MLP models performed the best, while the SVM performance fluctuated and the XGB and RF performed poorly. The finding may be attributable to the specific characteristics of the dataset and the mechanisms by which each of these models interacts with these characteristics.

Possible factors for this phenomenon include:

1. The dimensionality and sparsity of the data
  A dataset may contain many irrelevant or redundant features. LR, which implements feature selection through L1 regularization, tends to perform well on datasets with high dimensionality and low correlation between features [86]. If the dataset contains many irrelevant features or noise, LR can effectively identify and compress these unimportant features to improve the model performance. MLP, on the other hand, is a powerful nonlinear model capable of capturing complex data patterns and relationships through multiple hidden layers [73]. If the feature relationships in the data are very complex and nonlinear, MLP is usually able to learn these complexities through its deep network structure.
2. Feature interaction and nonlinearity
  XGB and RF typically perform well when feature relationships are relatively independent and linearly differentiable, and XGB in particular performs well for classification problems and structured datasets [71]. However, if the relationships between features in a dataset are extremely complex or masked by noise, these models may not be able to capture these relationships effectively. In particular, when gene expression data contain many low or extreme values and are sparsely represented after normalization methods such as Log or Z, these models may struggle even more to capture complex nonlinear patterns.
3. Model robustness and sensitivity to noise
  While XGB and RF are resistant to general outliers and noise, they may be less effective in the face of extreme noise or outlier distributions, especially in cases where decision trees are prone to overfitting on outliers. In contrast, MLP may be better at resisting noise through its nonlinear and multilevel structure during training, especially when equipped with appropriate regularization techniques (e.g., Dropout).
4. Scale sensitivity of different models
  Feature scale sensitivity is the degree to which a ML model is sensitive to changes in the range and scale of input feature values [87]. Different models have different sensitivities to feature scales. Distance-based models, such as LR and SVM, are very sensitive to feature scales, while tree-based models, such as decision trees, random forests, and gradient boosting trees are not sensitive to feature scales, so that the performance of the former improves much more after normalization. As a neural network model, on the other hand, the structure and learning algorithm of MLP enable it to adapt to different data scales, and the appropriate normalization method also helps to speed up the training and avoid some gradient problems, such as gradient vanishing or exploding, which leads to a more stable model performance.

By further analyzing the performance of the LR and MLP models on datasets with different preprocessing, we find that Balanced Accuracy seems to be relatively more stable than Kappa value. From a data perspective, this suggests that preprocessing tools such as normalization, feature selection, and outlier handling change the distribution of the raw data or the relationship between features to a certain extent, thus affecting the way the model learns. The change in data distribution directly affects the decision boundaries of the model, making the model’s classification boundaries significantly different after different preprocessing, thus enhancing Kappa, which specifically emphasizes the consistency between actual and random classification. On the other hand, the relative stability of Balanced Accuracy suggests that, despite the change in the classification boundaries, the model’s ability to recognize the various categories on the whole consistency was maintained. From a modeling perspective, LR and MLP show better robustness when dealing with different data. Even if the preprocessing changes some features of the data, these two models are still able to recognize the categories effectively and maintain a more stable classification performance.

We also recognize that this study has its limitations. The training process was limited by the small number of available samples and did not take into account the effects caused by imbalance. During the analysis process, due to the limited computational power, we were unable to examine the variations in gene selection thresholds, normalization methods in a large and detailed way, especially as we mentioned earlier that further research on suitable gene selection methods is needed.

In addition to the models examined in this study, we acknowledge the growing availability of novel ML approaches that may offer advantages in small-sample, high-dimensional settings. For example, penalized regression methods such as elastic net have shown strong performance in feature selection under high-dimensional conditions, though our preliminary tests did not yield improved accuracy in this setting and introduced additional complexity. More recent methods like TabPFN v2 [88], which leverage pre-trained transformer architectures for tabular data, demonstrate impressive generalization in few-shot tasks and warrant further exploration. Beyond these, self-supervised learning techniques (e.g., contrastive learning) [89,90], meta-learning frameworks (e.g., Prototypical Networks) [91], and attention-based tabular models (e.g., TabTransformer, SAINT) [92,93] may offer alternative strategies that better capture nonlinear patterns or perform robustly with limited labeled data. While not explored in this study, these emerging approaches represent promising future directions for genomic prediction tasks.

However, we hope to use this study as an example to provide researchers with a comprehensive set of classification model construction strategies for various classification prediction studies.

## 5. Conclusions

To improve ML performance in cross-platform testing on independent datasets, this study proposes a strategy based on novel NDEG-based data normalization. It combines gene selection scenarios, normalization methods and classification models. The BRCA data in TCGA were generated using both microarray and RNA-seq platforms for the sample set, and thus was used in this study. Stable NDEG and DEG with variability were first searched for by ANOVA and used for the screening of the corresponding datasets.

In this cross-platform data classification study, RNA-seq provides more comprehensive and precise transcriptome information since the overall performance of model S trained on RNA-seq data is much better than that of model A trained on Microarray data. The results show that NDEG and DEG gene selection can effectively improve the classification performance of ML models. Thus, it is recommended to determine the optimal model based on the model performance matrices obtained from the NDEG and DEG gene threshold changes. The choice of normalization method is crucial for ML classification performance, while the parametric normalization methods are overall inferior to the nonparametric ones. At the same time, different classifiers perform differently on different data, and the normalization methods and classifiers should be considered together.

## Supplementary Material

Supplementary

Supplementary Materials

The additional data and information can be downloaded at: https://media.sciltp.com/articles/others/2505261010239036/SupplementarymaterialV2.zip.

## Figures and Tables

**Table 1.** The results of the statistical analysis of the performance of the different classifiers on each dataset processed with various DEG and different normalization treatments (for Model-A).

Kappa				Balanced Accuracy			

SVM	Mean	Standard Deviation	Coefficient of Variation	SVM	Mean	Standard Deviation	Coefficient of Variation

Raw_data	0.257	0.037	0.143	Raw_data	0.402	0.035	0.088

LOG	0.259	0.038	0.148	LOG	0.402	0.019	0.048
NST	0.249	0.012	0.046	NST	0.397	0.011	0.026
QN	0.257	0.028	0.110	QN	0.411	0.036	0.087
Z	0.263	0.035	0.133	Z	0.407	0.030	0.073
NPN	0.249	0.012	0.047	NPN	0.397	0.008	0.020

RF				RF			

Raw_data	0.149	0.130	0.874	Raw_data	0.283	0.075	0.263
LOG	0.179	0.106	0.595	LOG	0.289	0.055	0.189
NST	0.258	0.113	0.437	NST	0.355	0.061	0.172
QN	0.167	0.151	0.908	QN	0.297	0.096	0.322
Z	0.149	0.101	0.679	Z	0.278	0.060	0.218
NPN	0.181	0.120	0.665	NPN	0.327	0.082	0.250

LR				LR			

Raw_data	0.113	0.115	1.018	Raw_data	0.288	0.088	0.305
LOG	0.131	0.098	0.751	LOG	0.304	0.078	0.255
NST	0.130	0.086	0.665	NST	0.302	0.068	0.224
QN	0.077	0.085	1.095	QN	0.263	0.068	0.261
Z	0.153	0.095	0.621	Z	0.322	0.077	0.238
NPN	0.152	0.084	0.556	NPN	0.319	0.067	0.209

MLP				MLP			

Raw_data	0.158	0.116	0.735	Raw_data	0.336	0.110	0.328
LOG	0.202	0.109	0.540	LOG	0.376	0.107	0.285
NST	0.222	0.083	0.374	NST	0.382	0.077	0.201
QN	0.167	0.096	0.577	QN	0.343	0.091	0.265
Z	0.181	0.077	0.424	Z	0.347	0.065	0.187
NPN	0.182	0.126	0.691	NPN	0.351	0.118	0.336

XGB				XGB			

Raw_data	0.217	0.108	0.497	Raw_data	0.370	0.066	0.178
LOG	0.112	0.105	0.931	LOG	0.272	0.085	0.314
NST	0.228	0.168	0.734	NST	0.393	0.109	0.277
QN	0.119	0.100	0.843	QN	0.297	0.116	0.392
Z	0.148	0.098	0.665	Z	0.321	0.069	0.215
NPN	0.296	0.129	0.436	NPN	0.455	0.095	0.208

DEG, Differentially expressed genes; LR, Logistic Regression; MLP, Multilayer Perceptron; RF, Random Forest; SVM, (Linear) support vector machine; XGB, Extreme Gradient Boosting.

**Table 2.** Some classification performance results on data Selected by NDEG and DEG genes (for Model-S).

Balanced Accuracy																
MLP																
LOG-RQN	0.001	0.002	0.003	0.004	0.005	0.006	0.007	0.008	0.009	0.01	0.02	0.03	0.05	0.07	0.1	1
0.98	0.691	0.698	0.681	0.705	0.685	0.702	0.694	0.687	0.700	0.692	0.679	0.685	0.681	0.771	0.680	0.702
0.95	0.691	0.721	0.706	0.694	0.748	0.695	0.727	0.671	0.696	0.687	0.693	0.694	0.688	0.689	0.707	0.705
0.92	0.694	0.675	0.692	0.686	0.690	0.702	0.730	0.704	0.688	0.684	0.712	0.664	0.678	0.711	0.684	0.706
0.90	0.691	0.712	0.739	0.696	0.697	0.703	0.707	0.673	0.704	0.681	0.688	0.721	0.695	0.699	0.659	0.715
0.85	0.690	0.699	0.691	0.699	0.676	0.689	0.699	0.666	0.715	0.703	0.674	0.694	0.682	0.691	0.694	0.681
SVM																
LOG-RQN-Z	0.001	0.002	0.003	0.004	0.005	0.006	0.007	0.008	0.009	0.01	0.02	0.03	0.05	0.07	0.1	1
0.98	0.738	0.670	0.668	0.700	0.735	0.689	0.702	0.676	0.667	0.660	0.604	0.671	0.656	0.692	0.648	0.695
0.95	0.667	0.660	0.699	0.693	0.718	0.675	0.710	0.680	0.667	0.653	0.657	0.675	0.681	0.685	0.686	0.709
0.92	0.602	0.634	0.659	0.713	0.670	0.693	0.695	0.682	0.680	0.674	0.674	0.597	0.616	0.657	0.663	0.681
0.90	0.652	0.635	0.659	0.718	0.773	0.749	0.675	0.672	0.687	0.601	0.681	0.617	0.560	0.597	0.629	0.689
0.85	0.587	0.584	0.618	0.630	0.645	0.676	0.434	0.504	0.612	0.467	0.431	0.430	0.547	0.544	0.548	0.553
**Kappa**																
MLP																
LOG-RQN	0.001	0.002	0.003	0.004	0.005	0.006	0.007	0.008	0.009	0.01	0.02	0.03	0.05	0.07	0.1	1
0.98	0.834	0.827	0.839	0.852	0.833	0.845	0.831	0.809	0.827	0.845	0.818	0.825	0.835	0.850	0.807	0.838
0.95	0.821	0.863	0.854	0.834	0.841	0.831	0.867	0.799	0.807	0.815	0.837	0.833	0.832	0.830	0.816	0.843
0.92	0.827	0.792	0.837	0.821	0.837	0.832	0.872	0.849	0.821	0.804	0.857	0.796	0.808	0.858	0.823	0.842
0.90	0.822	0.825	0.883	0.840	0.849	0.834	0.815	0.791	0.822	0.803	0.823	0.838	0.835	0.847	0.789	0.856
0.85	0.821	0.844	0.835	0.852	0.826	0.830	0.828	0.786	0.818	0.825	0.813	0.815	0.822	0.851	0.821	0.822
SVM																
LOG-RQN-Z	0.001	0.002	0.003	0.004	0.005	0.006	0.007	0.008	0.009	0.01	0.02	0.03	0.05	0.07	0.1	1
0.98	0.788	0.808	0.801	0.783	0.810	0.811	0.808	0.829	0.778	0.793	0.682	0.787	0.721	0.818	0.785	0.797
0.95	0.737	0.758	0.807	0.797	0.772	0.759	0.818	0.782	0.728	0.745	0.767	0.772	0.739	0.751	0.796	0.820
0.92	0.697	0.739	0.744	0.755	0.751	0.806	0.782	0.762	0.701	0.736	0.716	0.659	0.732	0.751	0.770	0.751
0.90	0.703	0.760	0.712	0.707	0.781	0.783	0.780	0.756	0.787	0.669	0.778	0.680	0.620	0.707	0.705	0.658
0.85	0.690	0.703	0.639	0.706	0.726	0.675	0.579	0.586	0.703	0.541	0.470	0.519	0.688	0.649	0.638	0.547

The color shade indicates the ranking of the metric in the cell among all cells. MLP, Multilayer perceptron; SVM, (Linear) support vector machine.

## Data Availability

The data sets used and/or analyzed of this study are available on the cBioPortal website (https://www.cbioportal.org/, accessed on 25 February 2024). The program coding is available from the corresponding authors on reasonable request.
