# Supplementary material for: Normalization and Selecting Non-Differentially Expressed Genes Improve Machine Learning Modelling of Cross-Platform Transcriptomic Data
Source: Trans Artif Intell. Author manuscript; Available in PMC 2025 Jul 8. (PMC12235674; doi:10.53941/tai.2025.100005)
Supplement: Supplementary [file NIHMS2087281-supplement-Supplementary.zip › Supplementary Figure 1.docx]

Supplementary Figure 1

Microarray Dataset

Dataset with all genes data

Gene selection based on ANOVA

Cleaned Microarray Dataset

Gene matching for labeled samples

Data cleaning

RNA_seq Dataset

Cleaned RNA_seq Dataset

Normalization with LOG, Z, NPN, QN and NST

Dataset screened with varied DEG

Dataset screened with varied DEG + NDEG

Normalization with LOG-NPN-Z, LOG-RQN, LOG-RQN-Z or LOG-NICG-Z

Data cleaning

Matching data based on DEG and NDEG lists

STAGE 1

DEG and NDEG lists

Dataset with all genes data

Dataset screened with varied DEG

Dataset screened with varied DEG + NDEG

Normalized RNA_seq Dataset and Normalized Microarray Dataset

……

……

……

……

Supplementary Figure 1: Stage 1 of the framework of the classification strategy: data cleaning, gene selection and normalization ( Model-A )

Model-A: Microarray Dataset as training set and RNA_seq Dataset as testing set
