# Supplementary material for: Normalization and Selecting Non-Differentially Expressed Genes Improve Machine Learning Modelling of Cross-Platform Transcriptomic Data
Source: Trans Artif Intell. Author manuscript; Available in PMC 2025 Jul 8. (PMC12235674; doi:10.53941/tai.2025.100005)
Supplement: Supplementary [file NIHMS2087281-supplement-Supplementary.zip › Supplementary Figure 2.docx]

Supplementary Figure 2

Normalized Microarray Dataset

75% of the Microarray Dataset using for model training

Sample name list

Data partitioning

Normalized RNA_seq Dataset

Matched samples in RNA_seq Dataset using for testing

Classification prediction on the test set using a trained model

STAGE 2

Classification strategies containing DEG, NDEG selection parameters, normalization method and classification model

Supplementary Figure 2: Stage 2 of the framework of the classification strategy: dataset partitioning, classification model training, prediction and classification performance evaluation ( Model-A )

Model-A: Microarray Dataset as training set and RNA_seq Dataset as testing set

Balanced Accuracy, Kappa, Precision, Recall, F1, AUC

Evaluation performance metrics

25% of the Microarray Dataset

SVM

ML model training with 10-fold CV

RF

LR

MLP

XGB
