# Supplementary material for: Normalization and Selecting Non-Differentially Expressed Genes Improve Machine Learning Modelling of Cross-Platform Transcriptomic Data
Source: Trans Artif Intell. Author manuscript; Available in PMC 2025 Jul 8. (PMC12235674; doi:10.53941/tai.2025.100005)
Supplement: Supplementary [file NIHMS2087281-supplement-Supplementary.zip › Supplementary table 1.docx]

| Supplementary table 1. Classification performance results on data selected by DEG genes and raw-data (Model-S) | | | | | | | | | | | | | | | | | | | | | | | | | | | | | | | | | | | | | | | | | | | | | | | | |  |
| --- | --- | --- | --- | --- | --- | --- | --- | --- | --- | --- | --- | --- | --- | --- | --- | --- | --- | --- | --- | --- | --- | --- | --- | --- | --- | --- | --- | --- | --- | --- | --- | --- | --- | --- | --- | --- | --- | --- | --- | --- | --- | --- | --- | --- | --- | --- | --- | --- | --- |
| Kappa |  | | | **SVM** | | | | | | | | | | | | | | | | | | | | | | | | | | | | | | | | | | | | | | | | | | | | |  |
| gene numbers | 11408 | | | 11790 | | | 12019 | | | 12189 | | | 12303 | | | 12427 | | | 12548 | | | 12621 | | | 12690 | | 12757 | | 13155 | | 13409 | | 13744 | | 13955 | | 14197 | | 15672 | | max | | Mean | Standard Deviation | | | | Coefficient of Variation |  |
| DEG thresholds | 0.001 | | | 0.002 | | | 0.003 | | | 0.004 | | | 0.005 | | | 0.006 | | | 0.007 | | | 0.008 | | | 0.009 | | 0.010 | | 0.020 | | 0.030 | | 0.050 | | 0.070 | | 0.100 | | 1.000 | |  |  |  |  |  |  |  |  |  |
| Raw data | 0.383 | | | 0.181 | | | 0.276 | | | 0.036 | | | 0.181 | | | 0.092 | | | 0.118 | | | 0.247 | | | 0.247 | | 0.108 | | 0.202 | | 0.231 | | 0.119 | | 0.104 | | 0.212 | | 0.395 | | 0.395 | | 0.196 | 0.101 | | | | 0.515 |  |
| LOG | 0.243 | | | 0.279 | | | 0.202 | | | 0.037 | | | 0.133 | | | 0.129 | | | 0.160 | | | 0.103 | | | 0.103 | | 0.176 | | 0.068 | | 0.152 | | 0.131 | | 0.142 | | 0.156 | | 0.325 | | 0.325 | | 0.159 | 0.074 | | | | 0.469 |  |
| NST | 0.512 | | | 0.279 | | | 0.360 | | | 0.248 | | | 0.364 | | | 0.216 | | | 0.286 | | | 0.246 | | | 0.246 | | 0.230 | | 0.231 | | 0.400 | | 0.306 | | 0.466 | | 0.320 | | 0.424 | | 0.512 | | 0.321 | 0.091 | | | | 0.284 |  |
| QN | 0.257 | | | 0.135 | | | 0.257 | | | 0.254 | | | 0.193 | | | 0.172 | | | 0.112 | | | 0.179 | | | 0.179 | | 0.276 | | 0.235 | | 0.305 | | 0.234 | | 0.197 | | 0.246 | | 0.460 | | 0.460 | | 0.231 | 0.080 | | | | 0.348 |  |
| Z | 0.287 | | | 0.294 | | | 0.252 | | | 0.110 | | | 0.139 | | | 0.198 | | | 0.098 | | | 0.195 | | | 0.195 | | 0.262 | | 0.141 | | 0.283 | | 0.082 | | 0.300 | | 0.141 | | 0.384 | | 0.384 | | 0.210 | 0.088 | | | | 0.417 |  |
| NPN | 0.390 | | | 0.377 | | | 0.413 | | | 0.330 | | | 0.340 | | | 0.343 | | | 0.391 | | | 0.282 | | | 0.282 | | 0.332 | | 0.361 | | 0.316 | | 0.369 | | 0.496 | | 0.493 | | 0.436 | | 0.496 | | 0.372 | 0.064 | | | | 0.172 |  |
| Balanced Accuracy | | | | **SVM** | | | | | | | | | | | | | | | | | | | | | | | | | | | | | | | | | | | | | | | | | | | | |  |
| gene numbers | 11408 | | | 11790 | | | 12019 | | | 12189 | | | 12303 | | | 12427 | | | 12548 | | | 12621 | | | 12690 | | 12757 | | 13155 | | 13409 | | 13744 | | 13955 | | 14197 | | 15672 | | max | | Mean | Standard Deviation | | | | Coefficient of Variation |  |
| DEG thresholds | 0.001 | | | 0.002 | | | 0.003 | | | 0.004 | | | 0.005 | | | 0.006 | | | 0.007 | | | 0.008 | | | 0.009 | | 0.010 | | 0.020 | | 0.030 | | 0.050 | | 0.070 | | 0.100 | | 1.000 | |  |  |  |  |  |  |  |  |  |
| Raw data | 0.449 | | | 0.362 | | | 0.384 | | | 0.265 | | | 0.439 | | | 0.223 | | | 0.334 | | | 0.504 | | | 0.504 | | 0.352 | | 0.343 | | 0.375 | | 0.345 | | 0.307 | | 0.380 | | 0.477 | | 0.504 | | 0.378 | 0.080 | | | | 0.213 |  |
| LOG | 0.382 | | | 0.375 | | | 0.438 | | | 0.245 | | | 0.401 | | | 0.296 | | | 0.309 | | | 0.332 | | | 0.332 | | 0.310 | | 0.356 | | 0.300 | | 0.346 | | 0.322 | | 0.310 | | 0.443 | | 0.443 | | 0.344 | 0.054 | | | | 0.156 |  |
| NST | 0.591 | | | 0.442 | | | 0.512 | | | 0.452 | | | 0.573 | | | 0.392 | | | 0.444 | | | 0.526 | | | 0.526 | | 0.485 | | 0.438 | | 0.578 | | 0.541 | | 0.523 | | 0.466 | | 0.596 | | 0.596  0.644 | | 0.505  0.486 | 0.062  0.065 | | | | 0.123  0.134 |  |
| QN | 0.489 | | | 0.551 | | | 0.512 | | | 0.532 | | | 0.465 | | | 0.419 | | | 0.381 | | | 0.458 | | | 0.458 | | 0.493 | | 0.496 | | 0.547 | | 0.498 | | 0.397 | | 0.439 | | 0.644 | |  |  |  |  |  |  |  |  |  |
| Z | 0.384 | | | 0.390 | | | 0.371 | | | 0.307 | | | 0.382 | | | 0.314 | | | 0.256 | | | 0.354 | | | 0.354 | | 0.368 | | 0.375 | | 0.347 | | 0.334 | | 0.428 | | 0.316 | | 0.457 | | 0.457 | | 0.359 | 0.048 | | | | 0.135 |  |
| NPN | 0.517 | | | 0.510 | | | 0.523 | | | 0.525 | | | 0.550 | | | 0.539 | | | 0.481 | | | 0.520 | | | 0.520 | | 0.548 | | 0.465 | | 0.468 | | 0.527 | | 0.556 | | 0.564 | | 0.609 | | 0.609 | | 0.526 | 0.037 | | | | 0.070 |  |
|  |  | | |  | | |  | | |  | | |  | | |  | | |  | | |  | | |  | |  | |  | |  | |  | |  | |  | |  | |  | |  |  | | | |  |  |
| Kappa |  | | | **RF** | | | | | | | | | | | | | | | | | | | | | | | | | | | | | | | | | | | | | | | | | | | | |  |
| gene numbers | 11408 | | | 11790 | | | 12019 | | | 12189 | | | 12303 | | | 12427 | | | 12548 | | | 12621 | | | 12690 | | 12757 | | 13155 | | 13409 | | 13744 | | 13955 | | 14197 | | 15672 | | max | | Mean | Standard Deviation | | | | Coefficient of Variation |  |
| DEG thresholds | 0.001 | | | 0.002 | | | 0.003 | | | 0.004 | | | 0.005 | | | 0.006 | | | 0.007 | | | 0.008 | | | 0.009 | | 0.010 | | 0.020 | | 0.030 | | 0.050 | | 0.070 | | 0.100 | | 1.000 | |  |  |  |  |  |  |  |  |  |
| Raw data | 0.259 | | | 0.408 | | | 0.363 | | | 0.184 | | | 0.274 | | | 0.210 | | | 0.255 | | | 0.290 | | | 0.290 | | 0.299 | | 0.186 | | 0.061 | | 0.124 | | 0.281 | | 0.260 | | 0.287 | | 0.408 | | 0.252 | 0.085 | | | | 0.337 |  |
| LOG | 0.161 | | | 0.243 | | | 0.147 | | | 0.183 | | | 0.066 | | | 0.285 | | | 0.344 | | | 0.113 | | | 0.113 | | 0.102 | | 0.078 | | 0.161 | | 0.040 | | 0.205 | | 0.306 | | 0.121 | | 0.344 | | 0.167 | 0.089 | | | | 0.535 |  |
| NST | 0.247 | | | 0.324 | | | 0.207 | | | 0.164 | | | 0.293 | | | 0.241 | | | 0.072 | | | 0.236 | | | 0.236 | | 0.142 | | 0.040 | | 0.206 | | 0.189 | | 0.404 | | 0.283 | | 0.231 | | 0.404 | | 0.220 | 0.090 | | | | 0.408 |  |
| QN | 0.175 | | | 0.198 | | | 0.000 | | | 0.149 | | | 0.209 | | | 0.106 | | | 0.151 | | | 0.178 | | | 0.178 | | 0.236 | | 0.277 | | 0.103 | | 0.289 | | 0.093 | | 0.162 | | 0.264 | | 0.289 | | 0.173 | 0.075 | | | | 0.436 |  |
| Z | 0.206 | | | 0.108 | | | 0.207 | | | 0.167 | | | 0.128 | | | 0.150 | | | 0.029 | | | 0.035 | | | 0.035 | | 0.197 | | 0.048 | | 0.263 | | 0.160 | | 0.299 | | 0.225 | | 0.324 | | 0.324 | | 0.161 | 0.094 | | | | 0.580 |  |
| NPN | 0.183 | | | 0.206 | | | 0.135 | | | 0.075 | | | 0.117 | | | 0.271 | | | 0.314 | | | 0.302 | | | 0.302 | | 0.340 | | 0.325 | | 0.174 | | 0.198 | | 0.319 | | 0.354 | | 0.266 | | 0.354 | | 0.243 | 0.088 | | | | 0.361 |  |
| Balanced Accuracy | | | | **RF** | | | | | | | | | | | | | | | | | | | | | | | | | | | | | | | | | | | | | | | | | | | | |  |
| gene numbers | 11408 | | | 11790 | | | 12019 | | | 12189 | | | 12303 | | | 12427 | | | 12548 | | | 12621 | | | 12690 | | 12757 | | 13155 | | 13409 | | 13744 | | 13955 | | 14197 | | 15672 | | max | | Mean | Standard Deviation | | | | Coefficient of Variation |  |
| DEG thresholds | 0.001 | | | 0.002 | | | 0.003 | | | 0.004 | | | 0.005 | | | 0.006 | | | 0.007 | | | 0.008 | | | 0.009 | | 0.010 | | 0.020 | | 0.030 | | 0.050 | | 0.070 | | 0.100 | | 1.000 | |  |  |  |  |  |  |  |  |  |
| Raw data | 0.354 | | | 0.438 | | | 0.394 | | | 0.268 | | | 0.359 | | | 0.319 | | | 0.329 | | | 0.351 | | | 0.351 | | 0.369 | | 0.320 | | 0.229 | | 0.263 | | 0.340 | | 0.336 | | 0.379 | | 0.438 | | 0.337 | 0.052 | | | | 0.153 |  |
| LOG | 0.285 | | | 0.333 | | | 0.272 | | | 0.295 | | | 0.236 | | | 0.346 | | | 0.380 | | | 0.254 | | | 0.254 | | 0.251 | | 0.239 | | 0.276 | | 0.219 | | 0.315 | | 0.343 | | 0.253 | | 0.380 | | 0.285 | 0.047 | | | | 0.164 |  |
| NST | 0.325 | | | 0.374 | | | 0.306 | | | 0.284 | | | 0.355 | | | 0.312 | | | 0.232 | | | 0.323 | | | 0.323 | | 0.269 | | 0.226 | | 0.297 | | 0.316 | | 0.477 | | 0.357 | | 0.32 | | 0.477  0.355 | | 0.319  0.291 | 0.059  0.042 | | | | 0.184  0.145 |  |
| QN | 0.289 | | | 0.303 | | | 0.200 | | | 0.281 | | | 0.305 | | | 0.256 | | | 0.277 | | | 0.296 | | | 0.296 | | 0.322 | | 0.355 | | 0.252 | | 0.354 | | 0.245 | | 0.276 | | 0.353 | |  |  |  |  |  |  |  |  |  |
| Z | 0.298 | | | 0.249 | | | 0.317 | | | 0.282 | | | 0.289 | | | 0.275 | | | 0.214 | | | 0.219 | | | 0.219 | | 0.284 | | 0.222 | | 0.337 | | 0.282 | | 0.405 | | 0.319 | | 0.379 | | 0.405 | | 0.287 | 0.056 | | | | 0.196 |  |
| NPN | 0.305 | | | 0.310 | | | 0.256 | | | 0.234 | | | 0.260 | | | 0.342 | | | 0.355 | | | 0.336 | | | 0.336 | | 0.381 | | 0.391 | | 0.289 | | 0.288 | | 0.365 | | 0.383 | | 0.347 | | 0.391 | | 0.324 | 0.048 | | | | 0.149 |  |
|  | | |  | | |  | | |  | | |  | | |  | | |  | | |  | | |  | |  | |  | |  | |  | |  | |  | |  | |  | |  | |  |  | |  | | |
| Kappa |  | | | **LR** | | | | | | | | | | | | | | | | | | | | | | | | | | | | | | | | | | | | | | | | | | | | |  |
| gene numbers | 11408 | | | 11790 | | | 12019 | | | 12189 | | | 12303 | | | 12427 | | | 12548 | | | 12621 | | | 12690 | | 12757 | | 13155 | | 13409 | | 13744 | | 13955 | | 14197 | | 15672 | | max | | Mean | Standard Deviation | | | | Coefficient of Variation |  |
| DEG thresholds | 0.001 | | | 0.002 | | | 0.003 | | | 0.004 | | | 0.005 | | | 0.006 | | | 0.007 | | | 0.008 | | | 0.009 | | 0.010 | | 0.020 | | 0.030 | | 0.050 | | 0.070 | | 0.100 | | 1.000 | |  |  |  |  |  |  |  |  |  |
| Raw data | 0.316 | | | 0.356 | | | 0.188 | | | 0.380 | | | 0.447 | | | 0.391 | | | 0.408 | | | 0.217 | | | 0.217 | | 0.431 | | 0.280 | | 0.428 | | 0.352 | | 0.236 | | 0.290 | | 0.317 | | 0.447 | | 0.328 | 0.084 | | | | 0.256 |  |
| LOG | 0.344 | | | 0.329 | | | 0.322 | | | 0.419 | | | 0.324 | | | 0.318 | | | 0.377 | | | 0.383 | | | 0.383 | | 0.458 | | 0.415 | | 0.380 | | 0.400 | | 0.460 | | 0.272 | | 0.359 | | 0.460 | | 0.371 | 0.052 | | | | 0.140 |  |
| NST | 0.584 | | | 0.398 | | | 0.329 | | | 0.428 | | | 0.415 | | | 0.500 | | | 0.418 | | | 0.335 | | | 0.335 | | 0.499 | | 0.439 | | 0.547 | | 0.432 | | 0.419 | | 0.555 | | 0.476 | | 0.584 | | 0.444 | 0.078 | | | | 0.176 |  |
| QN | 0.509 | | | 0.476 | | | 0.429 | | | 0.415 | | | 0.482 | | | 0.410 | | | 0.388 | | | 0.427 | | | 0.427 | | 0.470 | | 0.415 | | 0.492 | | 0.507 | | 0.426 | | 0.399 | | 0.504 | | 0.509 | | 0.448 | 0.042 | | | | 0.093 |  |
| Z | 0.328 | | | 0.286 | | | 0.225 | | | 0.420 | | | 0.298 | | | 0.211 | | | 0.344 | | | 0.413 | | | 0.413 | | 0.376 | | 0.283 | | 0.382 | | 0.316 | | 0.396 | | 0.114 | | 0.385 | | 0.420 | | 0.324 | 0.086 | | | | 0.265 |  |
| NPN | 0.383 | | | 0.457 | | | 0.522 | | | 0.466 | | | 0.538 | | | 0.451 | | | 0.386 | | | 0.348 | | | 0.348 | | 0.462 | | 0.407 | | 0.457 | | 0.452 | | 0.363 | | 0.523 | | 0.468 | | 0.538 | | 0.439 | 0.061 | | | | 0.139 |  |
| Balanced Accuracy | | | | **LR** | | | | | | | | | | | | | | | | | | | | | | | | | | | | | | | | | | | | | | | | | | | | |  |
| gene numbers | 11408 | | | 11790 | | | 12019 | | | 12189 | | | 12303 | | | 12427 | | | 12548 | | | 12621 | | | 12690 | | 12757 | | 13155 | | 13409 | | 13744 | | 13955 | | 14197 | | 15672 | | max | | Mean | Standard Deviation | | | | Coefficient of Variation |  |
| DEG thresholds | 0.001 | | | 0.002 | | | 0.003 | | | 0.004 | | | 0.005 | | | 0.006 | | | 0.007 | | | 0.008 | | | 0.009 | | 0.010 | | 0.020 | | 0.030 | | 0.050 | | 0.070 | | 0.100 | | 1.000 | |  |  |  |  |  |  |  |  |  |
| Raw data | 0.426 | | | 0.409 | | | 0.317 | | | 0.452 | | | 0.553 | | | 0.475 | | | 0.473 | | | 0.325 | | | 0.325 | | 0.501 | | 0.336 | | 0.590 | | 0.469 | | 0.313 | | 0.384 | | 0.414 | | 0.590 | | 0.423 | 0.086 | | | | 0.203 |  |
| LOG | 0.429 | | | 0.460 | | | 0.466 | | | 0.451 | | | 0.468 | | | 0.443 | | | 0.434 | | | 0.512 | | | 0.512 | | 0.479 | | 0.528 | | 0.475 | | 0.444 | | 0.519 | | 0.353 | | 0.506 | | 0.528 | | 0.467 | 0.044 | | | | 0.094 |  |
| NST | 0.559 | | | 0.438 | | | 0.389 | | | 0.488 | | | 0.492 | | | 0.53 | | | 0.524 | | | 0.397 | | | 0.397 | | 0.609 | | 0.503 | | 0.644 | | 0.444 | | 0.443 | | 0.589 | | 0.589 | | 0.644  0.589 | | 0.502  0.507 | 0.080  0.054 | | | | 0.160  0.106 |  |
| QN | 0.582 | | | 0.542 | | | 0.480 | | | 0.450 | | | 0.540 | | | 0.490 | | | 0.424 | | | 0.502 | | | 0.502 | | 0.487 | | 0.492 | | 0.564 | | 0.589 | | 0.492 | | 0.411 | | 0.573 | |  |  |  |  |  |  |  |  |  |
| Z | 0.427 | | | 0.378 | | | 0.322 | | | 0.490 | | | 0.355 | | | 0.320 | | | 0.368 | | | 0.435 | | | 0.435 | | 0.422 | | 0.419 | | 0.444 | | 0.406 | | 0.485 | | 0.203 | | 0.468 | | 0.490 | | 0.399 | 0.073 | | | | 0.184 |  |
| NPN | 0.429 | | | 0.474 | | | 0.489 | | | 0.533 | | | 0.608 | | | 0.515 | | | 0.412 | | | 0.390 | | | 0.390 | | 0.525 | | 0.438 | | 0.478 | | 0.509 | | 0.405 | | 0.533 | | 0.523 | | 0.608 | | 0.478 | 0.063 | | | | 0.131 |  |
|  | | |  | | |  | | |  | | |  | | |  | | |  | | |  | | |  | |  | |  | |  | |  | |  | |  | |  | |  | |  | |  |  | |  | | |
| Kappa |  | | | **MLP** | | | | | | | | | | | | | | | | | | | | | | | | | | | | | | | | | | | | | | | | | | | | |  |
| gene numbers | 11408 | | | 11790 | | | 12019 | | | 12189 | | | 12303 | | | 12427 | | | 12548 | | | 12621 | | | 12690 | | 12757 | | 13155 | | 13409 | | 13744 | | 13955 | | 14197 | | 15672 | | max | | Mean | Standard Deviation | | | | Coefficient of Variation |  |
| DEG thresholds | 0.001 | | | 0.002 | | | 0.003 | | | 0.004 | | | 0.005 | | | 0.006 | | | 0.007 | | | 0.008 | | | 0.009 | | 0.010 | | 0.020 | | 0.030 | | 0.050 | | 0.070 | | 0.100 | | 1.000 | |  |  |  |  |  |  |  |  |  |
| Raw data | 0.440 | | | 0.226 | | | 0.420 | | | 0.270 | | | 0.348 | | | 0.327 | | | 0.190 | | | 0.120 | | | 0.120 | | 0.424 | | 0.279 | | 0.504 | | 0.284 | | 0.310 | | 0.316 | | 0.357 | | 0.504 | | 0.308 | 0.110 | | | | 0.356 |  |
| LOG | 0.449 | | | 0.240 | | | 0.430 | | | 0.137 | | | 0.307 | | | 0.457 | | | 0.245 | | | 0.426 | | | 0.426 | | 0.228 | | 0.351 | | 0.260 | | 0.281 | | 0.255 | | 0.383 | | 0.393 | | 0.457 | | 0.329 | 0.097 | | | | 0.296 |  |
| NST | 0.399 | | | 0.385 | | | 0.305 | | | 0.350 | | | 0.441 | | | 0.246 | | | 0.335 | | | 0.315 | | | 0.315 | | 0.400 | | 0.311 | | 0.323 | | 0.074 | | 0.305 | | 0.375 | | 0.444 | | 0.444 | | 0.333 | 0.088 | | | | 0.263 |  |
| QN | 0.433 | | | 0.340 | | | 0.376 | | | 0.329 | | | 0.394 | | | 0.348 | | | 0.354 | | | 0.413 | | | 0.413 | | 0.437 | | 0.441 | | 0.295 | | 0.414 | | 0.405 | | 0.373 | | 0.502 | | 0.502 | | 0.392 | 0.051 | | | | 0.131 |  |
| Z | 0.407 | | | 0.416 | | | 0.271 | | | 0.337 | | | 0.275 | | | 0.347 | | | 0.316 | | | 0.459 | | | 0.459 | | 0.336 | | 0.326 | | 0.466 | | 0.360 | | 0.179 | | 0.419 | | 0.319 | | 0.466 | | 0.356 | 0.079 | | | | 0.221 |  |
| NPN | 0.313 | | | 0.374 | | | 0.412 | | | 0.418 | | | 0.323 | | | 0.379 | | | 0.401 | | | 0.216 | | | 0.216 | | 0.118 | | 0.349 | | 0.345 | | 0.253 | | 0.318 | | 0.300 | | 0.529 | | 0.529 | | 0.329 | 0.097 | | | | 0.296 |  |
| Balanced Accuracy | | | | **MLP** | | | | | | | | | | | | | | | | | | | | | | | | | | | | | | | | | | | | | | | | | | | | |  |
| gene numbers | 11408 | | | 11790 | | | 12019 | | | 12189 | | | 12303 | | | 12427 | | | 12548 | | | 12621 | | | 12690 | | 12757 | | 13155 | | 13409 | | 13744 | | 13955 | | 14197 | | 15672 | | max | | Mean | Standard Deviation | | | | Coefficient of Variation |  |
| DEG thresholds | 0.001 | | | 0.002 | | | 0.003 | | | 0.004 | | | 0.005 | | | 0.006 | | | 0.007 | | | 0.008 | | | 0.009 | | 0.010 | | 0.020 | | 0.030 | | 0.050 | | 0.070 | | 0.100 | | 1.000 | |  |  |  |  |  |  |  |  |  |
| Raw data | 0.513 | | | 0.393 | | | 0.493 | | | 0.426 | | | 0.546 | | | 0.538 | | | 0.343 | | | 0.376 | | | 0.376 | | 0.457 | | 0.353 | | 0.503 | | 0.347 | | 0.389 | | 0.475 | | 0.465 | | 0.546 | | 0.437 | 0.070 | | | | 0.160 |  |
| LOG | 0.607 | | | 0.409 | | | 0.574 | | | 0.390 | | | 0.395 | | | 0.482 | | | 0.384 | | | 0.440 | | | 0.440 | | 0.357 | | 0.493 | | 0.417 | | 0.375 | | 0.321 | | 0.451 | | 0.502 | | 0.607 | | 0.440 | 0.077 | | | | 0.175 |  |
| NST | 0.482 | | | 0.417 | | | 0.408 | | | 0.396 | | | 0.505 | | | 0.349 | | | 0.449 | | | 0.372 | | | 0.372 | | 0.415 | | 0.421 | | 0.361 | | 0.351 | | 0.425 | | 0.415 | | 0.462 | | 0.505  0.582 | | 0.413  0.469 | 0.046  0.051 | | | | 0.111  0.109 |  |
| QN | 0.455 | | | 0.486 | | | 0.444 | | | 0.406 | | | 0.470 | | | 0.417 | | | 0.406 | | | 0.446 | | | 0.446 | | 0.504 | | 0.567 | | 0.446 | | 0.492 | | 0.438 | | 0.494 | | 0.582 | |  |  |  |  |  |  |  |  |  |
| Z | 0.431 | | | 0.412 | | | 0.401 | | | 0.477 | | | 0.513 | | | 0.383 | | | 0.339 | | | 0.486 | | | 0.486 | | 0.468 | | 0.377 | | 0.583 | | 0.412 | | 0.348 | | 0.484 | | 0.417 | | 0.583 | | 0.439 | 0.065 | | | | 0.148 |  |
| NPN | 0.466 | | | 0.418 | | | 0.515 | | | 0.431 | | | 0.420 | | | 0.428 | | | 0.414 | | | 0.391 | | | 0.391 | | 0.327 | | 0.378 | | 0.407 | | 0.337 | | 0.354 | | 0.332 | | 0.604 | | 0.604 | | 0.413 | 0.071 | | | | 0.172 |  |
|  | | | |  | | |  | | |  | | |  | | |  | | |  | | |  | | |  | |  | |  | |  | |  | |  | |  | |  | |  | |  |  | |  | |  | |
| Kappa |  | | | **XGB** | | | | | | | | | | | | | | | | | | | | | | | | | | | | | | | | | | | | | | | | | | | | |  |
| gene numbers | 11408 | | | 11790 | | | 12019 | | | 12189 | | | 12303 | | | 12427 | | | 12548 | | | 12621 | | | 12690 | | 12757 | | 13155 | | 13409 | | 13744 | | 13955 | | 14197 | | 15672 | | max | | Mean | Standard Deviation | | | | Coefficient of Variation |  |
| DEG thresholds | 0.001 | | | 0.002 | | | 0.003 | | | 0.004 | | | 0.005 | | | 0.006 | | | 0.007 | | | 0.008 | | | 0.009 | | 0.010 | | 0.020 | | 0.030 | | 0.050 | | 0.070 | | 0.100 | | 1.000 | |  |  |  |  |  |  |  |  |  |
| Raw data | 0.365 | | | 0.415 | | | 0.310 | | | 0.247 | | | 0.174 | | | 0.170 | | | 0.393 | | | 0.253 | | | 0.253 | | 0.375 | | 0.289 | | 0.166 | | 0.323 | | 0.184 | | 0.283 | | 0.372 | | 0.415 | | 0.286 | 0.084 | | | | 0.294 |  |
| LOG | 0.281 | | | 0.467 | | | 0.238 | | | 0.341 | | | 0.293 | | | 0.170 | | | 0.477 | | | 0.311 | | | 0.311 | | 0.390 | | 0.287 | | 0.205 | | 0.476 | | 0.413 | | 0.389 | | 0.265 | | 0.477 | | 0.332 | 0.095 | | | | 0.287 |  |
| NST | 0.337 | | | 0.399 | | | 0.455 | | | 0.332 | | | 0.373 | | | 0.483 | | | 0.469 | | | 0.433 | | | 0.433 | | 0.436 | | 0.233 | | 0.455 | | 0.336 | | 0.420 | | 0.459 | | 0.307 | | 0.483 | | 0.397 | 0.071 | | | | 0.178 |  |
| QN | 0.263 | | | 0.215 | | | 0.377 | | | 0.216 | | | 0.273 | | | 0.107 | | | 0.190 | | | 0.252 | | | 0.252 | | 0.370 | | 0.308 | | 0.256 | | 0.164 | | 0.324 | | 0.209 | | 0.342 | | 0.377 | | 0.257 | 0.075 | | | | 0.290 |  |
| Z | 0.272 | | | 0.352 | | | 0.255 | | | 0.244 | | | 0.421 | | | 0.340 | | | 0.397 | | | 0.286 | | | 0.286 | | 0.326 | | 0.332 | | 0.251 | | 0.321 | | 0.486 | | 0.223 | | 0.230 | | 0.486 | | 0.314 | 0.074 | | | | 0.235 |  |
| NPN | 0.350 | | | 0.417 | | | 0.315 | | | 0.473 | | | 0.373 | | | 0.433 | | | 0.459 | | | 0.320 | | | 0.320 | | 0.411 | | 0.351 | | 0.211 | | 0.431 | | 0.401 | | 0.404 | | 0.347 | | 0.473 | | 0.376 | 0.067 | | | | 0.177 |  |
| Balanced Accuracy | | | | **XGB** | | | | | | | | | | | | | | | | | | | | | | | | | | | | | | | | | | | | | | | | | | | | |  |
| gene numbers | 11408 | | | 11790 | | | 12019 | | | 12189 | | | 12303 | | | 12427 | | | 12548 | | | 12621 | | | 12690 | | 12757 | | 13155 | | 13409 | | 13744 | | 13955 | | 14197 | | 15672 | | max | | Mean | Standard Deviation | | | | Coefficient of Variation |  |
| DEG thresholds | 0.001 | | | 0.002 | | | 0.003 | | | 0.004 | | | 0.005 | | | 0.006 | | | 0.007 | | | 0.008 | | | 0.009 | | 0.010 | | 0.020 | | 0.030 | | 0.050 | | 0.070 | | 0.100 | | 1.000 | |  |  |  |  |  |  |  |  |  |
| Raw data | 0.485 | | | 0.469 | | | 0.444 | | | 0.388 | | | 0.340 | | | 0.274 | | | 0.447 | | | 0.449 | | | 0.449 | | 0.527 | | 0.370 | | 0.356 | | 0.448 | | 0.348 | | 0.373 | | 0.496 | | 0.527 | | 0.416 | 0.068 | | | | 0.164 |  |
| LOG | 0.426 | | | 0.486 | | | 0.342 | | | 0.468 | | | 0.385 | | | 0.291 | | | 0.503 | | | 0.399 | | | 0.399 | | 0.516 | | 0.363 | | 0.302 | | 0.516 | | 0.442 | | 0.423 | | 0.452 | | 0.516 | | 0.420 | 0.071 | | | | 0.169 |  |
| NST | 0.386 | | | 0.461 | | | 0.446 | | | 0.4 | | | 0.46 | | | 0.487 | | | 0.496 | | | 0.472 | | | 0.472 | | 0.452 | | 0.296 | | 0.496 | | 0.446 | | 0.453 | | 0.497 | | 0.385 | | 0.497  0.476 | | 0.444  0.388 | 0.054 | | | | 0.121  0.113 |  |
| QN | 0.365 | | | 0.418 | | | 0.426 | | | 0.369 | | | 0.392 | | | 0.366 | | | 0.334 | | | 0.357 | | | 0.357 | | 0.422 | | 0.456 | | 0.332 | | 0.396 | | 0.476 | | 0.333 | | 0.415 | |  |  |  | 0.044 | | | |  |  |
| Z | 0.394 | | | 0.411 | | | 0.373 | | | 0.382 | | | 0.473 | | | 0.396 | | | 0.467 | | | 0.376 | | | 0.376 | | 0.389 | | 0.369 | | 0.341 | | 0.431 | | 0.496 | | 0.313 | | 0.445 | | 0.496 | | 0.402 | 0.049 | | | | 0.123 |  |
| NPN | 0.412 | | | 0.489 | | | 0.379 | | | 0.480 | | | 0.505 | | | 0.458 | | | 0.488 | | | 0.408 | | | 0.408 | | 0.443 | | 0.416 | | 0.339 | | 0.473 | | 0.449 | | 0.457 | | 0.396 | | 0.505 | | 0.438 | 0.046 | | | | 0.105 |  |
