# Supplementary material for: Normalization and Selecting Non-Differentially Expressed Genes Improve Machine Learning Modelling of Cross-Platform Transcriptomic Data
Source: Trans Artif Intell. Author manuscript; Available in PMC 2025 Jul 8. (PMC12235674; doi:10.53941/tai.2025.100005)
Supplement: Supplementary [file NIHMS2087281-supplement-Supplementary.zip › Supplementary table 2.docx]

| Supplementary table 2. Classification performance results on data selected by DEG genes and raw-data (Model-A) | | | | | | | | | | | | | | | | | | | | | | | | | | | | | | | | | | | | | | | | | |
| --- | --- | --- | --- | --- | --- | --- | --- | --- | --- | --- | --- | --- | --- | --- | --- | --- | --- | --- | --- | --- | --- | --- | --- | --- | --- | --- | --- | --- | --- | --- | --- | --- | --- | --- | --- | --- | --- | --- | --- | --- | --- |
| Kappa | | |  | | **SVM** | | | | | | | | | | | | | | | | | | | | | | | | | | | | | | | | | | | | |
| gene numbers | | | 11408 | | 11790 | | 12019 | | 12189 | | 12303 | | 12427 | | 12548 | | 12621 | | 12690 | | 12757 | | 13155 | | 13409 | | 13744 | | 13955 | | 14197 | | 15672 | | max | | Mean | | Standard Deviation | Coefficient of Variation | |
| DEG thresholds | | | 0.001 | | 0.002 | | 0.003 | | 0.004 | | 0.005 | | 0.006 | | 0.007 | | 0.008 | | 0.009 | | 0.010 | | 0.020 | | 0.030 | | 0.050 | | 0.070 | | 0.100 | | 1.000 | |  |  |  |  |  |  |  |
| Raw data | | | 0.266 | | 0.253 | | 0.270 | | 0.248 | | 0.248 | | 0.219 | | 0.256 | | 0.251 | | 0.241 | | 0.241 | | 0.232 | | 0.385 | | 0.244 | | 0.235 | | 0.257 | | 0.268 | | 0.385 | | 0.257 | | 0.037 | 0.143 | |
| LOG | | | 0.395 | | 0.241 | | 0.243 | | 0.260 | | 0.266 | | 0.217 | | 0.259 | | 0.251 | | 0.255 | | 0.245 | | 0.246 | | 0.242 | | 0.244 | | 0.266 | | 0.250 | | 0.262 | | 0.395 | | 0.259 | | 0.038 | 0.148 | |
| NST | | | 0.258 | | 0.241 | | 0.248 | | 0.251 | | 0.259 | | 0.221 | | 0.255 | | 0.273 | | 0.249 | | 0.240 | | 0.234 | | 0.254 | | 0.255 | | 0.247 | | 0.247 | | 0.253 | | 0.273 | | 0.249 | | 0.012 | 0.046 | |
| QN | | | 0.250 | | 0.250 | | 0.328 | | 0.292 | | 0.267 | | 0.202 | | 0.263 | | 0.258 | | 0.237 | | 0.225 | | 0.242 | | 0.268 | | 0.243 | | 0.282 | | 0.255 | | 0.253 | | 0.328 | | 0.257 | | 0.028 | 0.110 | |
| Z | | | 0.265 | | 0.250 | | 0.256 | | 0.272 | | 0.338 | | 0.224 | | 0.259 | | 0.246 | | 0.236 | | 0.249 | | 0.241 | | 0.356 | | 0.258 | | 0.264 | | 0.257 | | 0.240 | | 0.356 | | 0.263 | | 0.035 | 0.133 | |
| NPN | | | 0.269 | | 0.235 | | 0.247 | | 0.234 | | 0.254 | | 0.242 | | 0.256 | | 0.272 | | 0.250 | | 0.243 | | 0.253 | | 0.239 | | 0.233 | | 0.248 | | 0.249 | | 0.262 | | 0.272 | | 0.249 | | 0.012 | 0.047 | |
| Balanced Accuracy | | | | | **SVM** | | | | | | | | | | | | | | | | | | | | | | | | | | | | | | | | | | | | |
| gene numbers | | | 11408 | | 11790 | | 12019 | | 12189 | | 12303 | | 12427 | | 12548 | | 12621 | | 12690 | | 12757 | | 13155 | | 13409 | | 13744 | | 13955 | | 14197 | | 15672 | | max | | Mean | | Standard Deviation | Coefficient of Variation | |
| DEG thresholds | | | 0.001 | | 0.002 | | 0.003 | | 0.004 | | 0.005 | | 0.006 | | 0.007 | | 0.008 | | 0.009 | | 0.010 | | 0.020 | | 0.030 | | 0.050 | | 0.070 | | 0.100 | | 1.000 | |  |  |  |  |  |  |  |
| Raw data | | | 0.400 | | 0.391 | | 0.407 | | 0.395 | | 0.400 | | 0.372 | | 0.400 | | 0.400 | | 0.391 | | 0.385 | | 0.390 | | 0.532 | | 0.393 | | 0.384 | | 0.400 | | 0.400 | | 0.532 | | 0.402 | | 0.035 | 0.088 | |
| LOG | | | 0.455 | | 0.391 | | 0.391 | | 0.398 | | 0.404 | | 0.369 | | 0.400 | | 0.400 | | 0.391 | | 0.391 | | 0.391 | | 0.399 | | 0.426 | | 0.425 | | 0.400 | | 0.400 | | 0.455 | | 0.402 | | 0.019 | 0.048 | |
| NST | | | 0.400 | | 0.391 | | 0.394 | | 0.400 | | 0.400 | | 0.376 | | 0.400 | | 0.423 | | 0.391 | | 0.391 | | 0.397 | | 0.413 | | 0.393 | | 0.390 | | 0.400 | | 0.400 | | 0.423 | | 0.397 | | 0.011 | 0.026 | |
| QN | | | 0.433 | | 0.391 | | 0.509 | | 0.457 | | 0.410 | | 0.359 | | 0.400 | | 0.418 | | 0.385 | | 0.379 | | 0.391 | | 0.425 | | 0.391 | | 0.434 | | 0.400 | | 0.391 | | 0.509 | | 0.411 | | 0.036 | 0.087 | |
| Z | | | 0.404 | | 0.403 | | 0.394 | | 0.402 | | 0.432 | | 0.375 | | 0.400 | | 0.400 | | 0.390 | | 0.398 | | 0.391 | | 0.509 | | 0.401 | | 0.406 | | 0.400 | | 0.400 | | 0.509 | | 0.407 | | 0.030 | 0.073 | |
| NPN | | | 0.400 | | 0.390 | | 0.404 | | 0.395 | | 0.400 | | 0.387 | | 0.400 | | 0.410 | | 0.391 | | 0.391 | | 0.397 | | 0.396 | | 0.384 | | 0.413 | | 0.400 | | 0.400 | | 0.413 | | 0.397 | | 0.008 | 0.020 | |
|  |  | |  | |  | |  | |  | |  | |  | |  | |  | |  | |  | |  | |  | |  | |  | |  | |  | |  | |  | | |  |  |
| Kappa | | |  | | **RF** | | | | | | | | | | | | | | | | | | | | | | | | | | | | | | | | | | | | |
| gene numbers | | | 11408 | | 11790 | | 12019 | | 12189 | | 12303 | | 12427 | | 12548 | | 12621 | | 12690 | | 12757 | | 13155 | | 13409 | | 13744 | | 13955 | | 14197 | | 15672 | | max | | Mean | | Standard Deviation | Coefficient of Variation | |
| DEG thresholds | | | 0.001 | | 0.002 | | 0.003 | | 0.004 | | 0.005 | | 0.006 | | 0.007 | | 0.008 | | 0.009 | | 0.010 | | 0.020 | | 0.030 | | 0.050 | | 0.070 | | 0.100 | | 1.000 | |  |  |  |  |  |  |  |
| Raw data | | | 0.290 | | 0.012 | | 0.243 | | 0.017 | | 0.077 | | 0.139 | | 0.000 | | -0.012 | | 0.185 | | 0.240 | | 0.020 | | 0.151 | | 0.406 | | 0.097 | | 0.170 | | 0.352 | | 0.406 | | 0.149 | | 0.130 | 0.874 | |
| LOG | | | 0.247 | | 0.222 | | 0.051 | | 0.123 | | 0.095 | | 0.106 | | 0.346 | | 0.082 | | 0.000 | | 0.218 | | 0.275 | | 0.143 | | 0.360 | | 0.103 | | 0.207 | | 0.280 | | 0.360 | | 0.179 | | 0.106 | 0.595 | |
| NST | | | 0.274 | | 0.286 | | 0.109 | | 0.271 | | 0.091 | | 0.457 | | 0.059 | | 0.324 | | 0.214 | | 0.245 | | 0.396 | | 0.332 | | 0.188 | | 0.327 | | 0.180 | | 0.378 | | 0.457 | | 0.258 | | 0.113 | 0.437 | |
| QN | | | 0.022 | | 0.210 | | 0.095 | | -0.026 | | 0.295 | | 0.400 | | 0.128 | | 0.256 | | 0.034 | | 0.353 | | 0.077 | | 0.060 | | 0.209 | | 0.038 | | 0.044 | | 0.471 | | 0.471 | | 0.167 | | 0.151 | 0.908 | |
| Z | | | 0.218 | | 0.124 | | 0.046 | | 0.152 | | 0.136 | | 0.212 | | -0.012 | | 0.000 | | 0.054 | | 0.171 | | 0.293 | | 0.221 | | 0.163 | | 0.304 | | 0.262 | | 0.038 | | 0.304 | | 0.149 | | 0.101 | 0.679 | |
| NPN | | | -0.004 | | 0.341 | | 0.151 | | 0.113 | | 0.006 | | 0.323 | | 0.170 | | 0.030 | | 0.126 | | 0.053 | | 0.249 | | 0.203 | | 0.250 | | 0.246 | | 0.262 | | 0.375 | | 0.375 | | 0.181 | | 0.120 | 0.665 | |
| Balanced Accuracy | | | | | **RF** | | | | | | | | | | | | | | | | | | | | | | | | | | | | | | | | | | | | |
| RF | | |  | |  | |  | |  | |  | |  | |  | |  | |  | |  | |  | |  | |  | |  | |  | |  | |  | |  | |  |  | |
| gene numbers | | | 11408 | | 11790 | | 12019 | | 12189 | | 12303 | | 12427 | | 12548 | | 12621 | | 12690 | | 12757 | | 13155 | | 13409 | | 13744 | | 13955 | | 14197 | | 15672 | | max | | Mean | | Standard Deviation | Coefficient of Variation | |
| DEG thresholds | | | 0.001 | | 0.002 | | 0.003 | | 0.004 | | 0.005 | | 0.006 | | 0.007 | | 0.008 | | 0.009 | | 0.010 | | 0.020 | | 0.030 | | 0.050 | | 0.070 | | 0.100 | | 1.000 | |  |  |  |  |  |  |  |
| Raw data | | | 0.348 | | 0.205 | | 0.352 | | 0.215 | | 0.228 | | 0.297 | | 0.200 | | 0.196 | | 0.280 | | 0.397 | | 0.210 | | 0.266 | | 0.404 | | 0.248 | | 0.297 | | 0.389 | | 0.404 | | 0.283 | | 0.075 | 0.263 | |
| LOG | | | 0.321 | | 0.297 | | 0.235 | | 0.265 | | 0.240 | | 0.238 | | 0.377 | | 0.265 | | 0.200 | | 0.286 | | 0.339 | | 0.243 | | 0.397 | | 0.274 | | 0.301 | | 0.338 | | 0.397 | | 0.289 | | 0.055 | 0.189 | |
| NST | | | 0.343 | | 0.395 | | 0.263 | | 0.385 | | 0.265 | | 0.439 | | 0.242 | | 0.414 | | 0.363 | | 0.308 | | 0.416 | | 0.391 | | 0.358 | | 0.369 | | 0.312 | | 0.419 | | 0.439 | | 0.355 | | 0.061 | 0.172 | |
| QN | | | 0.224 | | 0.307 | | 0.267 | | 0.198 | | 0.475 | | 0.439 | | 0.254 | | 0.281 | | 0.214 | | 0.391 | | 0.235 | | 0.221 | | 0.357 | | 0.214 | | 0.216 | | 0.453 | | 0.475 | | 0.297 | | 0.096 | 0.322 | |
| Z | | | 0.314 | | 0.257 | | 0.224 | | 0.275 | | 0.253 | | 0.283 | | 0.196 | | 0.200 | | 0.229 | | 0.329 | | 0.364 | | 0.317 | | 0.283 | | 0.399 | | 0.320 | | 0.199 | | 0.399 | | 0.278 | | 0.060 | 0.218 | |
| NPN | | | 0.200 | | 0.381 | | 0.322 | | 0.294 | | 0.205 | | 0.423 | | 0.338 | | 0.227 | | 0.279 | | 0.241 | | 0.440 | | 0.363 | | 0.371 | | 0.341 | | 0.344 | | 0.469 | | 0.469 | | 0.327 | | 0.082 | 0.250 | |
|  |  | |  | |  | |  | |  | |  | |  | |  | |  | |  | |  | |  | |  | |  | |  | |  | |  | |  | |  | | |  |  |
| Kappa | | | **LR** | | | | | | | | | | | | | | | | | | | | | | | | | | | | | | | | | | | | | | |
| gene numbers | | | 11408 | | 11790 | | 12019 | | 12189 | | 12303 | | 12427 | | 12548 | | 12621 | | 12690 | | 12757 | | 13155 | | 13409 | | 13744 | | 13955 | | 14197 | | 15672 | | max | | Mean | | Standard Deviation | Coefficient of Variation | |
| DEG thresholds | | | 0.001 | | 0.002 | | 0.003 | | 0.004 | | 0.005 | | 0.006 | | 0.007 | | 0.008 | | 0.009 | | 0.010 | | 0.020 | | 0.030 | | 0.050 | | 0.070 | | 0.100 | | 1.000 | |  |  |  |  |  |  |  |
| Raw data | | | 0.210 | | 0.000 | | 0.000 | | 0.045 | | 0.202 | | 0.096 | | 0.012 | | 0.032 | | 0.220 | | 0.088 | | 0.144 | | 0.399 | | 0.107 | | 0.232 | | 0.012 | | 0.000 | | 0.399 | | 0.113 | | 0.115 | 1.018 | |
| LOG | | | 0.272 | | 0.024 | | 0.000 | | 0.149 | | 0.224 | | 0.205 | | 0.000 | | 0.217 | | 0.000 | | 0.147 | | 0.028 | | 0.190 | | 0.177 | | 0.052 | | 0.144 | | 0.259 | | 0.272 | | 0.131 | | 0.098 | 0.751 | |
| NST | | | 0.141 | | 0.020 | | 0.000 | | 0.197 | | 0.259 | | 0.182 | | 0.064 | | 0.146 | | 0.075 | | 0.063 | | 0.086 | | 0.074 | | 0.229 | | 0.051 | | 0.239 | | 0.246 | | 0.259 | | 0.130 | | 0.086 | 0.665 | |
| QN | | | 0.104 | | 0.131 | | 0.000 | | 0.185 | | 0.220 | | 0.190 | | 0.024 | | 0.211 | | 0.000 | | 0.000 | | 0.000 | | 0.012 | | 0.082 | | 0.012 | | 0.066 | | 0.000 | | 0.220 | | 0.077 | | 0.085 | 1.095 | |
| Z | | | 0.059 | | 0.044 | | 0.000 | | 0.235 | | 0.241 | | 0.222 | | 0.174 | | 0.239 | | 0.075 | | 0.214 | | 0.193 | | 0.303 | | 0.242 | | 0.095 | | 0.063 | | 0.048 | | 0.303 | | 0.153 | | 0.095 | 0.621 | |
| NPN | | | 0.171 | | 0.125 | | 0.000 | | 0.185 | | 0.253 | | 0.205 | | 0.024 | | 0.036 | | 0.184 | | 0.222 | | 0.188 | | 0.204 | | 0.231 | | 0.120 | | 0.241 | | 0.036 | | 0.253 | | 0.152 | | 0.084 | 0.556 | |
| Balanced Accuracy | | | | | **LR** | | | | | | | | | | | | | | | | | | | | | | | | | | | | | | | | | | | | |
| gene numbers | | | 11408 | | 11790 | | 12019 | | 12189 | | 12303 | | 12427 | | 12548 | | 12621 | | 12690 | | 12757 | | 13155 | | 13409 | | 13744 | | 13955 | | 14197 | | 15672 | | max | | Mean | | Standard Deviation | Coefficient of Variation | |
| DEG thresholds | | | 0.001 | | 0.002 | | 0.003 | | 0.004 | | 0.005 | | 0.006 | | 0.007 | | 0.008 | | 0.009 | | 0.010 | | 0.020 | | 0.030 | | 0.050 | | 0.070 | | 0.100 | | 1.000 | |  |  |  |  |  |  |  |
| Raw data | | | 0.358 | | 0.200 | | 0.200 | | 0.235 | | 0.363 | | 0.275 | | 0.209 | | 0.225 | | 0.375 | | 0.268 | | 0.318 | | 0.497 | | 0.284 | | 0.384 | | 0.209 | | 0.200 | | 0.497 | | 0.288 | | 0.088 | 0.305 | |
| LOG | | | 0.408 | | 0.219 | | 0.200 | | 0.313 | | 0.375 | | 0.360 | | 0.200 | | 0.372 | | 0.200 | | 0.314 | | 0.222 | | 0.355 | | 0.368 | | 0.242 | | 0.314 | | 0.400 | | 0.408 | | 0.304 | | 0.078 | 0.255 | |
| NST | | | 0.309 | | 0.216 | | 0.200 | | 0.357 | | 0.400 | | 0.345 | | 0.250 | | 0.315 | | 0.258 | | 0.250 | | 0.272 | | 0.258 | | 0.374 | | 0.239 | | 0.394 | | 0.394 | | 0.400 | | 0.302 | | 0.068 | 0.224 | |
| QN | | | 0.307 | | 0.300 | | 0.200 | | 0.352 | | 0.370 | | 0.350 | | 0.218 | | 0.374 | | 0.200 | | 0.200 | | 0.200 | | 0.209 | | 0.264 | | 0.209 | | 0.252 | | 0.200 | | 0.374 | | 0.263 | | 0.068 | 0.261 | |
| Z | | | 0.246 | | 0.236 | | 0.200 | | 0.381 | | 0.388 | | 0.375 | | 0.334 | | 0.397 | | 0.260 | | 0.377 | | 0.354 | | 0.449 | | 0.394 | | 0.271 | | 0.248 | | 0.241 | | 0.449 | | 0.322 | | 0.077 | 0.238 | |
| NPN | | | 0.326 | | 0.302 | | 0.200 | | 0.353 | | 0.400 | | 0.355 | | 0.219 | | 0.227 | | 0.341 | | 0.375 | | 0.346 | | 0.363 | | 0.381 | | 0.298 | | 0.394 | | 0.227 | | 0.400 | | 0.319 | | 0.067 | 0.209 | |
|  | | |  | |  | |  | |  | |  | |  | |  | |  | |  | |  | |  | |  | |  | |  | |  | |  | |  | |  | |  |  | |
| Kappa | | |  | | **MLP** | | | | | | | | | | | | | | | | | | | | | | | | | | | | | | | | | | | | |
| gene numbers | | | 11408 | | 11790 | | 12019 | | 12189 | | 12303 | | 12427 | | 12548 | | 12621 | | 12690 | | 12757 | | 13155 | | 13409 | | 13744 | | 13955 | | 14197 | | 15672 | | max | | Mean | | Standard Deviation | Coefficient of Variation | |
| DEG thresholds | | | 0.001 | | 0.002 | | 0.003 | | 0.004 | | 0.005 | | 0.006 | | 0.007 | | 0.008 | | 0.009 | | 0.010 | | 0.020 | | 0.030 | | 0.050 | | 0.070 | | 0.100 | | 1.000 | |  |  |  |  |  |  |  |
| Raw data | | | 0.048 | | 0.000 | | 0.000 | | 0.232 | | 0.233 | | 0.237 | | 0.125 | | 0.149 | | 0.097 | | 0.249 | | 0.255 | | 0.349 | | 0.291 | | 0.233 | | 0.032 | | 0.000 | | 0.349 | | 0.158 | | 0.116 | 0.735 | |
| LOG | | | 0.187 | | 0.346 | | 0.292 | | 0.262 | | 0.362 | | 0.114 | | 0.024 | | 0.228 | | 0.320 | | 0.095 | | 0.258 | | 0.179 | | 0.087 | | 0.012 | | 0.228 | | 0.240 | | 0.362 | | 0.202 | | 0.109 | 0.540 | |
| NST | | | 0.201 | | 0.325 | | 0.240 | | 0.251 | | 0.235 | | 0.233 | | 0.241 | | 0.256 | | 0.326 | | 0.233 | | 0.178 | | 0.024 | | 0.326 | | 0.157 | | 0.252 | | 0.075 | | 0.326 | | 0.222 | | 0.083 | 0.374 | |
| QN | | | 0.322 | | 0.179 | | 0.260 | | 0.208 | | 0.260 | | 0.214 | | 0.189 | | 0.123 | | 0.000 | | 0.000 | | 0.116 | | 0.232 | | 0.185 | | 0.237 | | 0.117 | | 0.024 | | 0.322 | | 0.167 | | 0.096 | 0.577 | |
| Z | | | 0.204 | | 0.117 | | 0.056 | | 0.250 | | 0.105 | | 0.178 | | 0.247 | | 0.175 | | 0.228 | | 0.233 | | 0.220 | | 0.298 | | 0.218 | | 0.232 | | 0.024 | | 0.113 | | 0.298 | | 0.181 | | 0.077 | 0.424 | |
| NPN | | | 0.400 | | 0.086 | | 0.131 | | 0.044 | | 0.246 | | 0.063 | | 0.256 | | 0.279 | | 0.205 | | 0.210 | | 0.361 | | 0.008 | | 0.053 | | 0.290 | | 0.016 | | 0.262 | | 0.400 | | 0.182 | | 0.126 | 0.691 | |
| Balanced Accuracy | | | | | **MLP** | | | | | | | | | | | | | | | | | | | | | | | | | | | | | | | | | | | | |
| gene numbers | | | 11408 | | 11790 | | 12019 | | 12189 | | 12303 | | 12427 | | 12548 | | 12621 | | 12690 | | 12757 | | 13155 | | 13409 | | 13744 | | 13955 | | 14197 | | 15672 | | max | | Mean | | Standard Deviation | Coefficient of Variation | |
| DEG thresholds | | | 0.001 | | 0.002 | | 0.003 | | 0.004 | | 0.005 | | 0.006 | | 0.007 | | 0.008 | | 0.009 | | 0.010 | | 0.020 | | 0.030 | | 0.050 | | 0.070 | | 0.100 | | 1.000 | |  |  |  |  |  |  |  |
| Raw data | | | 0.236 | | 0.200 | | 0.200 | | 0.382 | | 0.385 | | 0.381 | | 0.297 | | 0.319 | | 0.277 | | 0.391 | | 0.452 | | 0.549 | | 0.504 | | 0.384 | | 0.225 | | 0.200 | | 0.549 | | 0.336 | | 0.110 | 0.328 | |
| LOG | | | 0.347 | | 0.550 | | 0.465 | | 0.400 | | 0.554 | | 0.289 | | 0.218 | | 0.382 | | 0.522 | | 0.274 | | 0.400 | | 0.345 | | 0.291 | | 0.210 | | 0.380 | | 0.385 | | 0.554 | | 0.376 | | 0.107 | 0.285 | |
| NST | | | 0.353 | | 0.498 | | 0.387 | | 0.400 | | 0.382 | | 0.381 | | 0.387 | | 0.397 | | 0.512 | | 0.384 | | 0.351 | | 0.219 | | 0.481 | | 0.319 | | 0.400 | | 0.259 | | 0.512 | | 0.382 | | 0.077 | 0.201 | |
| QN | | | 0.561 | | 0.382 | | 0.428 | | 0.371 | | 0.393 | | 0.369 | | 0.343 | | 0.300 | | 0.200 | | 0.200 | | 0.330 | | 0.382 | | 0.345 | | 0.382 | | 0.291 | | 0.218 | | 0.561 | | 0.343 | | 0.091 | 0.265 | |
| Z | | | 0.360 | | 0.294 | | 0.242 | | 0.393 | | 0.275 | | 0.363 | | 0.388 | | 0.334 | | 0.384 | | 0.390 | | 0.376 | | 0.457 | | 0.406 | | 0.375 | | 0.218 | | 0.294 | | 0.457 | | 0.347 | | 0.065 | 0.187 | |
| NPN | | | 0.563 | | 0.270 | | 0.306 | | 0.237 | | 0.394 | | 0.248 | | 0.400 | | 0.450 | | 0.348 | | 0.366 | | 0.546 | | 0.206 | | 0.200 | | 0.466 | | 0.212 | | 0.400 | | 0.563 | | 0.351 | | 0.118 | 0.336 | |
|  | | |  | |  | |  | |  | |  | |  | |  | |  | |  | |  | |  | |  | |  | |  | |  | |  | |  | |  | |  |  | |
| Kappa | | | **XGB** | | | | | | | | | | | | | | | | | | | | | | | | | | | | | | | | | | | | | | |
| gene numbers | | | 11408 | | 11790 | | 12019 | | 12189 | | 12303 | | 12427 | | 12548 | | 12621 | | 12690 | | 12757 | | 13155 | | 13409 | | 13744 | | 13955 | | 14197 | | 15672 | | max | | Mean | | Standard Deviation | Coefficient of Variation | |
| DEG thresholds | | | 0.001 | | 0.002 | | 0.003 | | 0.004 | | 0.005 | | 0.006 | | 0.007 | | 0.008 | | 0.009 | | 0.010 | | 0.020 | | 0.030 | | 0.050 | | 0.070 | | 0.100 | | 1.000 | |  |  |  |  |  |  |  |
| Raw data | | | 0.382 | | 0.308 | | 0.306 | | 0.261 | | 0.218 | | 0.057 | | 0.236 | | 0.080 | | 0.187 | | 0.164 | | 0.180 | | 0.277 | | 0.203 | | 0.103 | | 0.429 | | 0.085 | | 0.429 | | 0.217 | | 0.108 | 0.497 | |
| LOG | | | 0.040 | | 0.191 | | 0.003 | | 0.307 | | 0.250 | | 0.014 | | 0.242 | | 0.190 | | 0.053 | | 0.169 | | 0.157 | | 0.100 | | 0.012 | | -0.031 | | 0.095 | | 0.005 | | 0.307 | | 0.112 | | 0.105 | 0.931 | |
| NST | | | 0.189 | | 0.217 | | 0.105 | | 0.089 | | 0.308 | | 0.317 | | 0.000 | | 0.036 | | 0.250 | | 0.075 | | 0.068 | | 0.376 | | 0.389 | | 0.497 | | 0.179 | | 0.560 | | 0.560 | | 0.228 | | 0.168 | 0.734 | |
| QN | | | 0.174 | | 0.165 | | 0.019 | | -0.014 | | 0.097 | | 0.038 | | 0.239 | | 0.040 | | 0.080 | | 0.113 | | 0.254 | | 0.017 | | 0.358 | | 0.091 | | 0.090 | | 0.140 | | 0.358 | | 0.119 | | 0.100 | 0.843 | |
| Z | | | 0.104 | | 0.308 | | 0.030 | | 0.206 | | 0.195 | | 0.151 | | 0.040 | | 0.084 | | 0.172 | | 0.276 | | 0.106 | | 0.063 | | 0.190 | | 0.102 | | 0.328 | | 0.008 | | 0.328 | | 0.148 | | 0.098 | 0.665 | |
| NPN | | | 0.435 | | 0.291 | | 0.298 | | 0.030 | | 0.361 | | 0.353 | | 0.197 | | 0.106 | | 0.156 | | 0.520 | | 0.270 | | 0.362 | | 0.245 | | 0.416 | | 0.416 | | 0.278 | | 0.520 | | 0.296 | | 0.129 | 0.436 | |
| Balanced Accuracy | | | | | **XGB** | | | | | | | | | | | | | | | | | | | | | | | | | | | | | | | | | | | | |
| gene numbers | | | 11408 | | 11790 | | 12019 | | 12189 | | 12303 | | 12427 | | 12548 | | 12621 | | 12690 | | 12757 | | 13155 | | 13409 | | 13744 | | 13955 | | 14197 | | 15672 | | max | | Mean | | Standard Deviation | Coefficient of Variation | |
| DEG thresholds | | | 0.001 | | 0.002 | | 0.003 | | 0.004 | | 0.005 | | 0.006 | | 0.007 | | 0.008 | | 0.009 | | 0.010 | | 0.020 | | 0.030 | | 0.050 | | 0.070 | | 0.100 | | 1.000 | |  |  |  |  |  |  |  |
| Raw data | | | 0.402 | | 0.457 | | 0.408 | | 0.408 | | 0.372 | | 0.243 | | 0.387 | | 0.333 | | 0.348 | | 0.319 | | 0.348 | | 0.406 | | 0.428 | | 0.293 | | 0.484 | | 0.279 | | 0.484 | | 0.370 | | 0.066 | 0.178 | |
| LOG | | | 0.233 | | 0.347 | | 0.207 | | 0.414 | | 0.400 | | 0.217 | | 0.397 | | 0.340 | | 0.239 | | 0.288 | | 0.234 | | 0.266 | | 0.153 | | 0.141 | | 0.265 | | 0.211 | | 0.414 | | 0.272 | | 0.085 | 0.314 | |
| NST | | | 0.392 | | 0.373 | | 0.337 | | 0.288 | | 0.504 | | 0.481 | | 0.218 | | 0.230 | | 0.412 | | 0.316 | | 0.289 | | 0.513 | | 0.503 | | 0.494 | | 0.364 | | 0.571 | | 0.571 | | 0.393 | | 0.109 | 0.277 | |
| QN | | | 0.503 | | 0.363 | | 0.224 | | 0.157 | | 0.268 | | 0.224 | | 0.527 | | 0.213 | | 0.189 | | 0.345 | | 0.339 | | 0.215 | | 0.454 | | 0.165 | | 0.294 | | 0.268 | | 0.527 | | 0.297 | | 0.116 | 0.392 | |
| Z | | | 0.332 | | 0.447 | | 0.250 | | 0.396 | | 0.349 | | 0.330 | | 0.295 | | 0.261 | | 0.337 | | 0.357 | | 0.251 | | 0.242 | | 0.394 | | 0.290 | | 0.402 | | 0.202 | | 0.447 | | 0.321 | | 0.069 | 0.215 | |
| NPN | | | 0.542 | | 0.381 | | 0.480 | | 0.226 | | 0.527 | | 0.466 | | 0.494 | | 0.368 | | 0.335 | | 0.501 | | 0.451 | | 0.610 | | 0.396 | | 0.519 | | 0.533 | | 0.457 | | 0.610 | | 0.455 | | 0.095 | 0.208 | |
