# Supplementary material for: Normalization and Selecting Non-Differentially Expressed Genes Improve Machine Learning Modelling of Cross-Platform Transcriptomic Data
Source: Trans Artif Intell. Author manuscript; Available in PMC 2025 Jul 8. (PMC12235674; doi:10.53941/tai.2025.100005)
Supplement: Supplementary [file NIHMS2087281-supplement-Supplementary.zip › Supplementary table 3.docx]

| Supplementary table 3. Average classification performance results (**Kappa**) on data constructed using DEG and NDEG genes selected via one-way ANOVA. (**Model-S**) | | | | | | | | | | | | | | | | | | | | | |
| --- | --- | --- | --- | --- | --- | --- | --- | --- | --- | --- | --- | --- | --- | --- | --- | --- | --- | --- | --- | --- | --- |
| SVM |  |  |  |  |  |  |  |  |  |  |  |  |  |  |  |  |  |  |  |  |  |
| LOG_NPN_Z | DEG thresholds | 0.001 | 0.002 | 0.003 | 0.004 | 0.005 | 0.006 | 0.007 | 0.008 | 0.009 | 0.010 | 0.020 | 0.030 | 0.050 | 0.070 | 0.100 | 1.000 | max | Mean | Standard Deviation | Coefficient of Variation |
| NDEG thresholds | gene numbers | 10124 | 10523 | 10756 | 10936 | 11079 | 11183 | 11282 | 11366 | 11447 | 11516 | 12001 | 12294 | 12690 | 12934 | 13236 | 15672 |  |  |  |  |
| 0.980 | 11 | 0.427 | 0.205 | 0.226 | 0.228 | 0.241 | 0.315 | 0.339 | 0.333 | 0.354 | 0.497 | 0.405 | 0.257 | 0.264 | 0.368 | 0.289 | 0.218 | 0.497 | 0.310 | 0.085 | 0.273 |
| 0.950 | 49 | 0.370 | 0.288 | 0.288 | 0.252 | 0.212 | 0.225 | 0.288 | 0.242 | 0.219 | 0.288 | 0.409 | 0.369 | 0.369 | 0.257 | 0.258 | 0.234 | 0.409 | 0.286 | 0.062 | 0.216 |
| 0.920 | 94 | 0.329 | 0.225 | 0.329 | 0.209 | 0.276 | 0.226 | 0.289 | 0.344 | 0.232 | 0.363 | 0.384 | 0.240 | 0.294 | 0.190 | 0.244 | 0.313 | 0.384 | 0.280 | 0.059 | 0.210 |
| 0.900 | 133 | 0.301 | 0.243 | 0.332 | 0.312 | 0.175 | 0.160 | 0.230 | 0.327 | 0.256 | 0.294 | 0.285 | 0.354 | 0.308 | 0.190 | 0.361 | 0.213 | 0.361 | 0.271 | 0.064 | 0.234 |
| 0.850 | 253 | 0.334 | 0.448 | 0.286 | 0.316 | 0.284 | 0.292 | 0.319 | 0.279 | 0.212 | 0.227 | 0.190 | 0.359 | 0.379 | 0.261 | 0.280 | 0.239 | 0.448 | 0.294 | 0.066 | 0.223 |
| Mean | | 0.352 | 0.282 | 0.292 | 0.264 | 0.238 | 0.244 | 0.293 | 0.305 | 0.255 | 0.334 | 0.335 | 0.316 | 0.323 | 0.253 | 0.286 | 0.243 |  | 0.288 |  |  |
| Standard Deviation | | 0.049 | 0.098 | 0.043 | 0.049 | 0.045 | 0.062 | 0.041 | 0.043 | 0.058 | 0.103 | 0.095 | 0.062 | 0.049 | 0.073 | 0.045 | 0.040 |  |  |  |  |
| LOG_RQN | DEG thresholds | 0.001 | 0.002 | 0.003 | 0.004 | 0.005 | 0.006 | 0.007 | 0.008 | 0.009 | 0.010 | 0.020 | 0.030 | 0.050 | 0.070 | 0.100 | 1.000 | max | Mean | Standard Deviation | Coefficient of Variation |
| NDEG thresholds | gene numbers | 10124 | 10523 | 10756 | 10936 | 11079 | 11183 | 11282 | 11366 | 11447 | 11516 | 12001 | 12294 | 12690 | 12934 | 13236 | 15672 |  |  |  |  |
| 0.980 | 11 | 0.801 | 0.788 | 0.780 | 0.821 | 0.811 | 0.808 | 0.849 | 0.806 | 0.776 | 0.777 | 0.693 | 0.812 | 0.798 | 0.812 | 0.781 | 0.803 | 0.849 | 0.795 | 0.033 | 0.042 |
| 0.950 | 49 | 0.761 | 0.761 | 0.816 | 0.788 | 0.755 | 0.759 | 0.819 | 0.786 | 0.730 | 0.730 | 0.742 | 0.788 | 0.781 | 0.756 | 0.801 | 0.824 | 0.824 | 0.775 | 0.030 | 0.039 |
| 0.920 | 94 | 0.650 | 0.707 | 0.762 | 0.730 | 0.783 | 0.792 | 0.804 | 0.789 | 0.756 | 0.725 | 0.765 | 0.693 | 0.713 | 0.758 | 0.778 | 0.772 | 0.804 | 0.749 | 0.042 | 0.056 |
| 0.900 | 133 | 0.724 | 0.700 | 0.749 | 0.635 | 0.760 | 0.808 | 0.782 | 0.804 | 0.780 | 0.648 | 0.796 | 0.643 | 0.614 | 0.679 | 0.717 | 0.623 | 0.808 | 0.716 | 0.069 | 0.096 |
| 0.850 | 253 | 0.668 | 0.666 | 0.655 | 0.723 | 0.691 | 0.663 | 0.516 | 0.558 | 0.721 | 0.533 | 0.450 | 0.504 | 0.638 | 0.644 | 0.621 | 0.389 | 0.723 | 0.602 | 0.099 | 0.165 |
| Mean | | 0.721 | 0.725 | 0.752 | 0.739 | 0.760 | 0.766 | 0.754 | 0.749 | 0.753 | 0.683 | 0.689 | 0.688 | 0.709 | 0.730 | 0.740 | 0.682 |  | 0.727 |  |  |
| Standard Deviation | | 0.063 | 0.050 | 0.060 | 0.071 | 0.045 | 0.061 | 0.135 | 0.107 | 0.026 | 0.096 | 0.139 | 0.124 | 0.082 | 0.068 | 0.074 | 0.182 |  |  |  |  |
| LOG_RQN_Z | DEG thresholds | 0.001 | 0.002 | 0.003 | 0.004 | 0.005 | 0.006 | 0.007 | 0.008 | 0.009 | 0.010 | 0.020 | 0.030 | 0.050 | 0.070 | 0.100 | 1.000 | max | Mean | Standard Deviation | Coefficient of Variation |
| NDEG thresholds | gene numbers | 10124 | 10523 | 10756 | 10936 | 11079 | 11183 | 11282 | 11366 | 11447 | 11516 | 12001 | 12294 | 12690 | 12934 | 13236 | 15672 |  |  |  |  |
| 0.980 | 11 | 0.788 | 0.808 | 0.801 | 0.783 | 0.810 | 0.811 | 0.808 | 0.829 | 0.778 | 0.793 | 0.682 | 0.787 | 0.721 | 0.818 | 0.785 | 0.797 | 0.829 | 0.787 | 0.037 | 0.047 |
| 0.950 | 49 | 0.737 | 0.758 | 0.807 | 0.797 | 0.772 | 0.759 | 0.818 | 0.782 | 0.728 | 0.745 | 0.767 | 0.772 | 0.739 | 0.751 | 0.796 | 0.820 | 0.820 | 0.772 | 0.029 | 0.038 |
| 0.920 | 94 | 0.697 | 0.739 | 0.744 | 0.755 | 0.751 | 0.806 | 0.782 | 0.762 | 0.701 | 0.736 | 0.716 | 0.659 | 0.732 | 0.751 | 0.770 | 0.751 | 0.806 | 0.741 | 0.035 | 0.048 |
| 0.900 | 133 | 0.703 | 0.760 | 0.712 | 0.707 | 0.781 | 0.783 | 0.780 | 0.756 | 0.787 | 0.669 | 0.778 | 0.680 | 0.620 | 0.707 | 0.705 | 0.658 | 0.787 | 0.724 | 0.052 | 0.072 |
| 0.850 | 253 | 0.690 | 0.703 | 0.639 | 0.706 | 0.726 | 0.675 | 0.579 | 0.586 | 0.703 | 0.541 | 0.470 | 0.519 | 0.688 | 0.649 | 0.638 | 0.547 | 0.726 | 0.629 | 0.078 | 0.125 |
| Mean | | 0.723 | 0.753 | 0.740 | 0.749 | 0.768 | 0.767 | 0.753 | 0.743 | 0.739 | 0.697 | 0.682 | 0.683 | 0.700 | 0.735 | 0.738 | 0.714 |  | 0.730 |  |  |
| Standard Deviation | | 0.040 | 0.038 | 0.069 | 0.042 | 0.032 | 0.055 | 0.099 | 0.093 | 0.041 | 0.097 | 0.125 | 0.107 | 0.049 | 0.063 | 0.066 | 0.112 |  |  |  |  |
| LOG_NICG_Z | DEG thresholds | 0.001 | 0.002 | 0.003 | 0.004 | 0.005 | 0.006 | 0.007 | 0.008 | 0.009 | 0.010 | 0.020 | 0.030 | 0.050 | 0.070 | 0.100 | 1.000 | max | Mean | Standard Deviation | Coefficient of Variation |
| NDEG thresholds | gene numbers | 10124 | 10523 | 10756 | 10936 | 11079 | 11183 | 11282 | 11366 | 11447 | 11516 | 12001 | 12294 | 12690 | 12934 | 13236 | 15672 |  |  |  |  |
| 0.980 | 11 | 0.211 | 0.167 | 0.274 | 0.160 | 0.274 | 0.196 | 0.161 | 0.255 | 0.208 | 0.321 | 0.276 | 0.143 | 0.299 | 0.384 | 0.397 | 0.229 | 0.397 | 0.247 | 0.077 | 0.313 |
| 0.950 | 49 | 0.175 | 0.209 | 0.266 | 0.069 | 0.188 | 0.116 | 0.212 | 0.233 | 0.271 | 0.129 | 0.394 | 0.295 | 0.330 | 0.245 | 0.209 | 0.217 | 0.394 | 0.222 | 0.081 | 0.364 |
| 0.920 | 94 | 0.179 | 0.189 | 0.349 | 0.130 | 0.168 | 0.243 | 0.217 | 0.393 | 0.221 | 0.441 | 0.357 | 0.238 | 0.338 | 0.245 | 0.195 | 0.272 | 0.441 | 0.261 | 0.089 | 0.342 |
| 0.900 | 133 | 0.240 | 0.162 | 0.235 | 0.246 | 0.195 | 0.220 | 0.188 | 0.353 | 0.320 | 0.353 | 0.356 | 0.306 | 0.302 | 0.194 | 0.392 | 0.245 | 0.392 | 0.269 | 0.071 | 0.265 |
| 0.850 | 253 | 0.050 | 0.334 | 0.439 | 0.153 | 0.192 | 0.159 | 0.185 | 0.350 | 0.182 | 0.286 | 0.209 | 0.248 | 0.263 | 0.167 | 0.131 | 0.299 | 0.439 | 0.228 | 0.097 | 0.427 |
| Mean | | 0.171 | 0.212 | 0.313 | 0.152 | 0.203 | 0.187 | 0.192 | 0.317 | 0.240 | 0.306 | 0.319 | 0.246 | 0.306 | 0.247 | 0.265 | 0.252 |  | 0.245 |  |  |
| Standard Deviation | | 0.073 | 0.071 | 0.082 | 0.064 | 0.041 | 0.050 | 0.023 | 0.069 | 0.055 | 0.114 | 0.075 | 0.064 | 0.030 | 0.084 | 0.122 | 0.034 |  |  |  |  |
|  |  |  |  |  |  |  |  |  |  |  |  |  |  |  |  |  |  |  |  |  |  |
| RF |  |  |  |  |  |  |  |  |  |  |  |  |  |  |  |  |  |  |  |  |  |
| LOG_NPN_Z | DEG thresholds | 0.001 | 0.002 | 0.003 | 0.004 | 0.005 | 0.006 | 0.007 | 0.008 | 0.009 | 0.010 | 0.020 | 0.030 | 0.050 | 0.070 | 0.100 | 1.000 | max | Mean | Standard Deviation | Coefficient of Variation |
| NDEG thresholds | gene numbers | 10124 | 10523 | 10756 | 10936 | 11079 | 11183 | 11282 | 11366 | 11447 | 11516 | 12001 | 12294 | 12690 | 12934 | 13236 | 15672 |  |  |  |  |
| 0.980 | 11 | 0.220 | 0.359 | 0.259 | 0.266 | 0.254 | 0.211 | 0.323 | 0.250 | 0.065 | 0.324 | 0.313 | 0.292 | 0.196 | 0.330 | 0.161 | 0.218 | 0.359 | 0.253 | 0.075 | 0.295 |
| 0.950 | 49 | 0.289 | 0.298 | 0.221 | 0.289 | 0.281 | 0.345 | 0.258 | 0.216 | 0.284 | 0.291 | 0.122 | 0.172 | 0.295 | 0.298 | 0.314 | 0.322 | 0.345 | 0.268 | 0.058 | 0.217 |
| 0.920 | 94 | 0.264 | 0.220 | 0.261 | 0.156 | 0.330 | 0.247 | 0.299 | 0.266 | 0.290 | 0.406 | 0.187 | 0.117 | 0.265 | 0.176 | 0.252 | 0.211 | 0.406 | 0.247 | 0.070 | 0.285 |
| 0.900 | 133 | 0.162 | 0.113 | 0.168 | 0.304 | 0.145 | 0.080 | 0.281 | 0.310 | 0.214 | 0.290 | 0.220 | 0.223 | 0.313 | 0.224 | 0.215 | 0.274 | 0.313 | 0.221 | 0.072 | 0.326 |
| 0.850 | 253 | 0.216 | 0.198 | 0.286 | 0.330 | 0.268 | 0.137 | 0.171 | 0.206 | 0.184 | 0.253 | 0.195 | 0.138 | 0.191 | 0.077 | 0.228 | 0.183 | 0.330 | 0.204 | 0.062 | 0.303 |
| Mean | | 0.230 | 0.238 | 0.239 | 0.269 | 0.256 | 0.204 | 0.266 | 0.250 | 0.207 | 0.313 | 0.207 | 0.188 | 0.252 | 0.221 | 0.234 | 0.241 |  | 0.238 |  |  |
| Standard Deviation | | 0.049 | 0.095 | 0.046 | 0.067 | 0.068 | 0.102 | 0.059 | 0.042 | 0.091 | 0.058 | 0.069 | 0.071 | 0.056 | 0.101 | 0.056 | 0.056 |  |  |  |  |
| LOG_RQN | DEG thresholds | 0.001 | 0.002 | 0.003 | 0.004 | 0.005 | 0.006 | 0.007 | 0.008 | 0.009 | 0.010 | 0.020 | 0.030 | 0.050 | 0.070 | 0.100 | 1.000 | max | Mean | Standard Deviation | Coefficient of Variation |
| NDEG thresholds | gene numbers | 10124 | 10523 | 10756 | 10936 | 11079 | 11183 | 11282 | 11366 | 11447 | 11516 | 12001 | 12294 | 12690 | 12934 | 13236 | 15672 |  |  |  |  |
| 0.980 | 11 | 0.704 | 0.687 | 0.723 | 0.647 | 0.588 | 0.738 | 0.714 | 0.637 | 0.638 | 0.690 | 0.574 | 0.649 | 0.630 | 0.671 | 0.463 | 0.705 | 0.738 | 0.654 | 0.069 | 0.105 |
| 0.950 | 49 | 0.602 | 0.639 | 0.694 | 0.647 | 0.682 | 0.719 | 0.670 | 0.645 | 0.540 | 0.560 | 0.537 | 0.604 | 0.654 | 0.654 | 0.671 | 0.727 | 0.727 | 0.640 | 0.058 | 0.091 |
| 0.920 | 94 | 0.246 | 0.483 | 0.523 | 0.440 | 0.569 | 0.614 | 0.641 | 0.518 | 0.377 | 0.465 | 0.436 | 0.240 | 0.571 | 0.562 | 0.567 | 0.657 | 0.657 | 0.494 | 0.124 | 0.252 |
| 0.900 | 133 | 0.261 | 0.307 | 0.270 | 0.552 | 0.466 | 0.442 | 0.512 | 0.457 | 0.606 | 0.419 | 0.593 | 0.269 | 0.268 | 0.308 | 0.392 | 0.453 | 0.606 | 0.411 | 0.120 | 0.291 |
| 0.850 | 253 | 0.170 | 0.183 | 0.158 | 0.296 | 0.124 | 0.139 | 0.181 | 0.255 | 0.550 | 0.121 | 0.161 | 0.129 | 0.169 | 0.263 | 0.124 | 0.038 | 0.550 | 0.191 | 0.114 | 0.598 |
| Mean | | 0.396 | 0.460 | 0.474 | 0.516 | 0.486 | 0.530 | 0.544 | 0.502 | 0.542 | 0.451 | 0.460 | 0.378 | 0.458 | 0.492 | 0.443 | 0.516 |  | 0.478 |  |  |
| Standard Deviation | | 0.239 | 0.215 | 0.252 | 0.150 | 0.216 | 0.248 | 0.216 | 0.160 | 0.101 | 0.212 | 0.178 | 0.233 | 0.224 | 0.194 | 0.207 | 0.288 |  |  |  |  |
| LOG_RQN_Z | DEG thresholds | 0.001 | 0.002 | 0.003 | 0.004 | 0.005 | 0.006 | 0.007 | 0.008 | 0.009 | 0.010 | 0.020 | 0.030 | 0.050 | 0.070 | 0.100 | 1.000 | max | Mean | Standard Deviation | Coefficient of Variation |
| NDEG thresholds | gene numbers | 10124 | 10523 | 10756 | 10936 | 11079 | 11183 | 11282 | 11366 | 11447 | 11516 | 12001 | 12294 | 12690 | 12934 | 13236 | 15672 |  |  |  |  |
| 0.980 | 11 | 0.738 | 0.741 | 0.713 | 0.629 | 0.543 | 0.727 | 0.713 | 0.645 | 0.701 | 0.675 | 0.610 | 0.701 | 0.664 | 0.664 | 0.589 | 0.734 | 0.741 | 0.674 | 0.058 | 0.086 |
| 0.950 | 49 | 0.604 | 0.638 | 0.717 | 0.627 | 0.647 | 0.698 | 0.663 | 0.669 | 0.663 | 0.625 | 0.604 | 0.644 | 0.699 | 0.616 | 0.729 | 0.722 | 0.729 | 0.660 | 0.042 | 0.063 |
| 0.920 | 94 | 0.234 | 0.578 | 0.568 | 0.409 | 0.471 | 0.603 | 0.679 | 0.630 | 0.400 | 0.366 | 0.492 | 0.308 | 0.584 | 0.610 | 0.610 | 0.661 | 0.679 | 0.513 | 0.134 | 0.261 |
| 0.900 | 133 | 0.265 | 0.539 | 0.275 | 0.470 | 0.552 | 0.384 | 0.543 | 0.506 | 0.694 | 0.319 | 0.543 | 0.275 | 0.242 | 0.358 | 0.380 | 0.420 | 0.694 | 0.423 | 0.132 | 0.312 |
| 0.850 | 253 | 0.074 | 0.183 | 0.099 | 0.296 | 0.210 | 0.097 | 0.144 | 0.316 | 0.454 | 0.127 | 0.115 | 0.183 | 0.160 | 0.061 | 0.130 | 0.060 | 0.454 | 0.169 | 0.107 | 0.629 |
| Mean | | 0.383 | 0.536 | 0.474 | 0.486 | 0.485 | 0.502 | 0.549 | 0.553 | 0.583 | 0.422 | 0.473 | 0.422 | 0.470 | 0.462 | 0.488 | 0.519 |  | 0.488 |  |  |
| Standard Deviation | | 0.277 | 0.212 | 0.276 | 0.144 | 0.166 | 0.263 | 0.235 | 0.147 | 0.144 | 0.227 | 0.206 | 0.234 | 0.251 | 0.254 | 0.236 | 0.286 |  |  |  |  |
| LOG_NICG_Z | DEG thresholds | 0.001 | 0.002 | 0.003 | 0.004 | 0.005 | 0.006 | 0.007 | 0.008 | 0.009 | 0.010 | 0.020 | 0.030 | 0.050 | 0.070 | 0.100 | 1.000 | max | Mean | Standard Deviation | Coefficient of Variation |
| NDEG thresholds | gene numbers | 10124 | 10523 | 10756 | 10936 | 11079 | 11183 | 11282 | 11366 | 11447 | 11516 | 12001 | 12294 | 12690 | 12934 | 13236 | 15672 |  |  |  |  |
| 0.980 | 11 | 0.105 | 0.217 | 0.066 | 0.082 | 0.121 | 0.099 | 0.227 | 0.133 | 0.150 | 0.054 | 0.195 | 0.139 | 0.223 | 0.302 | 0.164 | 0.240 | 0.302 | 0.157 | 0.071 | 0.449 |
| 0.950 | 49 | 0.126 | 0.117 | 0.361 | 0.330 | 0.316 | 0.167 | 0.166 | 0.097 | 0.009 | 0.010 | 0.091 | 0.079 | 0.085 | 0.183 | 0.120 | 0.228 | 0.361 | 0.155 | 0.106 | 0.684 |
| 0.920 | 94 | 0.213 | 0.118 | 0.004 | 0.082 | 0.223 | 0.163 | 0.084 | 0.295 | 0.181 | 0.277 | 0.280 | 0.038 | 0.035 | 0.150 | 0.130 | 0.197 | 0.295 | 0.154 | 0.091 | 0.589 |
| 0.900 | 133 | 0.343 | 0.232 | 0.306 | 0.105 | 0.188 | 0.165 | 0.166 | 0.172 | 0.131 | 0.365 | 0.195 | 0.127 | 0.074 | 0.275 | 0.140 | 0.248 | 0.365 | 0.202 | 0.085 | 0.423 |
| 0.850 | 253 | 0.162 | 0.088 | 0.158 | 0.239 | 0.213 | 0.061 | 0.085 | 0.119 | 0.169 | 0.189 | 0.114 | 0.108 | 0.283 | 0.187 | 0.075 | 0.302 | 0.302 | 0.159 | 0.073 | 0.459 |
| Mean | | 0.190 | 0.154 | 0.179 | 0.167 | 0.212 | 0.131 | 0.146 | 0.163 | 0.128 | 0.179 | 0.175 | 0.098 | 0.140 | 0.219 | 0.126 | 0.243 |  | 0.166 |  |  |
| Standard Deviation | | 0.095 | 0.065 | 0.153 | 0.112 | 0.070 | 0.048 | 0.061 | 0.078 | 0.069 | 0.149 | 0.075 | 0.041 | 0.107 | 0.065 | 0.033 | 0.038 |  |  |  |  |
|  |  |  |  |  |  |  |  |  |  |  |  |  |  |  |  |  |  |  |  |  |  |
| LR |  |  |  |  |  |  |  |  |  |  |  |  |  |  |  |  |  |  |  |  |  |
| LOG_NPN_Z | DEG thresholds | 0.001 | 0.002 | 0.003 | 0.004 | 0.005 | 0.006 | 0.007 | 0.008 | 0.009 | 0.010 | 0.020 | 0.030 | 0.050 | 0.070 | 0.100 | 1.000 | max | Mean | Standard Deviation | Coefficient of Variation |
| NDEG thresholds | gene numbers | 10124 | 10523 | 10756 | 10936 | 11079 | 11183 | 11282 | 11366 | 11447 | 11516 | 12001 | 12294 | 12690 | 12934 | 13236 | 15672 |  |  |  |  |
| 0.980 | 11 | 0.488 | 0.478 | 0.549 | 0.425 | 0.498 | 0.539 | 0.532 | 0.475 | 0.552 | 0.554 | 0.527 | 0.440 | 0.532 | 0.516 | 0.527 | 0.467 | 0.554 | 0.506 | 0.040 | 0.079 |
| 0.950 | 49 | 0.518 | 0.470 | 0.505 | 0.451 | 0.486 | 0.535 | 0.466 | 0.469 | 0.513 | 0.530 | 0.537 | 0.488 | 0.491 | 0.505 | 0.492 | 0.406 | 0.537 | 0.491 | 0.034 | 0.070 |
| 0.920 | 94 | 0.458 | 0.452 | 0.469 | 0.424 | 0.480 | 0.502 | 0.442 | 0.479 | 0.539 | 0.488 | 0.498 | 0.517 | 0.477 | 0.497 | 0.513 | 0.440 | 0.539 | 0.480 | 0.031 | 0.065 |
| 0.900 | 133 | 0.470 | 0.472 | 0.508 | 0.431 | 0.480 | 0.487 | 0.459 | 0.452 | 0.477 | 0.513 | 0.527 | 0.542 | 0.468 | 0.504 | 0.475 | 0.433 | 0.542 | 0.481 | 0.031 | 0.065 |
| 0.850 | 253 | 0.458 | 0.498 | 0.502 | 0.449 | 0.478 | 0.495 | 0.469 | 0.465 | 0.504 | 0.516 | 0.384 | 0.504 | 0.454 | 0.465 | 0.479 | 0.401 | 0.516 | 0.470 | 0.037 | 0.078 |
| Mean | | 0.479 | 0.474 | 0.507 | 0.436 | 0.484 | 0.511 | 0.474 | 0.468 | 0.517 | 0.520 | 0.494 | 0.498 | 0.484 | 0.497 | 0.497 | 0.430 |  | 0.486 |  |  |
| Standard Deviation | | 0.025 | 0.016 | 0.028 | 0.013 | 0.008 | 0.024 | 0.034 | 0.011 | 0.030 | 0.024 | 0.064 | 0.038 | 0.030 | 0.020 | 0.022 | 0.027 |  |  |  |  |
| LOG_RQN | DEG thresholds | 0.001 | 0.002 | 0.003 | 0.004 | 0.005 | 0.006 | 0.007 | 0.008 | 0.009 | 0.010 | 0.020 | 0.030 | 0.050 | 0.070 | 0.100 | 1.000 | max | Mean | Standard Deviation | Coefficient of Variation |
| NDEG thresholds | gene numbers | 10124 | 10523 | 10756 | 10936 | 11079 | 11183 | 11282 | 11366 | 11447 | 11516 | 12001 | 12294 | 12690 | 12934 | 13236 | 15672 |  |  |  |  |
| 0.980 | 11 | 0.853 | 0.824 | 0.819 | 0.859 | 0.825 | 0.848 | 0.860 | 0.835 | 0.816 | 0.823 | 0.825 | 0.832 | 0.837 | 0.816 | 0.809 | 0.829 | 0.860 | 0.832 | 0.016 | 0.019 |
| 0.950 | 49 | 0.813 | 0.865 | 0.877 | 0.858 | 0.819 | 0.833 | 0.875 | 0.833 | 0.792 | 0.837 | 0.808 | 0.835 | 0.802 | 0.840 | 0.839 | 0.849 | 0.877 | 0.836 | 0.025 | 0.030 |
| 0.920 | 94 | 0.770 | 0.794 | 0.798 | 0.849 | 0.848 | 0.830 | 0.850 | 0.832 | 0.821 | 0.802 | 0.857 | 0.814 | 0.814 | 0.838 | 0.827 | 0.852 | 0.857 | 0.825 | 0.025 | 0.030 |
| 0.900 | 133 | 0.830 | 0.810 | 0.759 | 0.826 | 0.849 | 0.827 | 0.851 | 0.811 | 0.809 | 0.812 | 0.788 | 0.823 | 0.817 | 0.822 | 0.773 | 0.824 | 0.851 | 0.814 | 0.024 | 0.030 |
| 0.850 | 253 | 0.816 | 0.822 | 0.863 | 0.780 | 0.781 | 0.843 | 0.850 | 0.644 | 0.831 | 0.790 | 0.815 | 0.790 | 0.836 | 0.805 | 0.817 | 0.852 | 0.863 | 0.808 | 0.051 | 0.063 |
| Mean | | 0.816 | 0.823 | 0.823 | 0.834 | 0.824 | 0.836 | 0.857 | 0.791 | 0.814 | 0.813 | 0.819 | 0.819 | 0.821 | 0.824 | 0.813 | 0.841 |  | 0.823 |  |  |
| Standard Deviation | | 0.030 | 0.026 | 0.048 | 0.033 | 0.028 | 0.009 | 0.011 | 0.083 | 0.015 | 0.018 | 0.026 | 0.018 | 0.015 | 0.015 | 0.025 | 0.014 |  |  |  |  |
| LOG_RQN_Z | DEG thresholds | 0.001 | 0.002 | 0.003 | 0.004 | 0.005 | 0.006 | 0.007 | 0.008 | 0.009 | 0.010 | 0.020 | 0.030 | 0.050 | 0.070 | 0.100 | 1.000 | max | Mean | Standard Deviation | Coefficient of Variation |
| NDEG thresholds | gene numbers | 10124 | 10523 | 10756 | 10936 | 11079 | 11183 | 11282 | 11366 | 11447 | 11516 | 12001 | 12294 | 12690 | 12934 | 13236 | 15672 |  |  |  |  |
| 0.980 | 11 | 0.859 | 0.864 | 0.849 | 0.844 | 0.814 | 0.839 | 0.816 | 0.835 | 0.828 | 0.829 | 0.826 | 0.827 | 0.825 | 0.821 | 0.844 | 0.818 | 0.864 | 0.834 | 0.015 | 0.018 |
| 0.950 | 49 | 0.843 | 0.858 | 0.880 | 0.849 | 0.837 | 0.847 | 0.852 | 0.835 | 0.829 | 0.827 | 0.840 | 0.817 | 0.849 | 0.832 | 0.839 | 0.855 | 0.880 | 0.843 | 0.015 | 0.018 |
| 0.920 | 94 | 0.850 | 0.836 | 0.849 | 0.855 | 0.823 | 0.831 | 0.841 | 0.827 | 0.801 | 0.853 | 0.846 | 0.789 | 0.789 | 0.846 | 0.783 | 0.855 | 0.855 | 0.830 | 0.025 | 0.030 |
| 0.900 | 133 | 0.761 | 0.841 | 0.766 | 0.837 | 0.865 | 0.827 | 0.841 | 0.841 | 0.805 | 0.789 | 0.836 | 0.848 | 0.622 | 0.832 | 0.787 | 0.767 | 0.865 | 0.804 | 0.059 | 0.073 |
| 0.850 | 253 | 0.795 | 0.814 | 0.767 | 0.864 | 0.833 | 0.834 | 0.741 | 0.818 | 0.736 | 0.635 | 0.822 | 0.813 | 0.758 | 0.812 | 0.816 | 0.849 | 0.864 | 0.794 | 0.056 | 0.071 |
| Mean | | 0.822 | 0.842 | 0.822 | 0.850 | 0.834 | 0.835 | 0.818 | 0.831 | 0.800 | 0.787 | 0.834 | 0.819 | 0.769 | 0.829 | 0.814 | 0.829 |  | 0.821 |  |  |
| Standard Deviation | | 0.042 | 0.020 | 0.052 | 0.010 | 0.019 | 0.008 | 0.045 | 0.009 | 0.038 | 0.088 | 0.010 | 0.021 | 0.089 | 0.013 | 0.028 | 0.038 |  |  |  |  |
| LOG_NICG_Z | DEG thresholds | 0.001 | 0.002 | 0.003 | 0.004 | 0.005 | 0.006 | 0.007 | 0.008 | 0.009 | 0.010 | 0.020 | 0.030 | 0.050 | 0.070 | 0.100 | 1.000 | max | Mean | Standard Deviation | Coefficient of Variation |
| NDEG thresholds | gene numbers | 10124 | 10523 | 10756 | 10936 | 11079 | 11183 | 11282 | 11366 | 11447 | 11516 | 12001 | 12294 | 12690 | 12934 | 13236 | 15672 |  |  |  |  |
| 0.980 | 11 | 0.254 | 0.379 | 0.423 | 0.364 | 0.421 | 0.333 | 0.381 | 0.426 | 0.502 | 0.439 | 0.410 | 0.479 | 0.448 | 0.459 | 0.354 | 0.443 | 0.502 | 0.407 | 0.062 | 0.151 |
| 0.950 | 49 | 0.473 | 0.390 | 0.375 | 0.385 | 0.444 | 0.345 | 0.283 | 0.331 | 0.473 | 0.345 | 0.479 | 0.447 | 0.519 | 0.433 | 0.298 | 0.358 | 0.519 | 0.399 | 0.070 | 0.176 |
| 0.920 | 94 | 0.387 | 0.444 | 0.345 | 0.387 | 0.373 | 0.397 | 0.375 | 0.362 | 0.476 | 0.387 | 0.467 | 0.428 | 0.428 | 0.443 | 0.331 | 0.420 | 0.476 | 0.403 | 0.042 | 0.105 |
| 0.900 | 133 | 0.449 | 0.385 | 0.397 | 0.305 | 0.469 | 0.409 | 0.365 | 0.437 | 0.199 | 0.470 | 0.421 | 0.496 | 0.478 | 0.465 | 0.410 | 0.403 | 0.496 | 0.410 | 0.074 | 0.182 |
| 0.850 | 253 | 0.364 | 0.422 | 0.359 | 0.331 | 0.498 | 0.368 | 0.271 | 0.333 | 0.351 | 0.474 | 0.330 | 0.521 | 0.364 | 0.441 | 0.380 | 0.376 | 0.521 | 0.387 | 0.067 | 0.175 |
| Mean | | 0.386 | 0.404 | 0.380 | 0.355 | 0.441 | 0.370 | 0.335 | 0.378 | 0.400 | 0.423 | 0.421 | 0.474 | 0.448 | 0.448 | 0.355 | 0.400 |  | 0.401 |  |  |
| Standard Deviation | | 0.086 | 0.028 | 0.031 | 0.036 | 0.048 | 0.033 | 0.053 | 0.051 | 0.127 | 0.056 | 0.059 | 0.037 | 0.058 | 0.013 | 0.043 | 0.034 |  |  |  |  |
|  |  |  |  |  |  |  |  |  |  |  |  |  |  |  |  |  |  |  |  |  |  |
| MLP |  |  |  |  |  |  |  |  |  |  |  |  |  |  |  |  |  |  |  |  |  |
| LOG_NPN_Z | DEG thresholds | 0.001 | 0.002 | 0.003 | 0.004 | 0.005 | 0.006 | 0.007 | 0.008 | 0.009 | 0.010 | 0.020 | 0.030 | 0.050 | 0.070 | 0.100 | 1.000 | max | Mean | Standard Deviation | Coefficient of Variation |
| NDEG thresholds | gene numbers | 10124 | 10523 | 10756 | 10936 | 11079 | 11183 | 11282 | 11366 | 11447 | 11516 | 12001 | 12294 | 12690 | 12934 | 13236 | 15672 |  |  |  |  |
| 0.980 | 11 | 0.466 | 0.322 | 0.478 | 0.396 | 0.365 | 0.419 | 0.500 | 0.501 | 0.433 | 0.449 | 0.541 | 0.351 | 0.415 | 0.460 | 0.451 | 0.389 | 0.541 | 0.434 | 0.059 | 0.137 |
| 0.950 | 49 | 0.372 | 0.297 | 0.409 | 0.362 | 0.281 | 0.378 | 0.489 | 0.418 | 0.416 | 0.489 | 0.438 | 0.456 | 0.410 | 0.456 | 0.357 | 0.322 | 0.489 | 0.397 | 0.063 | 0.159 |
| 0.920 | 94 | 0.347 | 0.412 | 0.388 | 0.409 | 0.414 | 0.454 | 0.367 | 0.453 | 0.240 | 0.471 | 0.437 | 0.454 | 0.414 | 0.316 | 0.379 | 0.336 | 0.471 | 0.393 | 0.061 | 0.156 |
| 0.900 | 133 | 0.357 | 0.481 | 0.443 | 0.237 | 0.235 | 0.322 | 0.368 | 0.359 | 0.196 | 0.369 | 0.433 | 0.357 | 0.409 | 0.367 | 0.286 | 0.398 | 0.481 | 0.351 | 0.079 | 0.226 |
| 0.850 | 253 | 0.333 | 0.242 | 0.228 | 0.361 | 0.351 | 0.390 | 0.448 | 0.313 | 0.345 | 0.237 | 0.261 | 0.257 | 0.306 | 0.399 | 0.303 | 0.307 | 0.448 | 0.318 | 0.063 | 0.200 |
| Mean | | 0.375 | 0.351 | 0.389 | 0.353 | 0.329 | 0.393 | 0.434 | 0.409 | 0.326 | 0.403 | 0.422 | 0.375 | 0.391 | 0.400 | 0.355 | 0.351 |  | 0.378 |  |  |
| Standard Deviation | | 0.053 | 0.095 | 0.097 | 0.068 | 0.071 | 0.049 | 0.064 | 0.075 | 0.105 | 0.104 | 0.101 | 0.083 | 0.047 | 0.061 | 0.066 | 0.041 |  |  |  |  |
| LOG_RQN | DEG thresholds | 0.001 | 0.002 | 0.003 | 0.004 | 0.005 | 0.006 | 0.007 | 0.008 | 0.009 | 0.010 | 0.020 | 0.030 | 0.050 | 0.070 | 0.100 | 1.000 | max | Mean | Standard Deviation | Coefficient of Variation |
| NDEG thresholds | gene numbers | 10124 | 10523 | 10756 | 10936 | 11079 | 11183 | 11282 | 11366 | 11447 | 11516 | 12001 | 12294 | 12690 | 12934 | 13236 | 15672 |  |  |  |  |
| 0.980 | 11 | 0.834 | 0.827 | 0.839 | 0.852 | 0.833 | 0.845 | 0.831 | 0.809 | 0.827 | 0.845 | 0.818 | 0.825 | 0.835 | 0.850 | 0.807 | 0.838 | 0.852 | 0.832 | 0.013 | 0.016 |
| 0.950 | 49 | 0.821 | 0.863 | 0.854 | 0.834 | 0.841 | 0.831 | 0.867 | 0.799 | 0.807 | 0.815 | 0.837 | 0.833 | 0.832 | 0.830 | 0.816 | 0.843 | 0.867 | 0.833 | 0.019 | 0.023 |
| 0.920 | 94 | 0.827 | 0.792 | 0.837 | 0.821 | 0.837 | 0.832 | 0.872 | 0.849 | 0.821 | 0.804 | 0.857 | 0.796 | 0.808 | 0.858 | 0.823 | 0.842 | 0.872 | 0.830 | 0.023 | 0.028 |
| 0.900 | 133 | 0.822 | 0.825 | 0.883 | 0.840 | 0.849 | 0.834 | 0.815 | 0.791 | 0.822 | 0.803 | 0.823 | 0.838 | 0.835 | 0.847 | 0.789 | 0.856 | 0.883 | 0.830 | 0.024 | 0.029 |
| 0.850 | 253 | 0.821 | 0.844 | 0.835 | 0.852 | 0.826 | 0.830 | 0.828 | 0.786 | 0.818 | 0.825 | 0.813 | 0.815 | 0.822 | 0.851 | 0.821 | 0.822 | 0.852 | 0.826 | 0.016 | 0.019 |
| Mean | | 0.825 | 0.830 | 0.850 | 0.840 | 0.837 | 0.835 | 0.843 | 0.807 | 0.819 | 0.818 | 0.830 | 0.821 | 0.826 | 0.847 | 0.811 | 0.840 |  | 0.830 |  |  |
| Standard Deviation | | 0.006 | 0.026 | 0.020 | 0.013 | 0.009 | 0.006 | 0.025 | 0.025 | 0.007 | 0.017 | 0.018 | 0.017 | 0.012 | 0.010 | 0.014 | 0.012 |  |  |  |  |
| LOG_RQN_Z | DEG thresholds | 0.001 | 0.002 | 0.003 | 0.004 | 0.005 | 0.006 | 0.007 | 0.008 | 0.009 | 0.010 | 0.020 | 0.030 | 0.050 | 0.070 | 0.100 | 1.000 | max | Mean | Standard Deviation | Coefficient of Variation |
| NDEG thresholds | gene numbers | 10124 | 10523 | 10756 | 10936 | 11079 | 11183 | 11282 | 11366 | 11447 | 11516 | 12001 | 12294 | 12690 | 12934 | 13236 | 15672 |  |  |  |  |
| 0.980 | 11 | 0.849 | 0.850 | 0.834 | 0.802 | 0.820 | 0.817 | 0.803 | 0.812 | 0.828 | 0.849 | 0.827 | 0.822 | 0.827 | 0.821 | 0.836 | 0.849 | 0.850 | 0.828 | 0.016 | 0.019 |
| 0.950 | 49 | 0.807 | 0.810 | 0.871 | 0.833 | 0.830 | 0.839 | 0.845 | 0.809 | 0.836 | 0.836 | 0.848 | 0.856 | 0.845 | 0.838 | 0.807 | 0.851 | 0.871 | 0.835 | 0.019 | 0.022 |
| 0.920 | 94 | 0.854 | 0.848 | 0.855 | 0.829 | 0.846 | 0.825 | 0.831 | 0.823 | 0.799 | 0.859 | 0.841 | 0.787 | 0.816 | 0.849 | 0.803 | 0.823 | 0.859 | 0.830 | 0.021 | 0.026 |
| 0.900 | 133 | 0.805 | 0.816 | 0.836 | 0.836 | 0.836 | 0.830 | 0.838 | 0.803 | 0.810 | 0.836 | 0.845 | 0.841 | 0.802 | 0.842 | 0.791 | 0.814 | 0.845 | 0.824 | 0.018 | 0.021 |
| 0.850 | 253 | 0.832 | 0.840 | 0.838 | 0.838 | 0.837 | 0.819 | 0.844 | 0.808 | 0.804 | 0.833 | 0.829 | 0.825 | 0.824 | 0.875 | 0.793 | 0.821 | 0.875 | 0.829 | 0.019 | 0.023 |
| Mean | | 0.829 | 0.832 | 0.847 | 0.827 | 0.834 | 0.826 | 0.832 | 0.811 | 0.815 | 0.843 | 0.838 | 0.826 | 0.823 | 0.845 | 0.806 | 0.831 |  | 0.829 |  |  |
| Standard Deviation | | 0.023 | 0.019 | 0.016 | 0.015 | 0.009 | 0.009 | 0.018 | 0.008 | 0.016 | 0.011 | 0.010 | 0.026 | 0.016 | 0.019 | 0.018 | 0.017 |  |  |  |  |
| LOG_NICG_Z | DEG thresholds | 0.001 | 0.002 | 0.003 | 0.004 | 0.005 | 0.006 | 0.007 | 0.008 | 0.009 | 0.010 | 0.020 | 0.030 | 0.050 | 0.070 | 0.100 | 1.000 | max | Mean | Standard Deviation | Coefficient of Variation |
| NDEG thresholds | gene numbers | 10124 | 10523 | 10756 | 10936 | 11079 | 11183 | 11282 | 11366 | 11447 | 11516 | 12001 | 12294 | 12690 | 12934 | 13236 | 15672 |  |  |  |  |
| 0.980 | 11 | 0.283 | 0.412 | 0.437 | 0.386 | 0.428 | 0.366 | 0.266 | 0.301 | 0.475 | 0.416 | 0.508 | 0.451 | 0.435 | 0.473 | 0.428 | 0.311 | 0.508 | 0.398 | 0.073 | 0.184 |
| 0.950 | 49 | 0.489 | 0.232 | 0.309 | 0.445 | 0.435 | 0.220 | 0.249 | 0.202 | 0.469 | 0.401 | 0.378 | 0.432 | 0.446 | 0.386 | 0.288 | 0.315 | 0.489 | 0.356 | 0.096 | 0.271 |
| 0.920 | 94 | 0.235 | 0.214 | 0.408 | 0.201 | 0.386 | 0.218 | 0.307 | 0.403 | 0.435 | 0.245 | 0.348 | 0.414 | 0.381 | 0.313 | 0.221 | 0.293 | 0.435 | 0.314 | 0.083 | 0.266 |
| 0.900 | 133 | 0.278 | 0.302 | 0.376 | 0.295 | 0.412 | 0.421 | 0.399 | 0.398 | 0.462 | 0.209 | 0.320 | 0.437 | 0.442 | 0.488 | 0.360 | 0.150 | 0.488 | 0.359 | 0.094 | 0.261 |
| 0.850 | 253 | 0.283 | 0.349 | 0.400 | 0.318 | 0.297 | 0.297 | 0.262 | 0.416 | 0.305 | 0.438 | 0.299 | 0.408 | 0.397 | 0.453 | 0.215 | 0.385 | 0.453 | 0.345 | 0.070 | 0.203 |
| Mean | | 0.314 | 0.302 | 0.386 | 0.329 | 0.391 | 0.304 | 0.297 | 0.344 | 0.429 | 0.342 | 0.371 | 0.428 | 0.420 | 0.423 | 0.302 | 0.291 |  | 0.355 |  |  |
| Standard Deviation | | 0.100 | 0.082 | 0.048 | 0.093 | 0.056 | 0.089 | 0.061 | 0.092 | 0.071 | 0.106 | 0.083 | 0.018 | 0.029 | 0.073 | 0.092 | 0.086 |  |  |  |  |
|  |  |  |  |  |  |  |  |  |  |  |  |  |  |  |  |  |  |  |  |  |  |
| XGB |  |  |  |  |  |  |  |  |  |  |  |  |  |  |  |  |  |  |  |  |  |
| LOG_NPN_Z | DEG thresholds | 0.001 | 0.002 | 0.003 | 0.004 | 0.005 | 0.006 | 0.007 | 0.008 | 0.009 | 0.010 | 0.020 | 0.030 | 0.050 | 0.070 | 0.100 | 1.000 | max | Mean | Standard Deviation | Coefficient of Variation |
| NDEG thresholds | gene numbers | 10124 | 10523 | 10756 | 10936 | 11079 | 11183 | 11282 | 11366 | 11447 | 11516 | 12001 | 12294 | 12690 | 12934 | 13236 | 15672 |  |  |  |  |
| 0.980 | 11 | 0.375 | 0.264 | 0.317 | 0.291 | 0.325 | 0.245 | 0.446 | 0.295 | 0.228 | 0.412 | 0.216 | 0.290 | 0.286 | 0.322 | 0.237 | 0.355 | 0.446 | 0.307 | 0.066 | 0.214 |
| 0.950 | 49 | 0.393 | 0.268 | 0.135 | 0.284 | 0.275 | 0.339 | 0.319 | 0.362 | 0.395 | 0.327 | 0.186 | 0.342 | 0.306 | 0.308 | 0.229 | 0.419 | 0.419 | 0.305 | 0.076 | 0.249 |
| 0.920 | 94 | 0.168 | 0.359 | 0.304 | 0.306 | 0.258 | 0.416 | 0.382 | 0.338 | 0.352 | 0.378 | 0.217 | 0.321 | 0.439 | 0.433 | 0.215 | 0.428 | 0.439 | 0.332 | 0.083 | 0.251 |
| 0.900 | 133 | 0.416 | 0.382 | 0.131 | 0.351 | 0.340 | 0.439 | 0.363 | 0.297 | 0.465 | 0.355 | 0.263 | 0.289 | 0.399 | 0.373 | 0.253 | 0.387 | 0.465 | 0.344 | 0.083 | 0.240 |
| 0.850 | 253 | 0.238 | 0.290 | 0.114 | 0.188 | 0.356 | 0.340 | 0.341 | 0.423 | 0.386 | 0.383 | 0.171 | 0.273 | 0.283 | 0.377 | 0.302 | 0.310 | 0.423 | 0.298 | 0.086 | 0.288 |
| Mean | | 0.318 | 0.312 | 0.200 | 0.284 | 0.311 | 0.356 | 0.370 | 0.343 | 0.365 | 0.371 | 0.211 | 0.303 | 0.342 | 0.363 | 0.247 | 0.380 |  | 0.317 |  |  |
| Standard Deviation | | 0.109 | 0.054 | 0.101 | 0.060 | 0.042 | 0.077 | 0.049 | 0.053 | 0.087 | 0.032 | 0.035 | 0.028 | 0.072 | 0.050 | 0.033 | 0.048 |  |  |  |  |
| LOG_RQN | DEG thresholds | 0.001 | 0.002 | 0.003 | 0.004 | 0.005 | 0.006 | 0.007 | 0.008 | 0.009 | 0.010 | 0.020 | 0.030 | 0.050 | 0.070 | 0.100 | 1.000 | max | Mean | Standard Deviation | Coefficient of Variation |
| NDEG thresholds | gene numbers | 10124 | 10523 | 10756 | 10936 | 11079 | 11183 | 11282 | 11366 | 11447 | 11516 | 12001 | 12294 | 12690 | 12934 | 13236 | 15672 |  |  |  |  |
| 0.980 | 11 | 0.716 | 0.674 | 0.753 | 0.767 | 0.679 | 0.738 | 0.758 | 0.677 | 0.676 | 0.702 | 0.587 | 0.715 | 0.724 | 0.763 | 0.681 | 0.677 | 0.767 | 0.705 | 0.047 | 0.066 |
| 0.950 | 49 | 0.691 | 0.703 | 0.735 | 0.688 | 0.631 | 0.745 | 0.714 | 0.726 | 0.561 | 0.637 | 0.692 | 0.713 | 0.592 | 0.713 | 0.658 | 0.734 | 0.745 | 0.683 | 0.053 | 0.078 |
| 0.920 | 94 | 0.358 | 0.566 | 0.647 | 0.541 | 0.544 | 0.733 | 0.663 | 0.601 | 0.339 | 0.364 | 0.613 | 0.545 | 0.430 | 0.465 | 0.488 | 0.675 | 0.733 | 0.536 | 0.120 | 0.224 |
| 0.900 | 133 | 0.358 | 0.463 | 0.400 | 0.433 | 0.609 | 0.634 | 0.696 | 0.636 | 0.658 | 0.640 | 0.555 | 0.645 | 0.326 | 0.479 | 0.467 | 0.417 | 0.696 | 0.526 | 0.121 | 0.230 |
| 0.850 | 253 | 0.469 | 0.321 | 0.375 | 0.561 | 0.167 | 0.263 | 0.425 | 0.344 | 0.627 | 0.225 | 0.286 | 0.381 | 0.245 | 0.423 | 0.318 | 0.102 | 0.627 | 0.346 | 0.137 | 0.397 |
| Mean | | 0.518 | 0.545 | 0.582 | 0.598 | 0.526 | 0.622 | 0.651 | 0.597 | 0.572 | 0.514 | 0.547 | 0.600 | 0.463 | 0.569 | 0.522 | 0.521 |  | 0.559 |  |  |
| Standard Deviation | | 0.175 | 0.157 | 0.182 | 0.131 | 0.206 | 0.206 | 0.131 | 0.149 | 0.138 | 0.208 | 0.154 | 0.141 | 0.195 | 0.157 | 0.150 | 0.264 |  |  |  |  |
| LOG_RQN_Z | DEG thresholds | 0.001 | 0.002 | 0.003 | 0.004 | 0.005 | 0.006 | 0.007 | 0.008 | 0.009 | 0.010 | 0.020 | 0.030 | 0.050 | 0.070 | 0.100 | 1.000 | max | Mean | Standard Deviation | Coefficient of Variation |
| NDEG thresholds | gene numbers | 10124 | 10523 | 10756 | 10936 | 11079 | 11183 | 11282 | 11366 | 11447 | 11516 | 12001 | 12294 | 12690 | 12934 | 13236 | 15672 |  |  |  |  |
| 0.980 | 11 | 0.782 | 0.794 | 0.787 | 0.763 | 0.734 | 0.747 | 0.771 | 0.673 | 0.658 | 0.685 | 0.629 | 0.687 | 0.705 | 0.786 | 0.665 | 0.724 | 0.794 | 0.724 | 0.054 | 0.074 |
| 0.950 | 49 | 0.651 | 0.746 | 0.764 | 0.680 | 0.698 | 0.745 | 0.745 | 0.693 | 0.630 | 0.600 | 0.704 | 0.745 | 0.566 | 0.706 | 0.690 | 0.747 | 0.764 | 0.694 | 0.058 | 0.083 |
| 0.920 | 94 | 0.581 | 0.656 | 0.656 | 0.584 | 0.581 | 0.687 | 0.702 | 0.682 | 0.369 | 0.483 | 0.599 | 0.584 | 0.394 | 0.399 | 0.521 | 0.649 | 0.702 | 0.571 | 0.108 | 0.190 |
| 0.900 | 133 | 0.428 | 0.567 | 0.518 | 0.473 | 0.581 | 0.645 | 0.708 | 0.627 | 0.625 | 0.589 | 0.549 | 0.646 | 0.368 | 0.456 | 0.423 | 0.462 | 0.708 | 0.542 | 0.098 | 0.181 |
| 0.850 | 253 | 0.345 | 0.477 | 0.401 | 0.379 | 0.239 | 0.228 | 0.439 | 0.244 | 0.664 | 0.348 | 0.353 | 0.396 | 0.162 | 0.440 | 0.390 | 0.094 | 0.664 | 0.350 | 0.136 | 0.388 |
| Mean | | 0.557 | 0.648 | 0.625 | 0.576 | 0.567 | 0.610 | 0.673 | 0.584 | 0.589 | 0.541 | 0.567 | 0.612 | 0.439 | 0.557 | 0.538 | 0.535 |  | 0.576 |  |  |
| Standard Deviation | | 0.175 | 0.129 | 0.165 | 0.154 | 0.196 | 0.218 | 0.134 | 0.192 | 0.124 | 0.129 | 0.132 | 0.134 | 0.207 | 0.176 | 0.137 | 0.271 |  |  |  |  |
| LOG_NICG_Z | DEG thresholds | 0.001 | 0.002 | 0.003 | 0.004 | 0.005 | 0.006 | 0.007 | 0.008 | 0.009 | 0.010 | 0.020 | 0.030 | 0.050 | 0.070 | 0.100 | 1.000 | max | Mean | Standard Deviation | Coefficient of Variation |
| NDEG thresholds | gene numbers | 10124 | 10523 | 10756 | 10936 | 11079 | 11183 | 11282 | 11366 | 11447 | 11516 | 12001 | 12294 | 12690 | 12934 | 13236 | 15672 |  |  |  |  |
| 0.980 | 11 | 0.240 | 0.440 | 0.244 | 0.478 | 0.284 | 0.289 | 0.358 | 0.312 | 0.256 | 0.381 | 0.374 | 0.292 | 0.244 | 0.279 | 0.358 | 0.386 | 0.478 | 0.326 | 0.073 | 0.224 |
| 0.950 | 49 | 0.296 | 0.178 | 0.363 | 0.238 | 0.178 | 0.265 | 0.350 | 0.237 | 0.264 | 0.260 | 0.331 | 0.364 | 0.365 | 0.265 | 0.331 | 0.298 | 0.365 | 0.286 | 0.062 | 0.215 |
| 0.920 | 94 | 0.301 | 0.191 | 0.413 | 0.428 | 0.246 | 0.310 | 0.364 | 0.266 | 0.274 | 0.386 | 0.332 | 0.275 | 0.360 | 0.277 | 0.230 | 0.332 | 0.428 | 0.312 | 0.067 | 0.214 |
| 0.900 | 133 | 0.150 | 0.364 | 0.263 | 0.423 | 0.305 | 0.303 | 0.352 | 0.308 | 0.279 | 0.332 | 0.305 | 0.335 | 0.266 | 0.147 | 0.132 | 0.244 | 0.423 | 0.282 | 0.081 | 0.289 |
| 0.850 | 253 | 0.198 | 0.238 | 0.280 | 0.135 | 0.342 | 0.128 | 0.293 | 0.212 | 0.330 | 0.318 | 0.182 | 0.167 | 0.212 | 0.264 | 0.327 | 0.278 | 0.342 | 0.244 | 0.070 | 0.287 |
| Mean | | 0.237 | 0.282 | 0.313 | 0.340 | 0.271 | 0.259 | 0.343 | 0.267 | 0.281 | 0.335 | 0.305 | 0.287 | 0.289 | 0.247 | 0.276 | 0.308 |  | 0.290 |  |  |
| Standard Deviation | | 0.064 | 0.115 | 0.072 | 0.147 | 0.062 | 0.075 | 0.029 | 0.044 | 0.029 | 0.052 | 0.073 | 0.076 | 0.069 | 0.056 | 0.094 | 0.054 |  |  |  |  |
