# Supplementary material for: Normalization and Selecting Non-Differentially Expressed Genes Improve Machine Learning Modelling of Cross-Platform Transcriptomic Data
Source: Trans Artif Intell. Author manuscript; Available in PMC 2025 Jul 8. (PMC12235674; doi:10.53941/tai.2025.100005)
Supplement: Supplementary [file NIHMS2087281-supplement-Supplementary.zip › Supplementary table 5.docx]

| Supplementary table 5. Average classification performance results (**Kappa**) on data constructed using DEG and NDEG genes selected via one-way ANOVA. (**Model-A**) | | | | | | | | | | | | | | | | | | | | | |
| --- | --- | --- | --- | --- | --- | --- | --- | --- | --- | --- | --- | --- | --- | --- | --- | --- | --- | --- | --- | --- | --- |
| SVM | |  |  |  |  |  |  |  |  |  |  |  |  |  |  |  |  |  |  |  |  |
| LOG_NPN_Z | DEG thresholds | 0.001 | 0.002 | 0.003 | 0.004 | 0.005 | 0.006 | 0.007 | 0.008 | 0.009 | 0.010 | 0.020 | 0.030 | 0.050 | 0.070 | 0.100 | 1.000 | max | Mean | Standard Deviation | Coefficient of Variation |
| NDEG thresholds | gene numbers | 11408 | 11790 | 12019 | 12189 | 12303 | 12427 | 12548 | 12621 | 12690 | 12757 | 13155 | 13409 | 13744 | 13955 | 14197 | 15672 |  |  |  |  |
| 0.980 | 6 | 0.269 | 0.285 | 0.268 | 0.263 | 0.291 | 0.232 | 0.245 | 0.278 | 0.243 | 0.253 | 0.260 | 0.256 | 0.251 | 0.277 | 0.256 | 0.283 | 0.291 | 0.263 | 0.017 | 0.064 |
| 0.950 | 22 | 0.263 | 0.256 | 0.273 | 0.261 | 0.268 | 0.242 | 0.234 | 0.256 | 0.247 | 0.231 | 0.308 | 0.246 | 0.255 | 0.259 | 0.249 | 0.260 | 0.308 | 0.257 | 0.018 | 0.069 |
| 0.920 | 39 | 0.269 | 0.262 | 0.272 | 0.279 | 0.280 | 0.259 | 0.233 | 0.288 | 0.227 | 0.240 | 0.306 | 0.233 | 0.252 | 0.244 | 0.233 | 0.255 | 0.306 | 0.258 | 0.023 | 0.089 |
| 0.900 | 58 | 0.277 | 0.238 | 0.269 | 0.256 | 0.260 | 0.258 | 0.249 | 0.272 | 0.227 | 0.282 | 0.236 | 0.242 | 0.255 | 0.242 | 0.234 | 0.254 | 0.282 | 0.253 | 0.016 | 0.064 |
| 0.850 | 97 | 0.276 | 0.266 | 0.273 | 0.259 | 0.281 | 0.251 | 0.246 | 0.244 | 0.243 | 0.249 | 0.243 | 0.239 | 0.239 | 0.266 | 0.262 | 0.256 | 0.281 | 0.256 | 0.014 | 0.053 |
| Mean | | 0.271 | 0.262 | 0.271 | 0.263 | 0.276 | 0.248 | 0.241 | 0.268 | 0.238 | 0.251 | 0.271 | 0.243 | 0.250 | 0.258 | 0.247 | 0.262 |  | 0.257 |  |  |
| Standard Deviation | | 0.006 | 0.017 | 0.002 | 0.009 | 0.012 | 0.011 | 0.007 | 0.018 | 0.010 | 0.020 | 0.034 | 0.009 | 0.006 | 0.015 | 0.013 | 0.012 |  |  |  |  |
| LOG-RQN | DEG thresholds | 0.001 | 0.002 | 0.003 | 0.004 | 0.005 | 0.006 | 0.007 | 0.008 | 0.009 | 0.010 | 0.020 | 0.030 | 0.050 | 0.070 | 0.100 | 1.000 | max | Mean | Standard Deviation | Coefficient of Variation |
| NDEG thresholds | gene numbers | 11408 | 11790 | 12019 | 12189 | 12303 | 12427 | 12548 | 12621 | 12690 | 12757 | 13155 | 13409 | 13744 | 13955 | 14197 | 15672 |  |  |  |  |
| 0.980 | 6 | 0.514 | 0.577 | 0.534 | 0.560 | 0.496 | 0.504 | 0.545 | 0.581 | 0.490 | 0.495 | 0.444 | 0.479 | 0.472 | 0.521 | 0.453 | 0.497 | 0.581 | 0.510 | 0.041 | 0.080 |
| 0.950 | 22 | 0.393 | 0.494 | 0.410 | 0.418 | 0.438 | 0.334 | 0.433 | 0.463 | 0.345 | 0.340 | 0.416 | 0.263 | 0.276 | 0.290 | 0.294 | 0.185 | 0.494 | 0.362 | 0.085 | 0.234 |
| 0.920 | 39 | 0.282 | 0.270 | 0.311 | 0.281 | 0.310 | 0.277 | 0.284 | 0.273 | 0.295 | 0.272 | 0.340 | 0.239 | 0.268 | 0.254 | 0.269 | 0.160 | 0.340 | 0.274 | 0.039 | 0.141 |
| 0.900 | 58 | 0.451 | 0.463 | 0.400 | 0.372 | 0.364 | 0.348 | 0.406 | 0.474 | 0.299 | 0.366 | 0.365 | 0.330 | 0.316 | 0.391 | 0.356 | 0.191 | 0.474 | 0.368 | 0.069 | 0.187 |
| 0.850 | 97 | 0.474 | 0.504 | 0.375 | 0.408 | 0.369 | 0.430 | 0.444 | 0.429 | 0.369 | 0.456 | 0.378 | 0.394 | 0.362 | 0.364 | 0.426 | 0.218 | 0.504 | 0.400 | 0.065 | 0.162 |
| Mean | | 0.423 | 0.462 | 0.406 | 0.408 | 0.396 | 0.379 | 0.422 | 0.444 | 0.360 | 0.386 | 0.388 | 0.341 | 0.339 | 0.364 | 0.359 | 0.250 |  | 0.383 |  |  |
| Standard Deviation | | 0.090 | 0.115 | 0.081 | 0.101 | 0.073 | 0.089 | 0.094 | 0.111 | 0.079 | 0.090 | 0.041 | 0.098 | 0.083 | 0.104 | 0.080 | 0.139 |  |  |  |  |
| LOG-RQN-Z | DEG thresholds | 0.001 | 0.002 | 0.003 | 0.004 | 0.005 | 0.006 | 0.007 | 0.008 | 0.009 | 0.010 | 0.020 | 0.030 | 0.050 | 0.070 | 0.100 | 1.000 | max | Mean | Standard Deviation | Coefficient of Variation |
| NDEG thresholds | gene numbers | 11408 | 11790 | 12019 | 12189 | 12303 | 12427 | 12548 | 12621 | 12690 | 12757 | 13155 | 13409 | 13744 | 13955 | 14197 | 15672 |  |  |  |  |
| 0.980 | 6 | 0.514 | 0.555 | 0.528 | 0.550 | 0.521 | 0.496 | 0.549 | 0.594 | 0.487 | 0.485 | 0.454 | 0.445 | 0.486 | 0.498 | 0.458 | 0.498 | 0.594 | 0.507 | 0.041 | 0.080 |
| 0.950 | 22 | 0.414 | 0.493 | 0.402 | 0.407 | 0.429 | 0.347 | 0.434 | 0.462 | 0.369 | 0.320 | 0.421 | 0.256 | 0.269 | 0.308 | 0.278 | 0.184 | 0.493 | 0.362 | 0.086 | 0.237 |
| 0.920 | 39 | 0.285 | 0.289 | 0.310 | 0.280 | 0.310 | 0.289 | 0.286 | 0.273 | 0.295 | 0.266 | 0.322 | 0.234 | 0.262 | 0.252 | 0.274 | 0.155 | 0.322 | 0.274 | 0.039 | 0.142 |
| 0.900 | 58 | 0.419 | 0.464 | 0.376 | 0.375 | 0.372 | 0.350 | 0.401 | 0.480 | 0.310 | 0.365 | 0.350 | 0.325 | 0.304 | 0.366 | 0.325 | 0.182 | 0.480 | 0.360 | 0.069 | 0.191 |
| 0.850 | 97 | 0.467 | 0.471 | 0.378 | 0.393 | 0.343 | 0.424 | 0.444 | 0.425 | 0.354 | 0.437 | 0.389 | 0.397 | 0.342 | 0.340 | 0.326 | 0.220 | 0.471 | 0.384 | 0.064 | 0.166 |
| Mean | | 0.420 | 0.454 | 0.399 | 0.401 | 0.395 | 0.381 | 0.423 | 0.447 | 0.363 | 0.374 | 0.387 | 0.331 | 0.332 | 0.353 | 0.332 | 0.248 |  | 0.378 |  |  |
| Standard Deviation | | 0.086 | 0.099 | 0.080 | 0.097 | 0.083 | 0.080 | 0.094 | 0.116 | 0.076 | 0.088 | 0.053 | 0.090 | 0.091 | 0.092 | 0.075 | 0.142 |  |  |  |  |
| LOG-NICG-Z | DEG thresholds | 0.001 | 0.002 | 0.003 | 0.004 | 0.005 | 0.006 | 0.007 | 0.008 | 0.009 | 0.010 | 0.020 | 0.030 | 0.050 | 0.070 | 0.100 | 1.000 | max | Mean | Standard Deviation | Coefficient of Variation |
| NDEG thresholds | gene numbers | 11408 | 11790 | 12019 | 12189 | 12303 | 12427 | 12548 | 12621 | 12690 | 12757 | 13155 | 13409 | 13744 | 13955 | 14197 | 15672 |  |  |  |  |
| 0.980 | 6 | 0.265 | 0.245 | 0.251 | 0.291 | 0.249 | 0.240 | 0.248 | 0.243 | 0.248 | 0.263 | 0.246 | 0.251 | 0.236 | 0.239 | 0.246 | 0.257 | 0.291 | 0.251 | 0.013 | 0.053 |
| 0.950 | 22 | 0.270 | 0.252 | 0.250 | 0.274 | 0.247 | 0.222 | 0.253 | 0.257 | 0.241 | 0.238 | 0.337 | 0.239 | 0.243 | 0.288 | 0.250 | 0.253 | 0.337 | 0.257 | 0.026 | 0.102 |
| 0.920 | 39 | 0.255 | 0.251 | 0.245 | 0.244 | 0.248 | 0.242 | 0.250 | 0.241 | 0.243 | 0.246 | 0.242 | 0.248 | 0.241 | 0.238 | 0.230 | 0.258 | 0.258 | 0.245 | 0.007 | 0.027 |
| 0.900 | 58 | 0.281 | 0.262 | 0.265 | 0.258 | 0.251 | 0.247 | 0.259 | 0.272 | 0.238 | 0.241 | 0.239 | 0.262 | 0.244 | 0.238 | 0.245 | 0.269 | 0.281 | 0.254 | 0.014 | 0.053 |
| 0.850 | 97 | 0.253 | 0.251 | 0.245 | 0.247 | 0.266 | 0.238 | 0.259 | 0.242 | 0.242 | 0.259 | 0.238 | 0.255 | 0.258 | 0.237 | 0.244 | 0.256 | 0.266 | 0.250 | 0.009 | 0.036 |
| Mean | | 0.265 | 0.252 | 0.251 | 0.263 | 0.252 | 0.238 | 0.254 | 0.251 | 0.242 | 0.249 | 0.260 | 0.251 | 0.245 | 0.248 | 0.243 | 0.258 |  | 0.251 |  |  |
| Standard Deviation | | 0.011 | 0.006 | 0.008 | 0.020 | 0.008 | 0.010 | 0.005 | 0.014 | 0.004 | 0.011 | 0.043 | 0.008 | 0.008 | 0.022 | 0.008 | 0.006 |  |  |  |  |
|  |  |  |  |  |  |  |  |  |  |  |  |  |  |  |  |  |  |  |  |  |  |
| RF | |  |  |  |  |  |  |  |  |  |  |  |  |  |  |  |  |  |  |  |  |
| LOG_NPN_Z | DEG thresholds | 0.001 | 0.002 | 0.003 | 0.004 | 0.005 | 0.006 | 0.007 | 0.008 | 0.009 | 0.010 | 0.020 | 0.030 | 0.050 | 0.070 | 0.100 | 1.000 | max | Mean | Standard Deviation | Coefficient of Variation |
| NDEG thresholds | gene numbers | 11408 | 11790 | 12019 | 12189 | 12303 | 12427 | 12548 | 12621 | 12690 | 12757 | 13155 | 13409 | 13744 | 13955 | 14197 | 15672 |  |  |  |  |
| 0.980 | 6 | 0.251 | 0.289 | 0.198 | 0.307 | 0.288 | 0.394 | 0.313 | 0.325 | 0.176 | 0.323 | 0.216 | 0.266 | 0.260 | 0.330 | 0.277 | 0.338 | 0.394 | 0.285 | 0.056 | 0.198 |
| 0.950 | 22 | 0.226 | 0.293 | 0.214 | 0.348 | 0.255 | 0.200 | 0.275 | 0.322 | 0.216 | 0.149 | 0.239 | 0.194 | 0.221 | 0.171 | 0.187 | 0.161 | 0.348 | 0.229 | 0.057 | 0.248 |
| 0.920 | 39 | 0.218 | 0.267 | 0.164 | 0.201 | 0.217 | 0.275 | 0.131 | 0.213 | 0.207 | 0.198 | 0.172 | 0.321 | 0.194 | 0.206 | 0.281 | 0.144 | 0.321 | 0.213 | 0.051 | 0.241 |
| 0.900 | 58 | 0.105 | 0.192 | 0.144 | 0.302 | 0.300 | 0.299 | 0.222 | 0.198 | 0.143 | 0.156 | 0.166 | 0.251 | 0.171 | 0.152 | 0.243 | 0.127 | 0.302 | 0.198 | 0.064 | 0.324 |
| 0.850 | 97 | 0.126 | 0.153 | 0.211 | 0.284 | 0.174 | 0.272 | 0.098 | 0.283 | 0.144 | 0.128 | 0.266 | 0.352 | 0.056 | 0.186 | 0.223 | 0.240 | 0.352 | 0.200 | 0.080 | 0.402 |
| Mean | | 0.185 | 0.239 | 0.186 | 0.289 | 0.247 | 0.288 | 0.208 | 0.268 | 0.177 | 0.191 | 0.212 | 0.277 | 0.180 | 0.209 | 0.242 | 0.202 |  | 0.225 |  |  |
| Standard Deviation | | 0.065 | 0.063 | 0.031 | 0.054 | 0.052 | 0.070 | 0.092 | 0.060 | 0.034 | 0.078 | 0.043 | 0.062 | 0.077 | 0.071 | 0.039 | 0.088 |  |  |  |  |
| LOG-RQN | DEG thresholds | 0.001 | 0.002 | 0.003 | 0.004 | 0.005 | 0.006 | 0.007 | 0.008 | 0.009 | 0.010 | 0.020 | 0.030 | 0.050 | 0.070 | 0.100 | 1.000 | max | Mean | Standard Deviation | Coefficient of Variation |
| NDEG thresholds | gene numbers | 11408 | 11790 | 12019 | 12189 | 12303 | 12427 | 12548 | 12621 | 12690 | 12757 | 13155 | 13409 | 13744 | 13955 | 14197 | 15672 |  |  |  |  |
| 0.980 | 6 | 0.425 | 0.398 | 0.395 | 0.288 | 0.347 | 0.312 | 0.380 | 0.355 | 0.360 | 0.298 | 0.373 | 0.400 | 0.336 | 0.415 | 0.423 | 0.401 | 0.425 | 0.369 | 0.044 | 0.118 |
| 0.950 | 22 | 0.209 | 0.277 | 0.258 | 0.211 | 0.234 | 0.166 | 0.161 | 0.284 | 0.205 | 0.226 | 0.230 | 0.136 | 0.112 | 0.155 | 0.110 | 0.037 | 0.284 | 0.188 | 0.067 | 0.359 |
| 0.920 | 39 | 0.181 | 0.128 | 0.225 | 0.192 | 0.203 | 0.142 | 0.172 | 0.126 | 0.158 | 0.134 | 0.148 | 0.088 | 0.110 | 0.108 | 0.109 | 0.017 | 0.225 | 0.140 | 0.050 | 0.359 |
| 0.900 | 58 | 0.250 | 0.284 | 0.239 | 0.197 | 0.203 | 0.256 | 0.249 | 0.325 | 0.150 | 0.169 | 0.203 | 0.159 | 0.158 | 0.268 | 0.205 | 0.061 | 0.325 | 0.211 | 0.064 | 0.303 |
| 0.850 | 97 | 0.363 | 0.348 | 0.248 | 0.288 | 0.309 | 0.228 | 0.343 | 0.372 | 0.238 | 0.269 | 0.250 | 0.206 | 0.164 | 0.285 | 0.359 | 0.097 | 0.372 | 0.273 | 0.077 | 0.282 |
| Mean | | 0.285 | 0.287 | 0.273 | 0.235 | 0.259 | 0.221 | 0.261 | 0.292 | 0.222 | 0.219 | 0.241 | 0.198 | 0.176 | 0.247 | 0.241 | 0.123 |  | 0.236 |  |  |
| Standard Deviation | | 0.104 | 0.102 | 0.069 | 0.049 | 0.066 | 0.069 | 0.099 | 0.099 | 0.085 | 0.068 | 0.083 | 0.121 | 0.093 | 0.120 | 0.144 | 0.158 |  |  |  |  |
| LOG-RQN-Z | DEG thresholds | 0.001 | 0.002 | 0.003 | 0.004 | 0.005 | 0.006 | 0.007 | 0.008 | 0.009 | 0.010 | 0.020 | 0.030 | 0.050 | 0.070 | 0.100 | 1.000 | max | Mean | Standard Deviation | Coefficient of Variation |
| NDEG thresholds | gene numbers | 11408 | 11790 | 12019 | 12189 | 12303 | 12427 | 12548 | 12621 | 12690 | 12757 | 13155 | 13409 | 13744 | 13955 | 14197 | 15672 |  |  |  |  |
| 0.980 | 6 | 0.384 | 0.361 | 0.399 | 0.294 | 0.338 | 0.385 | 0.431 | 0.395 | 0.399 | 0.321 | 0.349 | 0.364 | 0.432 | 0.339 | 0.388 | 0.325 | 0.432 | 0.369 | 0.039 | 0.107 |
| 0.950 | 22 | 0.235 | 0.261 | 0.266 | 0.148 | 0.242 | 0.131 | 0.202 | 0.337 | 0.252 | 0.204 | 0.199 | 0.166 | 0.157 | 0.186 | 0.143 | 0.032 | 0.337 | 0.198 | 0.071 | 0.357 |
| 0.920 | 39 | 0.155 | 0.199 | 0.185 | 0.148 | 0.152 | 0.120 | 0.138 | 0.141 | 0.109 | 0.165 | 0.229 | 0.118 | 0.128 | 0.156 | 0.162 | 0.028 | 0.229 | 0.146 | 0.044 | 0.302 |
| 0.900 | 58 | 0.242 | 0.274 | 0.198 | 0.191 | 0.200 | 0.229 | 0.196 | 0.321 | 0.139 | 0.215 | 0.179 | 0.160 | 0.134 | 0.186 | 0.169 | 0.025 | 0.321 | 0.191 | 0.065 | 0.341 |
| 0.850 | 97 | 0.357 | 0.379 | 0.206 | 0.190 | 0.295 | 0.304 | 0.272 | 0.312 | 0.254 | 0.318 | 0.294 | 0.265 | 0.274 | 0.277 | 0.192 | 0.121 | 0.379 | 0.269 | 0.066 | 0.244 |
| Mean | | 0.275 | 0.295 | 0.251 | 0.194 | 0.245 | 0.234 | 0.248 | 0.301 | 0.231 | 0.245 | 0.250 | 0.215 | 0.225 | 0.229 | 0.211 | 0.106 |  | 0.235 |  |  |
| Standard Deviation | | 0.094 | 0.075 | 0.088 | 0.060 | 0.074 | 0.113 | 0.113 | 0.095 | 0.115 | 0.071 | 0.071 | 0.099 | 0.130 | 0.077 | 0.101 | 0.129 |  |  |  |  |
| LOG-NICG-Z | DEG thresholds | 0.001 | 0.002 | 0.003 | 0.004 | 0.005 | 0.006 | 0.007 | 0.008 | 0.009 | 0.010 | 0.020 | 0.030 | 0.050 | 0.070 | 0.100 | 1.000 | max | Mean | Standard Deviation | Coefficient of Variation |
| NDEG thresholds | gene numbers | 11408 | 11790 | 12019 | 12189 | 12303 | 12427 | 12548 | 12621 | 12690 | 12757 | 13155 | 13409 | 13744 | 13955 | 14197 | 15672 |  |  |  |  |
| 0.980 | 6 | 0.168 | 0.208 | 0.111 | 0.204 | 0.196 | 0.166 | 0.057 | 0.162 | 0.208 | 0.149 | 0.117 | 0.089 | 0.116 | 0.225 | 0.184 | 0.101 | 0.225 | 0.154 | 0.050 | 0.325 |
| 0.950 | 22 | 0.253 | 0.177 | 0.066 | 0.140 | 0.157 | 0.130 | 0.093 | 0.227 | 0.089 | 0.192 | 0.149 | 0.092 | 0.124 | 0.093 | 0.091 | 0.297 | 0.297 | 0.148 | 0.066 | 0.448 |
| 0.920 | 39 | 0.248 | 0.077 | 0.145 | 0.160 | 0.225 | 0.205 | 0.264 | 0.129 | 0.084 | 0.166 | 0.110 | 0.263 | 0.222 | 0.117 | 0.049 | 0.118 | 0.264 | 0.161 | 0.069 | 0.427 |
| 0.900 | 58 | 0.106 | 0.084 | 0.207 | 0.068 | 0.163 | 0.167 | 0.128 | 0.252 | 0.157 | 0.256 | 0.262 | 0.145 | 0.168 | 0.072 | 0.087 | 0.209 | 0.262 | 0.158 | 0.065 | 0.414 |
| 0.850 | 97 | 0.187 | 0.111 | 0.188 | 0.121 | 0.195 | 0.145 | 0.103 | 0.233 | 0.123 | 0.145 | 0.137 | 0.124 | 0.090 | 0.097 | 0.135 | 0.176 | 0.233 | 0.144 | 0.041 | 0.282 |
| Mean | | 0.192 | 0.131 | 0.143 | 0.138 | 0.187 | 0.162 | 0.129 | 0.201 | 0.132 | 0.181 | 0.155 | 0.143 | 0.144 | 0.121 | 0.109 | 0.180 |  | 0.153 |  |  |
| Standard Deviation | | 0.061 | 0.058 | 0.057 | 0.050 | 0.028 | 0.028 | 0.079 | 0.053 | 0.052 | 0.046 | 0.062 | 0.071 | 0.052 | 0.060 | 0.052 | 0.079 |  |  |  |  |
|  |  |  |  |  |  |  |  |  |  |  |  |  |  |  |  |  |  |  |  |  |  |
| LR | |  |  |  |  |  |  |  |  |  |  |  |  |  |  |  |  |  |  |  |  |
| LOG_NPN_Z | DEG thresholds | 0.001 | 0.002 | 0.003 | 0.004 | 0.005 | 0.006 | 0.007 | 0.008 | 0.009 | 0.010 | 0.020 | 0.030 | 0.050 | 0.070 | 0.100 | 1.000 | max | Mean | Standard Deviation | Coefficient of Variation |
| NDEG thresholds | gene numbers | 11408 | 11790 | 12019 | 12189 | 12303 | 12427 | 12548 | 12621 | 12690 | 12757 | 13155 | 13409 | 13744 | 13955 | 14197 | 15672 |  |  |  |  |
| 0.980 | 6 | 0.099 | 0.178 | 0.120 | 0.174 | 0.190 | 0.171 | 0.070 | 0.077 | 0.128 | 0.177 | 0.130 | 0.142 | 0.115 | 0.142 | 0.075 | 0.106 | 0.190 | 0.131 | 0.039 | 0.300 |
| 0.950 | 22 | 0.135 | 0.122 | 0.090 | 0.149 | 0.164 | 0.130 | 0.126 | 0.145 | 0.123 | 0.044 | 0.193 | 0.054 | 0.091 | 0.098 | 0.076 | 0.039 | 0.193 | 0.111 | 0.044 | 0.393 |
| 0.920 | 39 | 0.110 | 0.129 | 0.078 | 0.090 | 0.172 | 0.152 | 0.081 | 0.145 | 0.083 | 0.051 | 0.146 | 0.048 | 0.104 | 0.132 | 0.050 | 0.097 | 0.172 | 0.104 | 0.039 | 0.373 |
| 0.90 | 58 | 0.205 | 0.171 | 0.072 | 0.124 | 0.175 | 0.138 | 0.101 | 0.164 | 0.110 | 0.137 | 0.049 | 0.073 | 0.085 | 0.068 | 0.045 | 0.114 | 0.205 | 0.114 | 0.048 | 0.420 |
| 0.850 | 97 | 0.155 | 0.145 | 0.075 | 0.176 | 0.208 | 0.154 | 0.094 | 0.090 | 0.090 | 0.139 | 0.061 | 0.145 | 0.114 | 0.175 | 0.080 | 0.059 | 0.208 | 0.122 | 0.046 | 0.373 |
| Mean | | 0.141 | 0.149 | 0.087 | 0.143 | 0.182 | 0.149 | 0.095 | 0.124 | 0.107 | 0.110 | 0.116 | 0.092 | 0.102 | 0.123 | 0.065 | 0.083 |  | 0.117 |  |  |
| Standard Deviation | | 0.042 | 0.025 | 0.020 | 0.036 | 0.018 | 0.016 | 0.021 | 0.038 | 0.020 | 0.059 | 0.060 | 0.047 | 0.013 | 0.041 | 0.017 | 0.033 |  |  |  |  |
| LOG-RQN | DEG thresholds | 0.001 | 0.002 | 0.003 | 0.004 | 0.005 | 0.006 | 0.007 | 0.008 | 0.009 | 0.010 | 0.020 | 0.030 | 0.050 | 0.070 | 0.100 | 1.000 | max | Mean | Standard Deviation | Coefficient of Variation |
| NDEG thresholds | gene numbers | 11408 | 11790 | 12019 | 12189 | 12303 | 12427 | 12548 | 12621 | 12690 | 12757 | 13155 | 13409 | 13744 | 13955 | 14197 | 15672 |  |  |  |  |
| 0.980 | 6 | 0.675 | 0.652 | 0.650 | 0.659 | 0.644 | 0.648 | 0.647 | 0.721 | 0.615 | 0.665 | 0.613 | 0.618 | 0.581 | 0.672 | 0.650 | 0.701 | 0.721 | 0.651 | 0.034 | 0.052 |
| 0.950 | 22 | 0.662 | 0.610 | 0.671 | 0.652 | 0.682 | 0.585 | 0.648 | 0.661 | 0.650 | 0.540 | 0.635 | 0.610 | 0.485 | 0.556 | 0.645 | 0.484 | 0.682 | 0.611 | 0.064 | 0.104 |
| 0.920 | 39 | 0.595 | 0.617 | 0.610 | 0.601 | 0.586 | 0.611 | 0.634 | 0.571 | 0.594 | 0.556 | 0.576 | 0.582 | 0.486 | 0.620 | 0.619 | 0.444 | 0.634 | 0.581 | 0.050 | 0.087 |
| 0.900 | 58 | 0.666 | 0.621 | 0.633 | 0.585 | 0.685 | 0.649 | 0.647 | 0.698 | 0.598 | 0.638 | 0.560 | 0.662 | 0.547 | 0.618 | 0.659 | 0.604 | 0.698 | 0.629 | 0.043 | 0.068 |
| 0.850 | 97 | 0.660 | 0.678 | 0.611 | 0.649 | 0.643 | 0.669 | 0.636 | 0.666 | 0.627 | 0.627 | 0.574 | 0.647 | 0.631 | 0.585 | 0.617 | 0.626 | 0.678 | 0.634 | 0.029 | 0.045 |
| Mean | | 0.652 | 0.636 | 0.635 | 0.629 | 0.648 | 0.632 | 0.642 | 0.663 | 0.617 | 0.605 | 0.592 | 0.624 | 0.546 | 0.610 | 0.638 | 0.572 |  | 0.621 |  |  |
| Standard Deviation | | 0.032 | 0.029 | 0.026 | 0.034 | 0.040 | 0.034 | 0.007 | 0.057 | 0.023 | 0.054 | 0.031 | 0.031 | 0.063 | 0.043 | 0.019 | 0.106 |  |  |  |  |
| LOG-RQN-Z | DEG thresholds | 0.001 | 0.002 | 0.003 | 0.004 | 0.005 | 0.006 | 0.007 | 0.008 | 0.009 | 0.010 | 0.020 | 0.030 | 0.050 | 0.070 | 0.100 | 1.000 | max | Mean | Standard Deviation | Coefficient of Variation |
| NDEG thresholds | gene numbers | 11408 | 11790 | 12019 | 12189 | 12303 | 12427 | 12548 | 12621 | 12690 | 12757 | 13155 | 13409 | 13744 | 13955 | 14197 | 15672 |  |  |  |  |
| 0.980 | 6 | 0.649 | 0.639 | 0.689 | 0.679 | 0.667 | 0.673 | 0.682 | 0.681 | 0.653 | 0.669 | 0.644 | 0.638 | 0.661 | 0.653 | 0.628 | 0.716 | 0.716 | 0.664 | 0.023 | 0.034 |
| 0.950 | 22 | 0.690 | 0.666 | 0.670 | 0.607 | 0.677 | 0.521 | 0.658 | 0.710 | 0.630 | 0.543 | 0.591 | 0.479 | 0.463 | 0.555 | 0.415 | 0.328 | 0.710 | 0.575 | 0.110 | 0.192 |
| 0.920 | 39 | 0.605 | 0.587 | 0.635 | 0.649 | 0.588 | 0.616 | 0.582 | 0.635 | 0.613 | 0.607 | 0.557 | 0.563 | 0.496 | 0.619 | 0.578 | 0.316 | 0.649 | 0.578 | 0.079 | 0.137 |
| 0.900 | 58 | 0.656 | 0.645 | 0.640 | 0.639 | 0.659 | 0.589 | 0.671 | 0.680 | 0.637 | 0.573 | 0.571 | 0.589 | 0.538 | 0.622 | 0.575 | 0.573 | 0.680 | 0.616 | 0.043 | 0.070 |
| 0.850 | 97 | 0.668 | 0.616 | 0.636 | 0.596 | 0.652 | 0.644 | 0.663 | 0.647 | 0.610 | 0.614 | 0.567 | 0.691 | 0.554 | 0.574 | 0.572 | 0.616 | 0.691 | 0.620 | 0.040 | 0.065 |
| Mean | | 0.654 | 0.631 | 0.654 | 0.634 | 0.648 | 0.609 | 0.651 | 0.671 | 0.629 | 0.601 | 0.586 | 0.592 | 0.542 | 0.604 | 0.553 | 0.510 |  | 0.611 |  |  |
| Standard Deviation | | 0.031 | 0.030 | 0.024 | 0.033 | 0.035 | 0.058 | 0.040 | 0.030 | 0.018 | 0.047 | 0.035 | 0.080 | 0.075 | 0.039 | 0.081 | 0.179 |  |  |  |  |
| LOG-NICG-Z | DEG thresholds | 0.001 | 0.002 | 0.003 | 0.004 | 0.005 | 0.006 | 0.007 | 0.008 | 0.009 | 0.010 | 0.020 | 0.030 | 0.050 | 0.070 | 0.100 | 1.000 | max | Mean | Standard Deviation | Coefficient of Variation |
| NDEG thresholds | gene numbers | 11408 | 11790 | 12019 | 12189 | 12303 | 12427 | 12548 | 12621 | 12690 | 12757 | 13155 | 13409 | 13744 | 13955 | 14197 | 15672 |  |  |  |  |
| 0.980 | 6 | 0.150 | 0.081 | 0.143 | 0.181 | 0.155 | 0.187 | 0.159 | 0.099 | 0.076 | 0.114 | 0.105 | 0.136 | 0.120 | 0.127 | 0.066 | 0.065 | 0.187 | 0.123 | 0.039 | 0.317 |
| 0.950 | 22 | 0.069 | 0.111 | 0.099 | 0.216 | 0.137 | 0.117 | 0.141 | 0.079 | 0.086 | 0.041 | 0.162 | 0.054 | 0.188 | 0.158 | 0.051 | 0.142 | 0.216 | 0.116 | 0.051 | 0.443 |
| 0.920 | 39 | 0.083 | 0.099 | 0.084 | 0.091 | 0.173 | 0.172 | 0.155 | 0.084 | 0.117 | 0.078 | 0.139 | 0.071 | 0.104 | 0.070 | 0.067 | 0.120 | 0.173 | 0.107 | 0.036 | 0.335 |
| 0.900 | 58 | 0.193 | 0.115 | 0.106 | 0.190 | 0.175 | 0.137 | 0.147 | 0.166 | 0.075 | 0.070 | 0.073 | 0.164 | 0.033 | 0.100 | 0.081 | 0.163 | 0.193 | 0.124 | 0.049 | 0.396 |
| 0.850 | 97 | 0.105 | 0.074 | 0.069 | 0.163 | 0.156 | 0.154 | 0.067 | 0.057 | 0.092 | 0.055 | 0.058 | 0.158 | 0.102 | 0.034 | 0.146 | 0.115 | 0.163 | 0.100 | 0.044 | 0.436 |
| Mean | | 0.120 | 0.096 | 0.100 | 0.168 | 0.159 | 0.154 | 0.134 | 0.097 | 0.089 | 0.072 | 0.107 | 0.117 | 0.109 | 0.098 | 0.082 | 0.121 |  | 0.114 |  |  |
| Standard Deviation | | 0.051 | 0.018 | 0.028 | 0.047 | 0.015 | 0.028 | 0.038 | 0.042 | 0.017 | 0.028 | 0.043 | 0.051 | 0.055 | 0.048 | 0.037 | 0.036 |  |  |  |  |
|  |  |  |  |  |  |  |  |  |  |  |  |  |  |  |  |  |  |  |  |  |  |
| MLP | |  |  |  |  |  |  |  |  |  |  |  |  |  |  |  |  |  |  |  |  |
| LOG_NPN_Z | DEG thresholds | 0.001 | 0.002 | 0.003 | 0.004 | 0.005 | 0.006 | 0.007 | 0.008 | 0.009 | 0.010 | 0.020 | 0.030 | 0.050 | 0.070 | 0.100 | 1.000 | max | Mean | Standard Deviation | Coefficient of Variation |
| NDEG thresholds | gene numbers | 11408 | 11790 | 12019 | 12189 | 12303 | 12427 | 12548 | 12621 | 12690 | 12757 | 13155 | 13409 | 13744 | 13955 | 14197 | 15672 |  |  |  |  |
| 0.980 | 6 | 0.297 | 0.187 | 0.236 | 0.153 | 0.267 | 0.183 | 0.209 | 0.193 | 0.232 | 0.226 | 0.204 | 0.205 | 0.178 | 0.248 | 0.191 | 0.172 | 0.297 | 0.211 | 0.038 | 0.178 |
| 0.950 | 22 | 0.216 | 0.265 | 0.232 | 0.228 | 0.199 | 0.205 | 0.180 | 0.185 | 0.248 | 0.112 | 0.090 | 0.160 | 0.175 | 0.238 | 0.254 | 0.152 | 0.265 | 0.196 | 0.050 | 0.255 |
| 0.920 | 39 | 0.257 | 0.169 | 0.180 | 0.213 | 0.222 | 0.232 | 0.140 | 0.202 | 0.066 | 0.167 | 0.212 | 0.126 | 0.228 | 0.126 | 0.212 | 0.175 | 0.257 | 0.183 | 0.050 | 0.271 |
| 0.900 | 58 | 0.223 | 0.258 | 0.191 | 0.188 | 0.211 | 0.243 | 0.174 | 0.152 | 0.225 | 0.241 | 0.205 | 0.113 | 0.135 | 0.141 | 0.099 | 0.217 | 0.258 | 0.188 | 0.048 | 0.257 |
| 0.850 | 97 | 0.282 | 0.288 | 0.229 | 0.217 | 0.284 | 0.181 | 0.252 | 0.180 | 0.228 | 0.200 | 0.142 | 0.054 | 0.093 | 0.251 | 0.229 | 0.143 | 0.288 | 0.203 | 0.068 | 0.335 |
| Mean | | 0.255 | 0.234 | 0.214 | 0.200 | 0.237 | 0.209 | 0.191 | 0.182 | 0.200 | 0.189 | 0.170 | 0.132 | 0.162 | 0.201 | 0.197 | 0.172 |  | 0.196 |  |  |
| Standard Deviation | | 0.036 | 0.052 | 0.026 | 0.030 | 0.037 | 0.028 | 0.042 | 0.019 | 0.075 | 0.051 | 0.053 | 0.056 | 0.051 | 0.062 | 0.060 | 0.028 |  |  |  |  |
| LOG-RQN | DEG thresholds | 0.001 | 0.002 | 0.003 | 0.004 | 0.005 | 0.006 | 0.007 | 0.008 | 0.009 | 0.010 | 0.020 | 0.030 | 0.050 | 0.070 | 0.100 | 1.000 | max | Mean | Standard Deviation | Coefficient of Variation |
| NDEG thresholds | gene numbers | 11408 | 11790 | 12019 | 12189 | 12303 | 12427 | 12548 | 12621 | 12690 | 12757 | 13155 | 13409 | 13744 | 13955 | 14197 | 15672 |  |  |  |  |
| 0.980 | 6 | 0.721 | 0.677 | 0.706 | 0.655 | 0.692 | 0.665 | 0.684 | 0.713 | 0.656 | 0.699 | 0.654 | 0.674 | 0.630 | 0.692 | 0.687 | 0.712 | 0.721 | 0.682 | 0.026 | 0.037 |
| 0.950 | 22 | 0.707 | 0.676 | 0.687 | 0.691 | 0.696 | 0.667 | 0.690 | 0.682 | 0.677 | 0.653 | 0.606 | 0.641 | 0.661 | 0.664 | 0.677 | 0.668 | 0.707 | 0.671 | 0.024 | 0.036 |
| 0.920 | 39 | 0.674 | 0.626 | 0.701 | 0.659 | 0.726 | 0.657 | 0.638 | 0.660 | 0.667 | 0.635 | 0.683 | 0.607 | 0.652 | 0.669 | 0.641 | 0.689 | 0.726 | 0.662 | 0.030 | 0.045 |
| 0.90 | 58 | 0.685 | 0.659 | 0.692 | 0.690 | 0.734 | 0.683 | 0.665 | 0.700 | 0.632 | 0.627 | 0.700 | 0.709 | 0.644 | 0.638 | 0.697 | 0.653 | 0.734 | 0.675 | 0.031 | 0.046 |
| 0.850 | 97 | 0.720 | 0.673 | 0.669 | 0.688 | 0.673 | 0.678 | 0.678 | 0.689 | 0.657 | 0.630 | 0.684 | 0.683 | 0.689 | 0.681 | 0.674 | 0.661 | 0.720 | 0.677 | 0.019 | 0.028 |
| Mean |  | 0.701 | 0.662 | 0.691 | 0.677 | 0.704 | 0.670 | 0.671 | 0.689 | 0.658 | 0.649 | 0.665 | 0.663 | 0.655 | 0.669 | 0.675 | 0.677 |  | 0.674 |  |  |
| Standard Deviation | | 0.021 | 0.021 | 0.014 | 0.018 | 0.025 | 0.010 | 0.021 | 0.020 | 0.017 | 0.030 | 0.037 | 0.039 | 0.022 | 0.021 | 0.021 | 0.024 |  |  |  |  |
| LOG-RQN-Z | DEG thresholds | 0.001 | 0.002 | 0.003 | 0.004 | 0.005 | 0.006 | 0.007 | 0.008 | 0.009 | 0.010 | 0.020 | 0.030 | 0.050 | 0.070 | 0.100 | 1.000 | max | Mean | Standard Deviation | Coefficient of Variation |
| NDEG thresholds | gene numbers | 11408 | 11790 | 12019 | 12189 | 12303 | 12427 | 12548 | 12621 | 12690 | 12757 | 13155 | 13409 | 13744 | 13955 | 14197 | 15672 |  |  |  |  |
| 0.980 | 6 | 0.695 | 0.667 | 0.701 | 0.703 | 0.679 | 0.620 | 0.623 | 0.701 | 0.678 | 0.677 | 0.669 | 0.652 | 0.673 | 0.643 | 0.683 | 0.670 | 0.703 | 0.671 | 0.025 | 0.038 |
| 0.950 | 22 | 0.716 | 0.689 | 0.683 | 0.672 | 0.680 | 0.661 | 0.692 | 0.690 | 0.637 | 0.649 | 0.624 | 0.626 | 0.594 | 0.650 | 0.660 | 0.660 | 0.716 | 0.661 | 0.031 | 0.047 |
| 0.920 | 39 | 0.654 | 0.703 | 0.691 | 0.670 | 0.697 | 0.676 | 0.661 | 0.660 | 0.628 | 0.631 | 0.689 | 0.650 | 0.634 | 0.612 | 0.673 | 0.600 | 0.703 | 0.658 | 0.031 | 0.047 |
| 0.900 | 58 | 0.679 | 0.704 | 0.677 | 0.657 | 0.683 | 0.693 | 0.671 | 0.679 | 0.655 | 0.649 | 0.686 | 0.688 | 0.676 | 0.659 | 0.685 | 0.651 | 0.704 | 0.674 | 0.016 | 0.024 |
| 0.850 | 97 | 0.707 | 0.704 | 0.673 | 0.688 | 0.679 | 0.662 | 0.703 | 0.681 | 0.624 | 0.639 | 0.674 | 0.725 | 0.662 | 0.658 | 0.665 | 0.676 | 0.725 | 0.676 | 0.026 | 0.038 |
| Mean |  | 0.690 | 0.693 | 0.685 | 0.678 | 0.684 | 0.662 | 0.670 | 0.682 | 0.644 | 0.649 | 0.668 | 0.668 | 0.648 | 0.644 | 0.673 | 0.651 |  | 0.668 |  |  |
| Standard Deviation | | 0.025 | 0.016 | 0.011 | 0.018 | 0.008 | 0.027 | 0.031 | 0.015 | 0.022 | 0.017 | 0.026 | 0.039 | 0.034 | 0.019 | 0.011 | 0.031 |  |  |  |  |
| LOG-NICG-Z | DEG thresholds | 0.001 | 0.002 | 0.003 | 0.004 | 0.005 | 0.006 | 0.007 | 0.008 | 0.009 | 0.010 | 0.020 | 0.030 | 0.050 | 0.070 | 0.100 | 1.000 | max | Mean | Standard Deviation | Coefficient of Variation |
| NDEG thresholds | gene numbers | 11408 | 11790 | 12019 | 12189 | 12303 | 12427 | 12548 | 12621 | 12690 | 12757 | 13155 | 13409 | 13744 | 13955 | 14197 | 15672 |  |  |  |  |
| 0.980 | 6 | 0.223 | 0.200 | 0.118 | 0.114 | 0.212 | 0.097 | 0.111 | 0.110 | 0.203 | 0.061 | 0.246 | 0.166 | 0.176 | 0.204 | 0.116 | 0.155 | 0.246 | 0.157 | 0.054 | 0.345 |
| 0.950 | 22 | 0.208 | 0.161 | 0.114 | 0.160 | 0.129 | 0.168 | 0.102 | 0.100 | 0.230 | 0.153 | 0.140 | 0.140 | 0.104 | 0.188 | 0.136 | 0.093 | 0.230 | 0.145 | 0.040 | 0.274 |
| 0.920 | 39 | 0.123 | 0.170 | 0.093 | 0.223 | 0.158 | 0.201 | 0.115 | 0.153 | 0.166 | 0.142 | 0.232 | 0.206 | 0.165 | 0.220 | 0.206 | 0.008 | 0.232 | 0.161 | 0.058 | 0.359 |
| 0.900 | 58 | 0.236 | 0.125 | 0.167 | 0.217 | 0.170 | 0.179 | 0.066 | 0.126 | 0.172 | 0.121 | 0.166 | 0.083 | 0.054 | 0.196 | 0.100 | 0.154 | 0.236 | 0.146 | 0.052 | 0.360 |
| 0.850 | 97 | 0.138 | 0.159 | 0.210 | 0.163 | 0.096 | 0.200 | 0.179 | 0.111 | 0.191 | 0.094 | 0.172 | 0.176 | 0.189 | 0.068 | 0.170 | 0.126 | 0.210 | 0.153 | 0.042 | 0.277 |
| Mean |  | 0.186 | 0.163 | 0.140 | 0.175 | 0.153 | 0.169 | 0.115 | 0.120 | 0.192 | 0.114 | 0.191 | 0.154 | 0.138 | 0.175 | 0.146 | 0.107 |  | 0.152 |  |  |
| Standard Deviation | | 0.052 | 0.027 | 0.047 | 0.045 | 0.044 | 0.042 | 0.041 | 0.021 | 0.026 | 0.037 | 0.046 | 0.046 | 0.057 | 0.061 | 0.043 | 0.061 |  |  |  |  |
|  |  |  |  |  |  |  |  |  |  |  |  |  |  |  |  |  |  |  |  |  |  |
| XGB | |  |  |  |  |  |  |  |  |  |  |  |  |  |  |  |  |  |  |  |  |
| LOG_NPN_Z | DEG thresholds | 0.001 | 0.002 | 0.003 | 0.004 | 0.005 | 0.006 | 0.007 | 0.008 | 0.009 | 0.010 | 0.020 | 0.030 | 0.050 | 0.070 | 0.100 | 1.000 | max | Mean | Standard Deviation | Coefficient of Variation |
| NDEG thresholds | gene numbers | 11408 | 11790 | 12019 | 12189 | 12303 | 12427 | 12548 | 12621 | 12690 | 12757 | 13155 | 13409 | 13744 | 13955 | 14197 | 15672 |  |  |  |  |
| 0.980 | 6 | 0.492 | 0.437 | 0.279 | 0.251 | 0.354 | 0.302 | 0.307 | 0.366 | 0.300 | 0.380 | 0.323 | 0.403 | 0.283 | 0.296 | 0.257 | 0.338 | 0.492 | 0.336 | 0.067 | 0.199 |
| 0.950 | 22 | 0.226 | 0.368 | 0.350 | 0.200 | 0.346 | 0.345 | 0.267 | 0.290 | 0.296 | 0.161 | 0.264 | 0.223 | 0.321 | 0.256 | 0.205 | 0.281 | 0.368 | 0.275 | 0.061 | 0.223 |
| 0.920 | 39 | 0.332 | 0.344 | 0.379 | 0.199 | 0.374 | 0.199 | 0.139 | 0.298 | 0.284 | 0.247 | 0.340 | 0.271 | 0.265 | 0.242 | 0.119 | 0.201 | 0.379 | 0.265 | 0.079 | 0.298 |
| 0.900 | 58 | 0.241 | 0.345 | 0.189 | 0.179 | 0.272 | 0.242 | 0.237 | 0.156 | 0.191 | 0.128 | 0.298 | 0.266 | 0.322 | 0.145 | 0.155 | 0.424 | 0.424 | 0.237 | 0.082 | 0.347 |
| 0.850 | 97 | 0.243 | 0.306 | 0.281 | 0.316 | 0.342 | 0.373 | 0.271 | 0.151 | 0.272 | 0.262 | 0.258 | 0.456 | 0.250 | 0.274 | 0.309 | 0.391 | 0.456 | 0.297 | 0.070 | 0.236 |
| Mean | | 0.307 | 0.360 | 0.296 | 0.229 | 0.338 | 0.292 | 0.244 | 0.252 | 0.269 | 0.236 | 0.296 | 0.324 | 0.288 | 0.242 | 0.209 | 0.327 |  | 0.282 |  |  |
| Standard Deviation | | 0.112 | 0.048 | 0.074 | 0.056 | 0.039 | 0.072 | 0.064 | 0.095 | 0.044 | 0.099 | 0.036 | 0.100 | 0.033 | 0.058 | 0.076 | 0.089 |  |  |  |  |
| LOG-RQN | DEG thresholds | 0.001 | 0.002 | 0.003 | 0.004 | 0.005 | 0.006 | 0.007 | 0.008 | 0.009 | 0.010 | 0.020 | 0.030 | 0.050 | 0.070 | 0.100 | 1.000 | max | Mean | Standard Deviation | Coefficient of Variation |
| NDEG thresholds | gene numbers | 11408 | 11790 | 12019 | 12189 | 12303 | 12427 | 12548 | 12621 | 12690 | 12757 | 13155 | 13409 | 13744 | 13955 | 14197 | 15672 |  |  |  |  |
| 0.980 | 6 | 0.494 | 0.479 | 0.480 | 0.486 | 0.462 | 0.494 | 0.472 | 0.462 | 0.479 | 0.473 | 0.449 | 0.437 | 0.515 | 0.488 | 0.453 | 0.363 | 0.515 | 0.468 | 0.034 | 0.072 |
| 0.950 | 22 | 0.383 | 0.386 | 0.370 | 0.353 | 0.359 | 0.267 | 0.402 | 0.487 | 0.296 | 0.387 | 0.342 | 0.208 | 0.268 | 0.298 | 0.326 | 0.199 | 0.487 | 0.333 | 0.075 | 0.225 |
| 0.920 | 39 | 0.315 | 0.291 | 0.341 | 0.283 | 0.322 | 0.316 | 0.327 | 0.253 | 0.319 | 0.366 | 0.303 | 0.235 | 0.293 | 0.281 | 0.304 | 0.151 | 0.366 | 0.294 | 0.050 | 0.169 |
| 0.90 | 58 | 0.416 | 0.424 | 0.470 | 0.316 | 0.367 | 0.398 | 0.461 | 0.436 | 0.402 | 0.450 | 0.343 | 0.337 | 0.411 | 0.398 | 0.325 | 0.227 | 0.470 | 0.386 | 0.064 | 0.165 |
| 0.850 | 97 | 0.512 | 0.470 | 0.439 | 0.405 | 0.346 | 0.391 | 0.469 | 0.440 | 0.374 | 0.364 | 0.413 | 0.355 | 0.405 | 0.404 | 0.402 | 0.259 | 0.512 | 0.403 | 0.059 | 0.147 |
| Mean |  | 0.424 | 0.410 | 0.420 | 0.369 | 0.371 | 0.373 | 0.426 | 0.416 | 0.374 | 0.408 | 0.370 | 0.314 | 0.378 | 0.374 | 0.362 | 0.240 |  | 0.377 |  |  |
| Standard Deviation | | 0.081 | 0.076 | 0.061 | 0.080 | 0.054 | 0.087 | 0.062 | 0.093 | 0.072 | 0.050 | 0.060 | 0.093 | 0.100 | 0.085 | 0.063 | 0.080 |  |  |  |  |
| LOG-RQN-Z | DEG thresholds | 0.001 | 0.002 | 0.003 | 0.004 | 0.005 | 0.006 | 0.007 | 0.008 | 0.009 | 0.010 | 0.020 | 0.030 | 0.050 | 0.070 | 0.100 | 1.000 | max | Mean | Standard Deviation | Coefficient of Variation |
| NDEG thresholds | gene numbers | 11408 | 11790 | 12019 | 12189 | 12303 | 12427 | 12548 | 12621 | 12690 | 12757 | 13155 | 13409 | 13744 | 13955 | 14197 | 15672 |  |  |  |  |
| 0.980 | 6 | 0.524 | 0.447 | 0.465 | 0.484 | 0.477 | 0.482 | 0.481 | 0.525 | 0.403 | 0.439 | 0.444 | 0.466 | 0.456 | 0.522 | 0.466 | 0.463 | 0.525 | 0.471 | 0.033 | 0.069 |
| 0.950 | 22 | 0.380 | 0.368 | 0.386 | 0.342 | 0.395 | 0.286 | 0.381 | 0.456 | 0.343 | 0.372 | 0.335 | 0.270 | 0.317 | 0.275 | 0.343 | 0.209 | 0.456 | 0.341 | 0.060 | 0.175 |
| 0.920 | 39 | 0.266 | 0.244 | 0.382 | 0.363 | 0.340 | 0.309 | 0.309 | 0.283 | 0.286 | 0.343 | 0.268 | 0.263 | 0.272 | 0.272 | 0.298 | 0.162 | 0.382 | 0.291 | 0.052 | 0.179 |
| 0.900 | 58 | 0.437 | 0.419 | 0.418 | 0.280 | 0.411 | 0.415 | 0.396 | 0.483 | 0.327 | 0.433 | 0.375 | 0.350 | 0.427 | 0.393 | 0.314 | 0.295 | 0.483 | 0.386 | 0.057 | 0.148 |
| 0.850 | 97 | 0.447 | 0.432 | 0.425 | 0.354 | 0.391 | 0.393 | 0.408 | 0.430 | 0.365 | 0.330 | 0.443 | 0.404 | 0.441 | 0.362 | 0.432 | 0.294 | 0.447 | 0.397 | 0.045 | 0.113 |
| Mean |  | 0.411 | 0.382 | 0.415 | 0.365 | 0.403 | 0.377 | 0.395 | 0.435 | 0.345 | 0.383 | 0.373 | 0.351 | 0.383 | 0.365 | 0.371 | 0.285 |  | 0.377 |  |  |
| Standard Deviation | | 0.096 | 0.083 | 0.034 | 0.074 | 0.049 | 0.080 | 0.061 | 0.092 | 0.044 | 0.050 | 0.075 | 0.087 | 0.082 | 0.103 | 0.074 | 0.115 |  |  |  |  |
| LOG-NICG-Z | DEG thresholds | 0.001 | 0.002 | 0.003 | 0.004 | 0.005 | 0.006 | 0.007 | 0.008 | 0.009 | 0.010 | 0.020 | 0.030 | 0.050 | 0.070 | 0.100 | 1.000 | max | Mean | Standard Deviation | Coefficient of Variation |
| NDEG thresholds | gene numbers | 11408 | 11790 | 12019 | 12189 | 12303 | 12427 | 12548 | 12621 | 12690 | 12757 | 13155 | 13409 | 13744 | 13955 | 14197 | 15672 |  |  |  |  |
| 0.980 | 6 | 0.125 | 0.245 | 0.139 | 0.186 | 0.254 | 0.120 | 0.154 | 0.355 | 0.174 | 0.200 | 0.144 | 0.152 | 0.265 | 0.134 | 0.181 | 0.065 | 0.355 | 0.181 | 0.070 | 0.389 |
| 0.950 | 22 | 0.120 | 0.362 | 0.193 | 0.143 | 0.229 | 0.210 | 0.168 | 0.273 | 0.317 | 0.209 | 0.232 | 0.106 | 0.316 | 0.179 | 0.182 | 0.185 | 0.362 | 0.214 | 0.072 | 0.338 |
| 0.920 | 39 | 0.139 | 0.212 | 0.102 | 0.114 | 0.217 | 0.234 | 0.188 | 0.209 | 0.216 | 0.195 | 0.251 | 0.151 | 0.259 | 0.226 | 0.163 | 0.073 | 0.259 | 0.184 | 0.055 | 0.299 |
| 0.90 | 58 | 0.110 | 0.244 | 0.233 | 0.179 | 0.210 | 0.206 | 0.044 | 0.122 | 0.185 | 0.185 | 0.140 | 0.258 | 0.300 | 0.067 | 0.209 | 0.167 | 0.300 | 0.179 | 0.069 | 0.386 |
| 0.850 | 97 | 0.117 | 0.207 | 0.224 | 0.219 | 0.244 | 0.259 | 0.236 | 0.127 | 0.228 | 0.183 | 0.224 | 0.206 | 0.206 | 0.170 | 0.159 | 0.172 | 0.259 | 0.199 | 0.041 | 0.205 |
| Mean |  | 0.122 | 0.254 | 0.178 | 0.168 | 0.231 | 0.206 | 0.158 | 0.217 | 0.224 | 0.194 | 0.198 | 0.175 | 0.269 | 0.155 | 0.179 | 0.133 |  | 0.191 |  |  |
| Standard Deviation | | 0.011 | 0.063 | 0.056 | 0.040 | 0.018 | 0.052 | 0.071 | 0.099 | 0.057 | 0.011 | 0.052 | 0.059 | 0.043 | 0.059 | 0.020 | 0.058 |  |  |  |  |
