# Supplementary material for: Normalization and Selecting Non-Differentially Expressed Genes Improve Machine Learning Modelling of Cross-Platform Transcriptomic Data
Source: Trans Artif Intell. Author manuscript; Available in PMC 2025 Jul 8. (PMC12235674; doi:10.53941/tai.2025.100005)
Supplement: Supplementary [file NIHMS2087281-supplement-Supplementary.zip › Supplementary table 7.docx]

| Supplementary table 7. Average performance results (mean ± standard deviation) of the best-performing models (with the highest Balanced Accuracy) on data constructed using DEG and NDEG genes selected via one-way ANOVA. (**Model-S**) | | | | | | | | | | | | |
| --- | --- | --- | --- | --- | --- | --- | --- | --- | --- | --- | --- | --- |
| Normalization _Method | DEG_ number | NDEG_ number | Model | E*_value_* | Kappa | Balanced _Accuracy | Accuracy | Precision | Recall | F1 | AUC | Confusion Matrix |
| LOG-NPN-Z | 10523 | 253 | SVM | 210.909 | 0.448  ±0.018 | 0.655  ±0.042 | 0.862  ±0.076 | 0.758  ±0.048 | 0.608  ±0.026 | 0.588  ±0.008 | 0.792  ±0.072 | [[20.80 Â± 1.47, 0.40 Â± 0.49, 0.00 Â± 0.00, 1.40 Â± 0.42, 0.00 Â± 0.00],  [0.60 Â± 0.49, 7.40 Â± 0.49, 3.40 Â± 0.49, 1.60 Â± 0.49, 0.00 Â± 0.00],  [0.60 Â± 0.49, 0.60 Â± 0.49, 23.60 Â± 1.96, 4.40 Â± 0.40, 0.00 Â± 0.00],  [0.00 Â± 0.00, 0.40 Â± 0.49, 0.60 Â± 0.49, 50.20 Â± 0.98, 0.00 Â± 0.00],  [1.00 Â± 0.20, 0.00 Â± 0.00, 0.60 Â± 0.49, 0.40 Â± 0.45, 0.00 Â± 0.00]] |
| LOG-RQN | 11079 | 133 | SVM | 378.907 | 0.760  ±0.017 | 0.773  ±0.093 | 0.883  ±0.014 | 0.878  ±0.022 | 0.831  ±0.015 | 0.831  ±0.007 | 0.961  ±0.007 | [[19.40 Â± 1.96, 0.80 Â± 0.98, 0.00 Â± 0.00, 0.40 Â± 0.49, 1.00 Â± 0.20],  [0.60 Â± 0.49, 11.00 Â± 0.00, 2.80 Â± 0.98, 0.00 Â± 0.00, 0.00 Â± 0.00],  [0.60 Â± 0.49, 1.80 Â± 1.47, 22.80 Â± 3.43, 3.20 Â± 1.47, 0.00 Â± 0.00],  [0.00 Â± 0.00, 0.60 Â± 0.49, 1.60 Â± 0.49, 51.00 Â± 2.45, 0.00 Â± 0.00],  [0.40 Â± 0.49, 0.00 Â± 0.00, 0.00 Â± 0.00, 0.00 Â± 0.00, 0.00 Â± 0.00]] |
| LOG-RQN-Z | 10936 | 94 | SVM | 313.309 | 0.755  ±0.037 | 0.726  ±0.089 | 0.893  ±0.028 | 0.847  ±0.017 | 0.825  ±0.026 | 0.829  ±0.027 | 0.949  ±0.008 | [[21.00 Â± 0.00, 0.00 Â± 0.00, 0.00 Â± 0.00, 0.00 Â± 0.00, 0.40 Â± 0.49],  [0.00 Â± 0.00, 11.40 Â± 0.49, 1.00 Â± 0.00, 1.20 Â± 0.98, 0.00 Â± 0.00],  [1.00 Â± 0.00, 0.60 Â± 0.49, 22.40 Â± 0.49, 3.00 Â± 0.00, 0.00 Â± 0.00],  [0.00 Â± 0.00, 0.00 Â± 0.00, 3.40 Â± 1.96, 50.60 Â± 1.96, 0.00 Â± 0.00],  [1.00 Â± 0.00, 0.00 Â± 0.00, 0.60 Â± 0.49, 0.40 Â± 0.49, 0.00 Â± 0.00]] |
| LOG-NICG-Z | 11516 | 94 | SVM | 89.519 | 0.441  ±0.186 | 0.626  ±0.210 | 0.791  ±0.011 | 0.627  ±0.105 | 0.632  ±0.094 | 0.565  ±0.141 | 0.815  ±0.058 | [[20.00 Â± 0.00, 0.00 Â± 0.00, 0.00 Â± 0.00, 0.40 Â± 0.49, 0.00 Â± 0.00],  [2.00 Â± 0.00, 6.00 Â± 0.00, 3.60 Â± 0.49, 2.40 Â± 0.49, 0.00 Â± 0.00],  [1.20 Â± 0.98, 0.60 Â± 0.49, 24.00 Â± 2.45, 4.80 Â± 1.47, 0.00 Â± 0.00],  [3.00 Â± 2.45, 0.00 Â± 0.00, 0.40 Â± 0.49, 48.00 Â± 2.45, 0.00 Â± 0.00],  [0.60 Â± 0.49, 0.00 Â± 0.00, 0.60 Â± 0.49, 0.40 Â± 0.49, 0.00 Â± 0.00]] |
| LOG-NPN-Z | 11516 | 94 | RF | 85.940 | 0.406  ±0.104 | 0.407  0.053 | 0.646  ±0.051 | 0.674  ±0.162 | 0.644  ±0.050 | 0.560  ±0.079 | 0.843  ±0.007 | [[8.40 Â± 0.49, 0.00 Â± 0.00, 1.60 Â± 0.49, 11.40 Â± 0.49, 0.00 Â± 0.00],  [0.40 Â± 0.49, 2.00 Â± 0.00, 4.40 Â± 0.49, 6.80 Â± 1.47, 0.00 Â± 0.00],  [1.60 Â± 0.49, 6.00 Â± 4.90, 15.20 Â± 3.92, 6.60 Â± 1.96, 0.00 Â± 0.00],  [0.40 Â± 0.49, 0.60 Â± 0.49, 0.00 Â± 0.00, 50.60 Â± 0.49, 0.00 Â± 0.00],  [1.00 Â± 0.00, 0.60 Â± 0.49, 0.00 Â± 0.00, 0.40 Â± 0.49, 0.00 Â± 0.00]] |
| LOG-RQN | 11183 | 11 | RF | 298.575 | 0.738  ±0.031 | 0.585  ±0.032 | 0.815  ±0.021 | 0.804  ±0.063 | 0.824  ±0.020 | 0.788  ±0.029 | 0.931  ±0.015 | [[18.20 Â± 1.47, 0.00 Â± 0.00, 0.00 Â± 0.00, 1.00 Â± 0.00, 0.00 Â± 0.00],  [0.40 Â± 0.49, 4.00 Â± 2.45, 5.20 Â± 3.92, 4.80 Â± 1.47, 0.00 Â± 0.00],  [1.00 Â± 0.00, 1.20 Â± 0.98, 22.00 Â± 2.45, 6.60 Â± 0.49, 0.00 Â± 0.00],  [0.00 Â± 0.00, 0.00 Â± 0.00, 0.00 Â± 0.00, 52.00 Â± 2.45, 0.00 Â± 0.00],  [0.40 Â± 0.49, 0.60 Â± 0.49, 0.00 Â± 0.00, 0.60 Â± 0.49, 0.00 Â± 0.00]] |
| LOG-RQN-Z | 15672 | 11 | RF | 271.629 | 0.734  ±0.044 | 0.588  ±0.042 | 0.807  ±0.032 | 0.795  ±0.057 | 0.820  ±0.030 | 0.792  ±0.043 | 0.920  ±0.025 | [[18.00 Â± 0.00, 0.40 Â± 0.49, 0.00 Â± 0.00, 0.40 Â± 0.49, 2.80 Â± 1.47],  [0.00 Â± 0.00, 9.40 Â± 0.49, 0.00 Â± 0.00, 5.00 Â± 0.00, 0.00 Â± 0.00],  [0.00 Â± 0.00, 0.00 Â± 0.00, 2.00 Â± 0.00, 24.60 Â± 1.96, 0.60 Â± 0.49],  [0.00 Â± 0.00, 0.00 Â± 0.00, 0.00 Â± 0.00, 54.00 Â± 2.45, 0.00 Â± 0.00],  [0.00 Â± 0.00, 0.00 Â± 0.00, 0.00 Â± 0.00, 0.80 Â± 0.98, 0.00 Â± 0.00]] |
| LOG-NICG-Z | 10756 | 133 | RF | 40.272 | 0.306  ±0.229 | 0.412  ±0.179 | 0.592  ±0.102 | 0.574  ±0.169 | 0.542  ±0.089 | 0.484  ±0.166 | 0.822  ±0.004 | [[12.40 Â± 1.96, 3.00 Â± 0.00, 0.00 Â± 0.00, 4.00 Â± 2.45, 2.20 Â± 0.98],  [0.00 Â± 0.00, 6.40 Â± 0.49, 0.00 Â± 0.00, 6.40 Â± 0.49, 0.00 Â± 0.00],  [0.00 Â± 0.00, 2.20 Â± 0.98, 0.00 Â± 0.00, 28.40 Â± 0.49, 0.00 Â± 0.00],  [0.00 Â± 0.00, 0.60 Â± 0.49, 0.00 Â± 0.00, 51.00 Â± 0.00, 0.00 Â± 0.00],  [0.60 Â± 0.49, 0.00 Â± 0.00, 0.00 Â± 0.00, 0.80 Â± 0.98, 0.00 Â± 0.00]] |
| LOG-NPN-Z | 11447 | 94 | LR | 202.280 | 0.539  ±0.038 | 0.671  ±0.098 | 0.732  ±0.042 | 0.672  ±0.121 | 0.703  ±0.033 | 0.622  ±0.019 | 0.908  ±0.006 | [[20.40 Â± 1.96, 0.40 Â± 0.49, 0.40 Â± 0.49, 0.00 Â± 0.00, 0.60 Â± 0.49],  [1.20 Â± 0.98, 10.60 Â± 0.49, 1.60 Â± 1.96, 0.00 Â± 0.00, 0.00 Â± 0.00],  [1.80 Â± 1.47, 2.40 Â± 1.96, 22.20 Â± 3.92, 3.20 Â± 0.98, 0.00 Â± 0.00],  [0.00 Â± 0.00, 0.00 Â± 0.00, 6.60 Â± 4.41, 45.00 Â± 2.45, 0.00 Â± 0.00],  [1.20 Â± 0.98, 0.00 Â± 0.00, 0.00 Â± 0.00, 0.40 Â± 0.49, 0.00 Â± 0.00]] |
| LOG-RQN | 11079 | 49 | LR | 400.110 | 0.819  ±0.030 | 0.749  ±0.049 | 0.876  ±0.021 | 0.876  ±0.025 | 0.875  ±0.021 | 0.867  ±0.021 | 0.968  ±0.006 | [[20.60 Â± 0.49, 0.40 Â± 0.49, 0.00 Â± 0.00, 0.00 Â± 0.00, 1.00 Â± 0.00],  [0.00 Â± 0.00, 9.60 Â± 0.49, 2.40 Â± 0.49, 0.40 Â± 0.49, 0.00 Â± 0.00],  [0.60 Â± 0.49, 1.20 Â± 0.98, 22.20 Â± 1.47, 4.60 Â± 0.49, 0.00 Â± 0.00],  [0.00 Â± 0.00, 0.40 Â± 0.49, 2.20 Â± 0.98, 50.40 Â± 0.49, 0.00 Â± 0.00],  [0.00 Â± 0.00, 0.00 Â± 0.00, 0.60 Â± 0.49, 0.40 Â± 0.49, 0.60 Â± 0.49]] |
| LOG-RQN-Z | 11079 | 133 | LR | 395.178 | 0.865  ±0.053 | 0.723  ±0.034 | 0.893  ±0.038 | 0.897  ±0.038 | 0.907  ±0.036 | 0.899  ±0.037 | 0.966  ±0.007 | [[21.00 Â± 0.00, 0.00 Â± 0.00, 0.00 Â± 0.00, 0.00 Â± 0.00, 0.40 Â± 0.49],  [0.00 Â± 0.00, 11.40 Â± 0.49, 1.00 Â± 0.00, 1.20 Â± 0.98, 0.00 Â± 0.00],  [1.00 Â± 0.00, 0.60 Â± 0.49, 22.40 Â± 0.49, 3.00 Â± 0.00, 0.00 Â± 0.00],  [0.00 Â± 0.00, 0.00 Â± 0.00, 3.40 Â± 1.96, 50.60 Â± 1.96, 0.00 Â± 0.00],  [1.00 Â± 0.00, 0.00 Â± 0.00, 0.60 Â± 0.49, 0.40 Â± 0.49, 0.00 Â± 0.00]] |
| LOG-NICG-Z | 11447 | 11 | LR | 126.936 | 0.502  ±0.121 | 0.639  ±0.158 | 0.714  ±0.087 | 0.621  ±0.127 | 0.681  ±0.072 | 0.602  ±0.073 | 0.903  ±0.019 | [[20.40 Â± 1.96, 0.80 Â± 0.98, 0.00 Â± 0.00, 0.60 Â± 0.49, 0.40 Â± 0.49],  [2.00 Â± 0.00, 12.00 Â± 0.00, 0.00 Â± 0.00, 0.00 Â± 0.00, 0.60 Â± 0.49],  [4.60 Â± 0.49, 3.40 Â± 1.96, 14.20 Â± 3.92, 3.80 Â± 0.98, 1.20 Â± 0.98],  [0.00 Â± 0.00, 0.80 Â± 0.98, 0.60 Â± 0.49, 49.40 Â± 0.49, 1.80 Â± 1.47],  [1.00 Â± 0.00, 0.00 Â± 0.00, 0.00 Â± 0.00, 0.40 Â± 0.49, 0.00 Â± 0.00]] |
| LOG-NPN-Z | 12690 | 133 | MLP | 200.291 | 0.409  ±0.007 | 0.589  ±0.035 | 0.690  ±0.032 | 0.665  ±0.127 | 0.607  ±0.028 | 0.548  ±0.019 | 0.860  ±0.029 | [[19.20 Â± 1.47, 0.00 Â± 0.00, 0.00 Â± 0.00, 0.80 Â± 0.98, 1.80 Â± 1.47],  [0.00 Â± 0.00, 11.20 Â± 0.98, 0.00 Â± 0.00, 2.00 Â± 0.00, 1.40 Â± 0.49],  [0.00 Â± 0.00, 0.00 Â± 0.00, 2.00 Â± 0.00, 23.00 Â± 0.00, 2.80 Â± 1.47],  [0.00 Â± 0.00, 0.00 Â± 0.00, 0.00 Â± 0.00, 48.40 Â± 0.49, 3.40 Â± 0.49],  [0.40 Â± 0.49, 0.00 Â± 0.00, 0.00 Â± 0.00, 1.00 Â± 0.20, 0.60 Â± 0.49]] |
| LOG-RQN | 12934 | 11 | MLP | 399.033 | 0.850  ±0.027 | 0.771  ±0.084 | 0.891  ±0.017 | 0.891  ±0.024 | 0.897  ±0.018 | 0.892  ±0.022 | 0.952  ±0.004 | [[19.60 Â± 0.49, 0.80 Â± 0.98, 0.00 Â± 0.00, 1.00 Â± 0.00, 0.00 Â± 0.00],  [0.00 Â± 0.00, 10.80 Â± 1.47, 2.80 Â± 0.98, 0.00 Â± 0.00, 0.00 Â± 0.00],  [1.00 Â± 0.00, 1.20 Â± 0.98, 22.60 Â± 1.96, 4.60 Â± 0.49, 0.00 Â± 0.00],  [0.00 Â± 0.00, 0.00 Â± 0.00, 1.20 Â± 0.98, 50.40 Â± 0.49, 0.00 Â± 0.00],  [0.40 Â± 0.49, 0.00 Â± 0.00, 0.60 Â± 0.49, 0.40 Â± 0.49, 0.60 Â± 0.49]] |
| LOG-RQN-Z | 12934 | 253 | MLP | 405.492 | 0.875  ±0.043 | 0.752  ±0.049 | 0.914  ±0.030 | 0.906  ±0.020 | 0.914  ±0.028 | 0.909  ±0.025 | 0.968  ±0.010 | [[19.80 Â± 0.42, 0.80 Â± 0.98, 0.00 Â± 0.00, 0.80 Â± 0.20, 0.00 Â± 0.00],  [0.00 Â± 0.00, 10.80 Â± 1.47, 2.80 Â± 0.98, 0.00 Â± 0.00, 0.00 Â± 0.00],  [1.00 Â± 0.00, 1.20 Â± 0.98, 22.60 Â± 1.96, 4.60 Â± 0.49, 0.00 Â± 0.00],  [0.00 Â± 0.00, 0.00 Â± 0.00, 1.20 Â± 0.98, 50.40 Â± 0.49, 0.00 Â± 0.00],  [0.40 Â± 0.49, 0.00 Â± 0.00, 0.60 Â± 0.49, 0.40 Â± 0.49, 0.60 Â± 0.49]] |
| LOG-NICG-Z | 11079 | 49 | MLP | 128.561 | 0.435  ±0.092 | 0.629  ±0.099 | 0.583  ±0.121 | 0.583  ±0.097 | 0.598  ±0.110 | 0.550  ±0.047 | 0.855  ±0.017 | [[13.40 Â± 2.94, 0.00 Â± 0.00, 0.00 Â± 0.00, 0.00 Â± 0.00, 9.60 Â± 2.94],  [0.00 Â± 0.00, 11.20 Â± 0.98, 0.00 Â± 0.00, 0.00 Â± 0.00, 2.60 Â± 1.96],  [0.00 Â± 0.00, 2.80 Â± 1.47, 7.40 Â± 4.41, 14.20 Â± 1.47, 4.00 Â± 2.45],  [0.00 Â± 0.00, 0.40 Â± 0.49, 1.80 Â± 1.47, 35.20 Â± 1.47, 13.40 Â± 0.49],  [0.00 Â± 0.00, 0.00 Â± 0.00, 0.00 Â± 0.00, 0.40 Â± 0.49, 1.60 Â± 0.49]] |
| LOG-NPN-Z | 10523 | 94 | XGB | 92.506 | 0.359  ±0.085 | 0.507  ±0.073 | 0.592  ±0.092 | 0.481  ±0.012 | 0.568  ±0.081 | 0.497  ±0.060 | 0.728  ±0.047 | [[17.60 Â± 0.49, 1.80 Â± 1.47, 0.00 Â± 0.00, 0.00 Â± 0.00, 1.60 Â± 1.96],  [0.00 Â± 0.00, 7.80 Â± 0.98, 0.00 Â± 0.00, 3.00 Â± 2.45, 1.60 Â± 1.96],  [0.00 Â± 0.00, 3.40 Â± 1.96, 0.80 Â± 0.98, 25.40 Â± 1.96, 1.60 Â± 1.96],  [0.00 Â± 0.00, 1.80 Â± 1.47, 0.00 Â± 0.00, 43.20 Â± 5.88, 6.40 Â± 7.84],  [1.00 Â± 0.00, 0.00 Â± 0.00, 0.00 Â± 0.00, 0.60 Â± 0.49, 0.40 Â± 0.49]] |
| LOG-RQN | 12690 | 11 | XGB | 237.678 | 0.724  ±0.071 | 0.728  ±0.155 | 0.864  ±0.046 | 0.815  ±0.058 | 0.812  ±0.046 | 0.805  ±0.052 | 0.927  ±0.020 | [[20.60 Â± 1.96, 0.40 Â± 0.49, 0.00 Â± 0.00, 0.00 Â± 0.00, 0.60 Â± 0.49],  [0.60 Â± 0.49, 10.20 Â± 1.47, 2.00 Â± 0.00, 0.00 Â± 0.00, 0.00 Â± 0.00],  [1.80 Â± 1.47, 1.60 Â± 0.49, 20.20 Â± 1.47, 5.00 Â± 0.00, 0.00 Â± 0.00],  [0.40 Â± 0.49, 0.40 Â± 0.49, 1.80 Â± 1.47, 50.40 Â± 1.96, 0.00 Â± 0.00],  [0.40 Â± 0.49, 0.00 Â± 0.00, 0.60 Â± 0.49, 0.40 Â± 0.49, 0.60 Â± 0.49]] |
| LOG-RQN-Z | 10756 | 11 | XGB | 300.160 | 0.787  ±0.054 | 0.713  ±0.088 | 0.875  ±0.032 | 0.849  ±0.042 | 0.854  ±0.038 | 0.845  ±0.044 | 0.937  ±0.028 | [[20.20 Â± 0.98, 0.00 Â± 0.00, 0.00 Â± 0.00, 0.40 Â± 0.49, 0.60 Â± 0.49],  [0.00 Â± 0.00, 11.80 Â± 0.98, 1.00 Â± 0.00, 0.00 Â± 0.00, 0.00 Â± 0.00],  [1.80 Â± 0.98, 1.20 Â± 0.98, 22.00 Â± 0.00, 4.00 Â± 0.00, 0.00 Â± 0.00],  [0.40 Â± 0.49, 0.80 Â± 0.98, 2.60 Â± 0.49, 49.20 Â± 0.98, 0.00 Â± 0.00],  [1.00 Â± 0.00, 0.00 Â± 0.00, 0.60 Â± 0.49, 0.40 Â± 0.49, 0.00 Â± 0.00]] |
| LOG-NICG-Z | 10936 | 94 | XGB | 123.406 | 0.428  ±0.064 | 0.553  ±0.085 | 0.638  ±0.042 | 0.514  0.042± | 0.619  ±0.049 | 0.551  ±0.045 | 0.797  ±0.037 | [[19.00 Â± 0.00, 0.40 Â± 0.49, 0.00 Â± 0.00, 1.20 Â± 0.98, 2.60 Â± 0.49],  [0.00 Â± 0.00, 9.00 Â± 0.00, 0.00 Â± 0.00, 3.60 Â± 1.96, 0.00 Â± 0.00],  [0.00 Â± 0.00, 0.00 Â± 0.00, 0.60 Â± 0.49, 25.80 Â± 0.98, 0.60 Â± 0.49],  [0.00 Â± 0.00, 0.00 Â± 0.00, 0.00 Â± 0.00, 52.80 Â± 1.47, 0.40 Â± 0.49],  [0.00 Â± 0.00, 0.00 Â± 0.00, 0.00 Â± 0.00, 1.60 Â± 0.49, 0.40 Â± 0.49]] |
