# Supplementary material for: Normalization and Selecting Non-Differentially Expressed Genes Improve Machine Learning Modelling of Cross-Platform Transcriptomic Data
Source: Trans Artif Intell. Author manuscript; Available in PMC 2025 Jul 8. (PMC12235674; doi:10.53941/tai.2025.100005)
Supplement: Supplementary [file NIHMS2087281-supplement-Supplementary.zip › Supplementary table 8.docx]

| Supplementary table 8. Average performance results (mean ± standard deviation) of the best-performing models (with the highest Kappa) on data constructed using DEG and NDEG genes selected via one-way ANOVA. (**Model-S**) | | | | | | | | | | | | |
| --- | --- | --- | --- | --- | --- | --- | --- | --- | --- | --- | --- | --- |
| Normalization _Method | DEG_ number | NDEG_ number | Model | E*_value_* | Kappa | Balanced _Accuracy | Accuracy | Precision | Recall | F1 | AUC | Confusion Matrix |
| LOG-NPN-Z | 11516 | 11 | SVM | 157.724 | 0.497  ±0.066 | 0.587  ±0.068 | 0.684  ±0.068 | 0.817  ±0.016 | 0.644  ±0.062 | 0.654  ±0.051 | 0.853  ±0.003 | [[15.80 Â± 1.45, 1.40 Â± 0.49, 0.00 Â± 0.00, 0.00 Â± 0.00, 5.60 Â± 0.40],  [0.60 Â± 0.20, 9.40 Â± 0.49, 0.20 Â± 0.45, 1.60 Â± 0.49, 0.00 Â± 0.00],  [0.40 Â± 0.20, 0.60 Â± 0.49, 23.60 Â± 1.96, 4.40 Â± 0.49, 0.00 Â± 0.00],  [0.00 Â± 0.00, 0.40 Â± 0.49, 0.60 Â± 0.49, 51.20 Â± 0.98, 1.00 Â± 0.00],  [0.00 Â± 0.00, 0.00 Â± 0.00, 0.60 Â± 0.49, 0.40 Â± 0.49, 1.00 Â± 0.00]] |
| LOG-RQN | 11282 | 11 | SVM | 356.050 | 0.849  ±0.053 | 0.702  ±0.048 | 0.873  ±0.031 | 0.887  ±0.037 | 0.897  ±0.034 | 0.889  ±0.039 | 0.962  ±0.007 | [[21.00 Â± 1.96, 0.40 Â± 0.49, 0.00 Â± 0.00, 0.40 Â± 0.49, 0.00 Â± 0.00],  [1.60 Â± 0.49, 10.00 Â± 0.00, 2.80 Â± 0.98, 0.00 Â± 0.00, 0.00 Â± 0.00],  [0.60 Â± 0.49, 1.80 Â± 1.47, 20.80 Â± 3.43, 0.20 Â± 0.98, 0.00 Â± 0.00],  [0.00 Â± 0.00, 0.60 Â± 0.49, 1.60 Â± 0.49, 53.40 Â± 0.49, 0.00 Â± 0.00],  [0.40 Â± 0.49, 0.60 Â± 0.49, 0.00 Â± 0.00, 1.00 Â± 0.00, 0.00 Â± 0.00]] |
| LOG-RQN-Z | 11366 | 11 | SVM | 340.511 | 0.829  ±0.056 | 0.689  ±0.046 | 0.871  ±0.011 | 0.869  ±0.010 | 0.883  ±0.010 | 0.874  ±0.012 | 0.953  ±0.003 | [[21.80 Â± 0.49, 0.00 Â± 0.00, 0.00 Â± 0.00, 0.00 Â± 0.00, 0.40 Â± 0.49],  [0.00 Â± 0.00, 10.40 Â± 0.49, 3.00 Â± 0.00, 1.20 Â± 0.98, 0.00 Â± 0.00],  [1.00 Â± 0.00, 0.60 Â± 0.49, 21.40 Â± 0.49, 3.00 Â± 0.00, 0.00 Â± 0.00],  [0.00 Â± 0.00, 0.00 Â± 0.00, 2.40 Â± 1.96, 50.60 Â± 1.96, 0.00 Â± 0.00],  [1.00 Â± 0.00, 0.00 Â± 0.00, 0.60 Â± 0.49, 0.40 Â± 0.49, 0.00 Â± 0.00]] |
| LOG-NICG-Z | 11516 | 94 | SVM | 89.519 | 0.441  ±0.186 | 0.626  ±0.210 | 0.791  ±0.011 | 0.627  ±0.105 | 0.632  ±0.094 | 0.565  ±0.141 | 0.815  ±0.058 | [[20.00 Â± 0.00, 0.00 Â± 0.00, 0.00 Â± 0.00, 0.40 Â± 0.49, 0.00 Â± 0.00],  [2.00 Â± 0.00, 6.00 Â± 0.00, 3.60 Â± 0.49, 2.40 Â± 0.49, 0.00 Â± 0.00],  [1.20 Â± 0.98, 0.60 Â± 0.49, 24.00 Â± 2.45, 4.80 Â± 1.47, 0.00 Â± 0.00],  [3.00 Â± 2.45, 0.00 Â± 0.00, 0.40 Â± 0.49, 48.00 Â± 2.45, 0.00 Â± 0.00],  [0.60 Â± 0.49, 0.00 Â± 0.00, 0.60 Â± 0.49, 0.40 Â± 0.49, 0.00 Â± 0.00]] |
| LOG-NPN-Z | 11516 | 94 | RF | 85.940 | 0.406  ±0.104 | 0.407  0.053 | 0.646  ±0.051 | 0.674  ±0.162 | 0.644  ±0.050 | 0.560  ±0.079 | 0.843  ±0.007 | [[8.40 Â± 0.49, 0.00 Â± 0.00, 1.60 Â± 0.49, 11.40 Â± 0.49, 0.00 Â± 0.00],  [0.40 Â± 0.49, 2.00 Â± 0.00, 4.40 Â± 0.49, 6.80 Â± 1.47, 0.00 Â± 0.00],  [1.60 Â± 0.49, 6.00 Â± 4.90, 15.20 Â± 3.92, 6.60 Â± 1.96, 0.00 Â± 0.00],  [0.40 Â± 0.49, 0.60 Â± 0.49, 0.00 Â± 0.00, 50.60 Â± 0.49, 0.00 Â± 0.00],  [1.00 Â± 0.00, 0.60 Â± 0.49, 0.00 Â± 0.00, 0.40 Â± 0.49, 0.00 Â± 0.00]] |
| LOG-RQN | 11183 | 11 | RF | 298.575 | 0.738  ±0.031 | 0.585  ±0.032 | 0.815  ±0.021 | 0.804  ±0.063 | 0.824  ±0.020 | 0.788  ±0.029 | 0.931  ±0.015 | [[18.20 Â± 1.47, 0.00 Â± 0.00, 0.00 Â± 0.00, 1.00 Â± 0.00, 0.00 Â± 0.00],  [0.40 Â± 0.49, 4.00 Â± 2.45, 5.20 Â± 3.92, 4.80 Â± 1.47, 0.00 Â± 0.00],  [1.00 Â± 0.00, 1.20 Â± 0.98, 22.00 Â± 2.45, 6.60 Â± 0.49, 0.00 Â± 0.00],  [0.00 Â± 0.00, 0.00 Â± 0.00, 0.00 Â± 0.00, 52.00 Â± 2.45, 0.00 Â± 0.00],  [0.40 Â± 0.49, 0.60 Â± 0.49, 0.00 Â± 0.00, 0.60 Â± 0.49, 0.00 Â± 0.00]] |
| LOG-RQN-Z | 10523 | 11 | RF | 362.542 | 0.741  ±0.019 | 0.571  ±0.010 | 0.819  ±0.015 | 0.776  ±0.037 | 0.827  ±0.012 | 0.783  ±0.017 | 0.936  ±0.006 | [[20.40 Â± 0.98, 0.40 Â± 0.49, 0.00 Â± 0.00, 0.40 Â± 0.49, 0.60 Â± 0.49],  [0.00 Â± 0.00, 4.40 Â± 0.49, 3.60 Â± 0.49, 5.00 Â± 0.00, 0.00 Â± 0.00],  [1.00 Â± 0.00, 0.00 Â± 0.00, 24.60 Â± 1.96, 3.60 Â± 1.96, 0.60 Â± 0.49],  [0.00 Â± 0.00, 2.00 Â± 0.00, 0.80 Â± 0.98, 46.00 Â± 2.45, 0.00 Â± 0.00],  [1.00 Â± 0.00, 0.00 Â± 0.00, 0.00 Â± 0.00, 0.80 Â± 0.98, 0.00 Â± 0.00]] |
| LOG-NICG-Z | 11516 | 133 | RF | 70.452 | 0.365  ±0.126 | 0.403  ±0.066 | 0.615  ±0.080 | 0.637  ±0.216 | 0.608  ±0.075 | 0.527  ±0.115 | 0.839  ±0.031 | [[12.80 Â± 1.96, 3.00 Â± 0.00, 0.00 Â± 0.00, 4.00 Â± 2.45, 1.20 Â± 0.98],  [0.00 Â± 0.00, 4.80 Â± 0.49, 0.00 Â± 0.00, 6.40 Â± 0.49, 1.00 Â± 0.00],  [0.00 Â± 0.00, 2.20 Â± 0.98, 0.00 Â± 0.00, 26.40 Â± 0.49, 0.00 Â± 0.00],  [0.00 Â± 0.00, 0.60 Â± 0.49, 0.00 Â± 0.00, 54.00 Â± 0.00, 0.00 Â± 0.00],  [0.60 Â± 0.49, 1.00 Â± 0.00, 0.00 Â± 0.00, 0.80 Â± 0.98, 0.00 Â± 0.00]] |
| LOG-NPN-Z | 11516 | 11 | LR | 237.174 | 0.554  ±0.021 | 0.547  ±0.019 | 0.710  ±0.013 | 0.809  ±0.007 | 0.715  ±0.011 | 0.651  ±0.014 | 0.911  ±0.010 | [[14.40 Â± 1.96, 0.40 Â± 0.49, 0.40 Â± 0.49, 0.00 Â± 0.00, 6.60 Â± 0.49],  [1.80 Â± 0.98, 8.60 Â± 0.49, 1.60 Â± 1.96, 0.00 Â± 0.00, 2.00 Â± 0.00],  [1.80 Â± 1.47, 0.40 Â± 1.96, 0.20 Â± 0.49, 28.20 Â± 0.98, 0.00 Â± 0.00],  [0.00 Â± 0.00, 0.00 Â± 0.00, 0.60 Â± 0.49, 48.00 Â± 2.45, 0.60 Â± 0.49],  [1.20 Â± 0.98, 0.00 Â± 0.00, 0.00 Â± 0.00, 0.00 Â± 0.00, 0.60 Â± 0.49]] |
| LOG-RQN | 10756 | 49 | LR | 398.764 | 0.877  ±0.039 | 0.731  ±0.051 | 0.906  ±0.022 | 0.911  ±0.021 | 0.915  ±0.026 | 0.910  ±0.023 | 0.965  ±0.034 | [[20.00 Â± 3.03, 0.00 Â± 0.00, 0.00 Â± 0.00, 0.20 Â± 0.40, 1.40 Â± 2.80],  [0.80 Â± 0.40, 11.80 Â± 1.47, 0.40 Â± 0.49, 0.60 Â± 1.20, 0.40 Â± 0.80],  [0.20 Â± 0.40, 3.60 Â± 1.20, 18.40 Â± 9.29, 6.80 Â± 10.61, 0.00 Â± 0.00],  [0.00 Â± 0.00, 1.00 Â± 0.63, 0.60 Â± 0.49, 49.60 Â± 1.85, 0.20 Â± 0.40],  [0.80 Â± 0.40, 0.00 Â± 0.00, 0.00 Â± 0.00, 1.00 Â± 0.00, 0.20 Â± 0.40]] |
| LOG-RQN-Z | 10756 | 49 | LR | 389.692 | 0.880  ±0.058 | 0.730  ±0.040 | 0.909  ±0.045 | 0.906  ±0.042 | 0.917  ±0.041 | 0.909  ±0.041 | 0.951  ±0.004 | [[22.20 Â± 0.98, 0.00 Â± 0.00, 0.00 Â± 0.00, 0.00 Â± 0.00, 0.00 Â± 0.00],  [1.40 Â± 0.49, 11.20 Â± 1.17, 0.00 Â± 0.00, 0.00 Â± 0.00, 0.00 Â± 0.00],  [1.40 Â± 0.80, 6.20 Â± 0.75, 20.00 Â± 0.89, 1.00 Â± 0.89, 0.00 Â± 0.00],  [0.00 Â± 0.00, 2.00 Â± 0.63, 1.80 Â± 0.40, 49.00 Â± 1.90, 0.00 Â± 0.00],  [0.80 Â± 0.40, 0.00 Â± 0.00, 0.00 Â± 0.00, 1.00 Â± 0.00, 0.00 Â± 0.00]] |
| LOG-NICG-Z | 12294 | 253 | LR | 173.117 | 0.521  ±0.044 | 0.543  ±0.050 | 0.664  ±0.033 | 0.809  ±0.014 | 0.686  ±0.039 | 0.625  ±0.011 | 0.909  0.012 | [[10.00 Â± 2.00, 0.00 Â± 0.00, 0.00 Â± 0.00, 1.40 Â± 1.20, 9.40 Â± 2.33],  [0.00 Â± 0.00, 8.00 Â± 2.10, 0.00 Â± 0.00, 3.20 Â± 0.40, 2.60 Â± 2.80],  [0.00 Â± 0.00, 2.00 Â± 0.89, 0.00 Â± 0.00, 26.00 Â± 1.55, 1.20 Â± 1.47],  [0.00 Â± 0.00, 0.00 Â± 0.00, 0.00 Â± 0.00, 49.60 Â± 3.88, 2.60 Â± 2.80],  [0.00 Â± 0.00, 0.00 Â± 0.00, 0.00 Â± 0.00, 0.60 Â± 0.49, 1.40 Â± 0.49]] |
| LOG-NPN-Z | 12001 | 11 | MLP | 179.154 | 0.541  ±0.035 | 0.520  ±0.049 | 0.661  ±0.017 | 0.791  ±0.015 | 0.697  ±0.046 | 0.654  ±0.021 | 0.885  ±0.036 | [[11.80 Â± 0.49, 0.00 Â± 0.00, 0.00 Â± 0.00, 1.20 Â± 0.98, 8.00 Â± 0.20],  [0.00 Â± 0.00, 8.40 Â± 2.80, 0.00 Â± 0.00, 3.80 Â± 0.75, 1.80 Â± 1.47],  [0.00 Â± 0.00, 1.40 Â± 1.20, 0.40 Â± 0.49, 26.40 Â± 1.85, 1.00 Â± 0.89],  [0.00 Â± 0.00, 0.40 Â± 0.49, 0.00 Â± 0.00, 48.20 Â± 0.98, 3.20 Â± 0.49],  [0.00 Â± 0.00, 0.00 Â± 0.00, 0.00 Â± 0.00, 1.00 Â± 0.89, 1.00 Â± 0.49]] |
| LOG-RQN | 10756 | 133 | MLP | 372.258 | 0.883  ±0.074 | 0.739  ±0.045 | 0.911  ±0.052 | 0.907  ±0.048 | 0.920  ±0.050 | 0.912  ±0.050 | 0.956  ±0.008 | [[22.00 Â± 1.26, 0.00 Â± 0.00, 0.00 Â± 0.00, 0.00 Â± 0.00, 0.00 Â± 0.00],  [0.80 Â± 0.40, 12.40 Â± 1.02, 0.60 Â± 0.49, 0.00 Â± 0.00, 0.00 Â± 0.00],  [0.00 Â± 0.00, 3.60 Â± 1.02, 24.20 Â± 1.72, 1.40 Â± 0.49, 0.00 Â± 0.00],  [0.00 Â± 0.00, 0.40 Â± 0.49, 1.20 Â± 0.40, 49.60 Â± 1.85, 0.00 Â± 0.00],  [0.80 Â± 0.40, 0.00 Â± 0.00, 0.00 Â± 0.00, 1.00 Â± 0.00, 0.00 Â± 0.00]] |
| LOG-RQN-Z | 12934 | 253 | MLP | 405.492 | 0.875  ±0.043 | 0.752  ±0.049 | 0.914  ±0.030 | 0.906  ±0.020 | 0.914  ±0.028 | 0.909  ±0.025 | 0.968  ±0.010 | [[19.60 Â± 0.49, 0.80 Â± 0.98, 0.00 Â± 0.00, 1.00 Â± 0.00, 0.00 Â± 0.00],  [0.00 Â± 0.00, 10.80 Â± 1.47, 2.80 Â± 0.98, 0.00 Â± 0.00, 0.00 Â± 0.00],  [1.00 Â± 0.00, 1.20 Â± 0.98, 22.60 Â± 1.96, 4.60 Â± 0.49, 0.00 Â± 0.00],  [0.00 Â± 0.00, 0.00 Â± 0.00, 1.20 Â± 0.98, 50.40 Â± 0.49, 0.00 Â± 0.00],  [0.40 Â± 0.49, 0.00 Â± 0.00, 0.60 Â± 0.49, 0.40 Â± 0.49, 0.60 Â± 0.49]] |
| LOG-NICG-Z | 12001 | 11 | MLP | 138.324 | 0.508  ±0.078 | 0.506  ±0.059 | 0.671  ±0.034 | 0.749  ±0.051 | 0.676  ±0.033 | 0.608  ±0.046 | 0.901  ±0.029 | [[12.80 Â± 3.87, 0.00 Â± 0.00, 0.00 Â± 0.00, 4.60 Â± 2.94, 6.00 Â± 1.67],  [0.00 Â± 0.00, 3.00 Â± 1.26, 0.00 Â± 0.00, 9.80 Â± 0.75, 0.40 Â± 0.49],  [0.00 Â± 0.00, 0.00 Â± 0.00, 0.60 Â± 0.49, 26.80 Â± 0.75, 2.00 Â± 1.10],  [0.00 Â± 0.00, 0.00 Â± 0.00, 0.00 Â± 0.00, 46.80 Â± 4.92, 3.20 Â± 3.31],  [0.00 Â± 0.00, 0.00 Â± 0.00, 0.00 Â± 0.00, 0.40 Â± 0.49, 1.60 Â± 0.49]] |
| LOG-NPN-Z | 11447 | 133 | XGB | 109.841 | 0.465  ±0.135 | 0.502  ±0.067 | 0.619  ±0.148 | 0.713  ±0.126 | 0.627  ±0.144 | 0.569  ±0.074 | 0.774  ±0.045 | [[11.40 Â± 3.50, 0.00 Â± 0.00, 0.00 Â± 0.00, 4.20 Â± 1.94, 6.20 Â± 3.19],  [0.00 Â± 0.00, 3.80 Â± 0.98, 0.00 Â± 0.00, 9.60 Â± 1.36, 0.80 Â± 1.17],  [0.00 Â± 0.00, 0.00 Â± 0.00, 0.60 Â± 0.49, 25.80 Â± 1.17, 1.60 Â± 1.74],  [0.00 Â± 0.00, 0.00 Â± 0.00, 0.00 Â± 0.00, 47.20 Â± 5.38, 5.20 Â± 5.15],  [0.00 Â± 0.00, 0.00 Â± 0.00, 0.00 Â± 0.00, 0.40 Â± 0.49, 1.20 Â± 0.40]] |
| LOG-RQN | 10936 | 11 | XGB | 326.894 | 0.767  ±0.038 | 0.649  ±0.037 | 0.839  ±0.025 | 0.831  ±0.030 | 0.838  ±0.027 | 0.830  ±0.026 | 0.951  ±0.009 | [[20.60 Â± 1.02, 0.00 Â± 0.00, 0.00 Â± 0.00, 0.40 Â± 0.49, 0.00 Â± 0.00],  [0.00 Â± 0.00, 6.80 Â± 0.98, 6.40 Â± 1.02, 0.60 Â± 0.80, 0.00 Â± 0.00],  [0.60 Â± 0.49, 1.20 Â± 1.47, 24.00 Â± 2.19, 3.00 Â± 0.89, 0.40 Â± 0.49],  [0.00 Â± 0.00, 0.40 Â± 1.02, 0.80 Â± 0.75, 49.60 Â± 1.96, 0.40 Â± 0.80],  [1.40 Â± 0.80, 0.00 Â± 0.00, 0.20 Â± 0.40, 0.20 Â± 0.40, 0.00 Â± 0.00]] |
| LOG-RQN-Z | 10523 | 11 | XGB | 280.861 | 0.794  ±0.065 | 0.671  ±0.079 | 0.827  ±0.048 | 0.861  ±0.062 | 0.861  ±0.042 | 0.848  ±0.053 | 0.929  ±0.012 | [[20.00 Â± 0.63, 0.00 Â± 0.00, 0.00 Â± 0.00, 0.80 Â± 0.75, 0.00 Â± 0.00],  [0.00 Â± 0.00, 8.40 Â± 1.02, 5.00 Â± 0.63, 0.00 Â± 0.00, 0.00 Â± 0.00],  [0.80 Â± 0.40, 0.60 Â± 0.49, 24.80 Â± 0.75, 3.60 Â± 0.49, 0.00 Â± 0.00],  [0.00 Â± 0.00, 2.00 Â± 0.63, 1.00 Â± 0.63, 48.60 Â± 1.02, 0.40 Â± 0.80],  [2.00 Â± 0.00, 0.00 Â± 0.00, 0.00 Â± 0.00, 0.00 Â± 0.00, 0.00 Â± 0.00]] |
| LOG-NICG-Z | 10936 | 11 | XGB | 152.373 | 0.478  ±0.084 | 0.517  ±0.025 | 0.652  ±0.051 | 0.527  ±0.013 | 0.663  ±0.058 | 0.573  ±0.045 | 0.796  ±0.047 | [[10.00 Â± 3.41, 0.00 Â± 0.00, 0.00 Â± 0.00, 3.00 Â± 3.35, 8.20 Â± 1.94],  [0.00 Â± 0.00, 3.40Â± 1.02, 0.00 Â± 0.00, 9.60 Â± 1.02, 1.00 Â± 0.98],  [0.00 Â± 0.00, 0.00 Â± 0.00, 0.80 Â± 0.40, 25.00 Â± 2.00, 2.80 Â± 0.75],  [0.00 Â± 0.00, 0.00 Â± 0.00, 0.00 Â± 0.00, 44.20 Â± 4.58, 8.20 Â± 0.49],  [0.00 Â± 0.00, 0.00 Â± 0.00, 0.00 Â± 0.00, 0.00 Â± 0.00, 1.80 Â± 0.40]] |
