# Supplementary material for: Normalization and Selecting Non-Differentially Expressed Genes Improve Machine Learning Modelling of Cross-Platform Transcriptomic Data
Source: Trans Artif Intell. Author manuscript; Available in PMC 2025 Jul 8. (PMC12235674; doi:10.53941/tai.2025.100005)
Supplement: Supplementary [file NIHMS2087281-supplement-Supplementary.zip › Supplementary table 9.docx]

| Supplementary table 9. Average performance results (mean ± standard deviation) of the best-performing models (with the highest Balanced Accuracy) on data constructed using DEG and NDEG genes selected via one-way ANOVA. (**Model-A**) | | | | | | | | | | | | |
| --- | --- | --- | --- | --- | --- | --- | --- | --- | --- | --- | --- | --- |
| Normalization _Method | DEG_ number | NDEG_ number | Model | E*_value_* | Kappa | Balanced _Accuracy | Accuracy | Precision | Recall | F1 | AUC | Confusion Matrix |
| LOG-NPN-Z | 13155 | 22 | SVM | 69.623 | 0.308  ±0.093 | 0.478  ±0.095 | 0.495  ±0.072 | 0.333  ±0.042 | 0.461  ±0.068 | 0.344  ±0.064 | 0.802  ±0.085 | [[19.60 Â± 0.63, 0.40 Â± 0.49, 1.00 Â± 0.00, 0.00 Â± 0.00, 0.00 Â± 0.00],  [0.40 Â± 0.49, 9.60 Â± 3.01, 1.40 Â± 1.20, 1.80 Â± 2.23, 0.00 Â± 0.00],  [0.40 Â± 0.49, 8.20 Â± 8.45, 8.20 Â± 5.42, 12.20 Â± 4.45, 0.00 Â± 0.00],  [0.40 Â± 0.49, 7.80 Â± 10.46, 1.40 Â± 1.50, 43.20 Â± 8.47, 0.00 Â± 0.00],  [0.20 Â± 0.40, 0.40 Â± 0.49, 0.20 Â± 0.40, 0.80 Â± 0.75, 0.00 Â± 0.00]] |
| LOG-RQN | 12621 | 6 | SVM | 147.756 | 0.581  ±0.128 | 0.615  ±0.125 | 0.718  ±112 | 0.748  ±0.053 | 0.703  ±0.102 | 0.697  ±0.095 | 0.901  ±0.041 | [[20.60 Â± 1.02, 0.00 Â± 0.00, 0.00 Â± 0.00, 0.60 Â± 0.80, 0.00 Â± 0.00],  [0.60 Â± 0.49, 9.20 Â± 0.75, 1.20 Â± 0.75, 2.40 Â± 1.02, 0.00 Â± 0.00],  [0.20 Â± 0.40, 1.00 Â± 1.26, 13.40 Â± 3.01, 14.80 Â± 3.31, 0.00 Â± 0.00],  [0.60 Â± 0.49, 0.20 Â± 0.40, 1.40 Â± 0.80, 49.80 Â± 2.86, 0.00 Â± 0.00],  [0.60 Â± 0.49, 0.00 Â± 0.00, 0.40 Â± 0.49, 1.00 Â± 0.63, 0.00 Â± 0.00]] |
| LOG-RQN-Z | 11790 | 97 | SVM | 140.260 | 0.471  ±0.098 | 0.628  ±0.089 | 0.678  ±0.102 | 0.748  ±0.051 | 0.604  ±0.096 | 0.582  ±0.079 | 0.881  ±0.042 | [[20.80 Â± 0.98, 0.00 Â± 0.00, 0.00 Â± 0.00, 0.60 Â± 0.80, 0.20 Â± 0.40],  [0.40 Â± 0.49, 9.60 Â± 2.15, 1.40 Â± 1.02, 2.00 Â± 1.41, 0.00 Â± 0.00],  [0.20 Â± 0.40, 2.80 Â± 2.99, 12.20 Â± 3.54, 14.00 Â± 3.29, 0.20 Â± 0.40],  [0.60 Â± 0.49, 0.80 Â± 1.17, 1.60 Â± 1.36, 49.00 Â± 1.41, 0.00 Â± 0.00],  [0.40 Â± 0.49, 0.00 Â± 0.00, 0.20 Â± 0.40, 0.80 Â± 0.40, 0.20 Â± 0.40]] |
| LOG-NICG-Z | 13155 | 22 | SVM | 66.822 | 0.337  ±0.135 | 0.479  ±0.118 | 0.515  ±0.115 | 0.539  ±0.285 | 0.488  ±0.104 | 0.388  ±0.143 | 0.812  ±0.119 | [[19.90 Â± 0.63, 0.40 Â± 0.49, 0.80 Â± 0.00, 0.00 Â± 0.00, 0.00 Â± 0.00],  [0.40 Â± 0.49, 9.60 Â± 3.01, 1.40 Â± 1.20, 1.80 Â± 2.23, 0.00 Â± 0.00],  [0.40 Â± 0.49, 8.20 Â± 8.45, 8.20 Â± 5.42, 12.20 Â± 4.45, 0.00 Â± 0.00],  [0.40 Â± 0.49, 7.80 Â± 10.46, 1.40 Â± 1.50, 43.20 Â± 8.47, 0.00 Â± 0.00],  [0.20 Â± 0.40, 0.40 Â± 0.49, 0.20 Â± 0.40, 0.80 Â± 0.75, 0.00 Â± 0.00]] |
| LOG-NPN-Z | 12427 | 6 | RF | 101.295 | 0.394  ±0.058 | 0.440  ±0.050 | 0.554  ±0.052 | 0.645  ±0.057 | 0.590  ±0.049 | 0.563  ±0.066 | 0.821  ±0.028 | [[18.50 Â± 0.43, 0.40 Â± 0.49, 1.40 Â± 0.00, 0.00 Â± 0.00, 0.00 Â± 0.00],  [0.40 Â± 0.29, 9.40 Â± 3.01, 1.20 Â± 1.20, 1.80 Â± 2.23, 0.00 Â± 0.00],  [0.40 Â± 0.49, 8.20 Â± 8.45, 8.20 Â± 5.12, 12.20 Â± 4.45, 0.00 Â± 0.00],  [0.40 Â± 0.49, 7.80 Â± 10.46, 1.40 Â± 1.50, 41.20 Â± 8.17, 0.00 Â± 0.00],  [0.20 Â± 0.40, 0.40 Â± 0.49, 0.20 Â± 0.41, 0.80 Â± 0.75, 0.00 Â± 0.00]] |
| LOG-RQN | 14197 | 6 | RF | 125.817 | 0.423  ±0.044 | 0.444  ±0.028 | 0.591  ±0.028 | 0.597  ±0.022 | 0.609  ±0.024 | 0.594  ±0.027 | 0.771  ±0.037 | [[18.70 Â± 0.21, 0.40 Â± 0.49, 1.40 Â± 0.00, 0.00 Â± 0.00, 0.00 Â± 0.00],  [0.40 Â± 0.49, 9.40 Â± 3.05, 1.20 Â± 1.20, 1.80 Â± 2.23, 0.00 Â± 0.00],  [0.40 Â± 0.49, 8.20 Â± 8.45, 8.20 Â± 3.41, 12.20 Â± 4.45, 0.00 Â± 0.00],  [0.40 Â± 0.49, 7.80 Â± 7.46, 1.20 Â± 1.50, 41.20 Â± 8.43, 0.00 Â± 0.00],  [0.20 Â± 0.40, 0.40 Â± 0.49, 0.20 Â± 0.40, 0.80 Â± 0.75, 0.00 Â± 0.00]] |
| LOG-RQN-Z | 13744 | 6 | RF | 133.644 | 0.432  ±0.047 | 0.447  ±0.021 | 0.632  ±0.052 | 0.613  ±0.045 | 0.620  ±0.041 | 0.603  ±0.045 | 0.790  ±0.038 | [[19.60 Â± 0.55, 0.40 Â± 0.49, 1.40 Â± 0.00, 0.00 Â± 0.00, 0.00 Â± 0.00],  [0.40 Â± 0.49, 9.40 Â± 3.05, 1.20 Â± 1.20, 1.80 Â± 2.23, 0.00 Â± 0.00],  [0.40 Â± 0.49, 8.20 Â± 8.45, 8.20 Â± 3.41, 12.20 Â± 4.45, 0.00 Â± 0.00],  [0.40 Â± 0.49, 7.80 Â± 7.46, 1.20 Â± 1.50, 41.20 Â± 8.43, 0.00 Â± 0.00],  [0.20 Â± 0.40, 0.40 Â± 0.49, 0.20 Â± 0.40, 0.80 Â± 0.75, 0.00 Â± 0.00]] |
| LOG-NICG-Z | 11408 | 22 | RF | 34.787 | 0.253  ±0.193 | 0.383  ±0.143 | 0.498  ±0.139 | 0.513  ±0.225 | 0.489  ±0.135 | 0.434  ±0.182 | 0772  ±0.042 | [[17.60 Â± 2.04, 0.00 Â± 0.00, 3.80 Â± 1.04, 0.00 Â± 0.00, 0.00 Â± 0.00],  [0.20 Â± 0.40, 0.00 Â± 0.00, 13.60 Â± 1.02, 0.00 Â± 0.00, 0.00 Â± 0.00],  [0.00 Â± 0.00, 0.00 Â± 0.00, 29.00 Â± 1.41, 0.00 Â± 0.00, 0.00 Â± 0.00],  [0.60 Â± 0.49, 0.00 Â± 0.00, 50.40 Â± 2.61, 0.00 Â± 0.00, 0.00 Â± 0.00],  [0.20 Â± 0.40, 0.00 Â± 0.00, 1.80 Â± 0.40, 0.00 Â± 0.00, 0.00 Â± 0.00]] |
| LOG-NPN-Z | 12303 | 97 | LR | 46.588 | 0.208  ±0.052 | 0.367  ±0.043 | 0.386  ±0.033 | 0.243  ±0.007 | 0.392  ±0.039 | 0.266  ±0.031 | 0.870  ±0.032 | [[17.60 Â± 2.24, 0.00 Â± 0.00, 3.60 Â± 3.14, 0.00 Â± 0.00, 0.00 Â± 0.00],  [0.20 Â± 0.40, 0.00 Â± 0.00, 13.60 Â± 1.02, 0.00 Â± 0.00, 0.00 Â± 0.00],  [0.00 Â± 0.00, 0.00 Â± 0.00, 28.00 Â± 1.41, 0.00 Â± 0.00, 0.00 Â± 0.00],  [0.60 Â± 0.49, 0.00 Â± 0.00, 52.40 Â± 2.06, 0.00 Â± 0.00, 0.00 Â± 0.00],  [0.20 Â± 0.40, 0.00 Â± 0.00, 1.80 Â± 0.40, 0.00 Â± 0.00, 0.00 Â± 0.00]] |
| LOG-RQN | 11408 | 97 | LR | 251.803 | 0.660  ±0.075 | 0.642  ±0.035 | 0.747  ±0.051 | 0.780  ±0.058 | 0.771  ±0.054 | 0.748  ±0.064 | 0.915  ±0.017 | [[20.20 Â± 1.17, 0.00 Â± 0.00, 0.00 Â± 0.00, 0.40 Â± 0.49, 0.20 Â± 0.40],  [0.40 Â± 0.49, 11.20 Â± 2.32, 1.20 Â± 1.17, 1.20 Â± 1.17, 0.00 Â± 0.00],  [0.20 Â± 0.40, 6.00 Â± 6.03, 10.80 Â± 7.76, 12.60 Â± 2.42, 0.00 Â± 0.00],  [0.40 Â± 0.49, 4.40 Â± 4.59, 1.40 Â± 1.50, 45.80 Â± 3.37, 0.00 Â± 0.00],  [0.40 Â± 0.49, 0.60 Â± 0.80, 0.20 Â± 0.40, 0.20 Â± 0.40, 0.20 Â± 0.40]] |
| LOG-RQN-Z | 11790 | 97 | LR | 239.655 | 0.616  ±0.051 | 0.660  ±0.054 | 0.753  ±0.042 | 0.764  ±0.044 | 0.742  ±0.035 | 0.704  ±0.051 | 0.917  ±0.012 | [[21.00 Â± 1.10, 0.00 Â± 0.00, 0.00 Â± 0.00, 0.40 Â± 0.49, 0.40 Â± 0.49],  [0.60 Â± 0.49, 10.40 Â± 1.85, 1.00 Â± 0.63, 1.60 Â± 1.02, 0.00 Â± 0.00],  [0.20 Â± 0.40, 2.00 Â± 1.67, 16.80 Â± 3.87, 10.20 Â± 3.82, 0.40 Â± 0.49],  [0.60 Â± 0.49, 1.20 Â± 0.40, 2.60 Â± 1.36, 46.40 Â± 0.49, 0.20 Â± 0.40],  [0.60 Â± 0.49, 0.00 Â± 0.00, 0.60 Â± 0.49, 0.60 Â± 0.49, 0.20 Â± 0.40]] |
| LOG-NICG-Z | 12189 | 22 | LR | 47.318 | 0.216  ±0.057 | 0.363  ±0.042 | 0.426  ±0.042 | 0.251  ±0.007 | 0.406  ±0.048 | 0.278  ±0.037 | 0.889  ±0.009 | [[17.60 Â± 3.44, 0.00 Â± 0.00, 4.00 Â± 3.16, 0.00 Â± 0.00, 0.00 Â± 0.00],  [0.20 Â± 0.40, 0.00 Â± 0.00, 13.60 Â± 0.49, 0.00 Â± 0.00, 0.00 Â± 0.00],  [0.00 Â± 0.00, 0.00 Â± 0.00, 29.20 Â± 1.17, 0.00 Â± 0.00, 0.00 Â± 0.00],  [0.60 Â± 0.49, 0.00 Â± 0.00, 50.80 Â± 1.60, 0.00 Â± 0.00, 0.00 Â± 0.00],  [0.20 Â± 0.40, 0.00 Â± 0.00, 1.80 Â± 0.40, 0.00 Â± 0.00, 0.00 Â± 0.00]] |
| LOG-NPN-Z | 11408 | 6 | MLP | 73.820 | 0.297  ±0.063 | 0.463  ±0.074 | 0.491  ±0.041 | 0.306  ±0.048 | 0.453  ±0.046 | 0.336  ±0.049 | 0.858  ±0.014 | [[19.20 Â± 0.63, 0.40 Â± 0.43, 1.00 Â± 0.00, 0.00 Â± 0.00, 0.00 Â± 0.00],  [0.40 Â± 0.49, 9.60 Â± 1.99, 1.40 Â± 1.20, 1.80 Â± 2.23, 0.00 Â± 0.00],  [0.40 Â± 0.49, 8.20 Â± 6.41, 8.40 Â± 4.12, 12.20 Â± 4.45, 0.00 Â± 0.00],  [0.40 Â± 0.49, 7.80 Â± 10.46, 1.40 Â± 1.50, 43.20 Â±5.41, 0.00 Â± 0.00],  [0.20 Â± 0.40, 0.40 Â± 0.49, 0.20 Â± 0.40, 0.80 Â± 0.75, 0.00 Â± 0.00]] |
| LOG-RQN | 12621 | 6 | MLP | 258.298 | 0.713  ±0.058 | 0.718  ±0.111 | 0.800  ±0.039 | 0.807  ±0.037 | 0.805  ±0.039 | 0.794  ±0.042 | 0.925  ±0.012 | [[20.80 Â± 1.47, 0.00 Â± 0.00, 0.00 Â± 0.00, 0.40 Â± 0.49, 0.40 Â± 0.49],  [0.80 Â± 0.75, 10.40 Â± 1.02, 1.40 Â± 0.80, 0.80 Â± 0.98, 0.00 Â± 0.00],  [0.20 Â± 0.40, 2.00 Â± 1.26, 15.40 Â± 2.65, 10.40 Â± 3.38, 0.20 Â± 0.40],  [0.60 Â± 0.49, 1.80 Â± 1.17, 2.80 Â± 1.33, 47.20 Â± 0.75, 0.60 Â± 0.80],  [0.00 Â± 0.00, 0.00 Â± 0.00, 0.80 Â± 0.40, 0.40 Â± 0.49, 0.60 Â± 0.49]] |
| LOG-RQN-Z | 12621 | 22 | MLP | 234.387 | 0.690  ±0.065 | 0.685  ±0.108 | 0.780  ±0.045 | 0.802  ±0.032 | 0.793  ±0.041 | 0.779  ±0.046 | 0.916  ±0.012 | [[20.80 Â± 0.75, 0.00 Â± 0.00, 0.00 Â± 0.00, 0.20 Â± 0.40, 0.40 Â± 0.49],  [0.60 Â± 0.49, 10.60 Â± 1.36, 0.60 Â± 0.49, 1.00 Â± 0.63, 0.00 Â± 0.00],  [0.20 Â± 0.40, 5.00 Â± 5.59, 13.00 Â± 5.73, 11.60 Â± 3.32, 0.00 Â± 0.00],  [0.60 Â± 0.49, 2.60 Â± 3.38, 2.00 Â± 1.26, 47.20 Â± 2.79, 0.00 Â± 0.00],  [0.40 Â± 0.49, 0.20 Â± 0.40, 0.00 Â± 0.00, 0.60 Â± 0.49, 0.40 Â± 0.49]] |
| LOG-NICG-Z | 13155 | 6 | MLP | 46.659 | 0.246  ±0.098 | 0.417  ±0.108 | 0.470  ±0.082 | 0.269  ±0.062 | 0.415  ±0.073 | 0.297  ±0.074 | 0.817  ±0.027 | [[18.10 Â± 3.40, 0.40 Â± 0.55, 1.40 Â± 0.00, 0.00 Â± 0.00, 0.00 Â± 0.00],  [0.40 Â± 0.29, 9.40 Â± 3.01, 1.20 Â± 1.20, 1.80 Â± 2.23, 0.00 Â± 0.00],  [0.40 Â± 0.49, 8.20 Â± 8.45, 8.20 Â± 3.87, 12.20 Â± 4.45, 0.00 Â± 0.00],  [0.40 Â± 0.49, 7.80 Â± 10.46, 1.40 Â± 1.50, 41.40 Â± 4.37, 0.00 Â± 0.00],  [0.20 Â± 0.40, 0.40 Â± 0.49, 0.20 Â± 0.41, 0.80 Â± 0.75, 0.00 Â± 0.00]] |
| LOG-NPN-Z | 15672 | 97 | XGB | 75.489 | 0.391  ±0.155 | 0.573  ±0.222 | 0.552  ±0.204 | 0.641  ±0.256 | 0.552  ±0.204 | 0.541  ±0.199 | 0.821  ±0.337 | [[20.80 Â± 1.17, 0.20 Â± 0.40, 0.00 Â± 0.00, 0.20 Â± 0.40, 0.20 Â± 0.40],  [0.00 Â± 0.00, 11.80 Â± 2.32, 1.00 Â± 1.26, 1.00 Â± 1.26, 0.00 Â± 0.00],  [0.20 Â± 0.40, 12.00 Â± 9.88, 8.40 Â± 6.59, 8.40 Â± 5.89, 0.00 Â± 0.00],  [0.60 Â± 0.49, 11.20 Â± 9.20, 1.20 Â± 0.75, 39.20 Â± 8.93, 0.00 Â± 0.00],  [0.20 Â± 0.40, 0.80 Â± 0.75, 0.20 Â± 0.40, 0.40 Â± 0.80, 0.00 Â± 0.00]] |
| LOG-RQN | 12548 | 6 | XGB | 144.856 | 0.472  ±0.050 | 0.562  ±0.085 | 0.695  ±0.042 | 0.641  ±0.039 | 0.632  ±0.037 | 0.633  ±0.037 | 0.823  ±0.035 | [[21.00 Â± 0.63, 0.40 Â± 0.49, 0.00 Â± 0.00, 0.00 Â± 0.00, 0.00 Â± 0.00],  [0.40 Â± 0.49, 9.60 Â± 3.01, 1.40 Â± 1.20, 1.80 Â± 2.23, 0.00 Â± 0.00],  [0.40 Â± 0.49, 8.20 Â± 8.45, 8.20 Â± 5.42, 12.20 Â± 4.45, 0.00 Â± 0.00],  [0.40 Â± 0.49, 7.80 Â± 10.46, 1.40 Â± 1.50, 43.20 Â± 8.47, 0.00 Â± 0.00],  [0.20 Â± 0.40, 0.40 Â± 0.49, 0.20 Â± 0.40, 0.80 Â± 0.75, 0.00 Â± 0.00]] |
| LOG-RQN-Z | 11790 | 97 | XGB | 127.614 | 0.432  ±0.066 | 0.566  ±0.082 | 0.605  ±0.051 | 0.642  ±0.031 | 0.599  ±0.055 | 0.607  ±0.041 | 0.816  ±0.032 | [[21.00 Â± 0.63, 0.40 Â± 0.49, 0.00 Â± 0.00, 0.40 Â± 0.80, 0.00 Â± 0.00],  [0.40 Â± 0.49, 10.00 Â± 3.46, 1.20 Â± 1.17, 1.60 Â± 2.33, 0.00 Â± 0.00],  [0.20 Â± 0.40, 8.40 Â± 9.09, 8.20 Â± 5.46, 11.60 Â± 4.84, 0.00 Â± 0.00],  [0.60 Â± 0.49, 7.40 Â± 9.73, 1.20 Â± 1.17, 44.00 Â± 10.26, 0.00 Â± 0.00],  [0.20 Â± 0.40, 0.60 Â± 0.80, 0.20 Â± 0.40, 0.40 Â± 0.80, 0.00 Â± 0.00]] |
| LOG-NICG-Z | 11790 | 22 | XGB | 69.248 | 0.362  ±0.171 | 0.469  ±0.099 | 0.513  ±0.132 | 0.535  ±0.222 | 0.502  ±0.160 | 0.472  ±0.200 | 0.818  ±0.061 | [[18.00 Â± 0.33, 0.40 Â± 0.49, 0.00 Â± 0.00, 0.00 Â± 0.00, 0.00 Â± 0.00],  [0.40 Â± 0.49, 8.60 Â± 3.01, 1.40 Â± 1.20, 1.80 Â± 2.23, 0.00 Â± 0.00],  [0.40 Â± 0.49, 8.20 Â± 8.45, 9.20 Â± 3.42, 12.20 Â± 4.45, 0.00 Â± 0.00],  [0.40 Â± 0.49, 7.80 Â± 10.46, 1.40 Â± 1.50, 43.20 Â± 8.47, 0.00 Â± 0.00],  [0.20 Â± 0.40, 0.40 Â± 0.49, 0.20 Â± 0.40, 0.80 Â± 0.75, 0.00 Â± 0.00]] |
