# Supplementary material for: Normalization and Selecting Non-Differentially Expressed Genes Improve Machine Learning Modelling of Cross-Platform Transcriptomic Data
Source: Trans Artif Intell. Author manuscript; Available in PMC 2025 Jul 8. (PMC12235674; doi:10.53941/tai.2025.100005)
Supplement: Supplementary [file NIHMS2087281-supplement-Supplementary.zip › Supplementary table 10.docx]

| Supplementary table 10. Average performance results (mean ± standard deviation) of the best-performing models (with the highest Kappa) on data constructed using DEG and NDEG genes selected via one-way ANOVA. (**Model-A**) | | | | | | | | | | | | |
| --- | --- | --- | --- | --- | --- | --- | --- | --- | --- | --- | --- | --- |
| Normalization _Method | DEG_ number | NDEG_ number | Model | E*_value_* | Kappa | Balanced _Accuracy | Accuracy | Precision | Recall | F1 | AUC | Confusion Matrix |
| LOG-NPN-Z | 13155 | 22 | SVM | 69.623 | 0.308  ±0.093 | 0.478  ±0.095 | 0.495  ±0.072 | 0.333  ±0.042 | 0.461  ±0.068 | 0.344  ±0.064 | 0.802  ±0.085 | [[19.60 Â± 0.63, 0.40 Â± 0.49, 1.00 Â± 0.00, 0.00 Â± 0.00, 0.00 Â± 0.00],  [0.40 Â± 0.49, 9.60 Â± 3.01, 1.40 Â± 1.20, 1.80 Â± 2.23, 0.00 Â± 0.00],  [0.40 Â± 0.49, 8.20 Â± 8.45, 8.20 Â± 5.42, 12.20 Â± 4.45, 0.00 Â± 0.00],  [0.40 Â± 0.49, 7.80 Â± 10.46, 1.40 Â± 1.50, 43.20 Â± 8.47, 0.00 Â± 0.00],  [0.20 Â± 0.40, 0.40 Â± 0.49, 0.20 Â± 0.40, 0.80 Â± 0.75, 0.00 Â± 0.00]] |
| LOG-RQN | 12621 | 6 | SVM | 147.756 | 0.581  ±0.128 | 0.615  ±0.125 | 0.718  ±112 | 0.748  ±0.053 | 0.703  ±0.102 | 0.697  ±0.095 | 0.901  ±0.041 | [[20.60 Â± 1.02, 0.00 Â± 0.00, 0.00 Â± 0.00, 0.60 Â± 0.80, 0.00 Â± 0.00],  [0.60 Â± 0.49, 9.20 Â± 0.75, 1.20 Â± 0.75, 2.40 Â± 1.02, 0.00 Â± 0.00],  [0.20 Â± 0.40, 1.00 Â± 1.26, 13.40 Â± 3.01, 14.80 Â± 3.31, 0.00 Â± 0.00],  [0.60 Â± 0.49, 0.20 Â± 0.40, 1.40 Â± 0.80, 49.80 Â± 2.86, 0.00 Â± 0.00],  [0.60 Â± 0.49, 0.00 Â± 0.00, 0.40 Â± 0.49, 1.00 Â± 0.63, 0.00 Â± 0.00]] |
| LOG-RQN-Z | 12621 | 6 | SVM | 166.895 | 0.594  ±0.134 | 0.609  ±0.074 | 0.719  ±0.111 | 0.750  ±0.050 | 0.714  ±0.107 | 0.704  ±0.100 | 0.901  ±0.046 | [[19.80 Â± 1.94, 1.00 Â± 0.00, 0.00 Â± 0.00, 0.00 Â± 0.00, 0.60 Â± 0.49],  [0.80 Â± 0.40, 11.40 Â± 1.36, 0.00 Â± 0.00, 1.00 Â± 0.63, 0.00 Â± 0.00],  [1.00 Â± 0.89, 7.20 Â± 1.07, 8.40 Â± 3.56, 11.80 Â± 2.79, 0.40 Â± 0.49],  [1.80 Â± 0.40, 12.20 Â± 0.98, 3.20 Â± 1.17, 35.60 Â± 1.41, 0.00 Â± 0.00],  [0.00 Â± 0.00, 0.00 Â± 0.00, 1.00 Â± 0.00, 0.20 Â± 0.40, 0.60 Â± 0.49]] |
| LOG-NICG-Z | 13155 | 22 | SVM | 66.822 | 0.337  ±0.135 | 0.479  ±0.118 | 0.515  ±0.115 | 0.539  ±0.285 | 0.488  ±0.104 | 0.388  ±0.143 | 0.812  ±0.119 | [[19.90 Â± 0.63, 0.40 Â± 0.49, 0.80 Â± 0.00, 0.00 Â± 0.00, 0.00 Â± 0.00],  [0.40 Â± 0.49, 9.60 Â± 3.01, 1.40 Â± 1.20, 1.80 Â± 2.23, 0.00 Â± 0.00],  [0.40 Â± 0.49, 8.20 Â± 8.45, 8.20 Â± 5.42, 12.20 Â± 4.45, 0.00 Â± 0.00],  [0.40 Â± 0.49, 7.80 Â± 10.46, 1.40 Â± 1.50, 43.20 Â± 8.47, 0.00 Â± 0.00],  [0.20 Â± 0.40, 0.40 Â± 0.49, 0.20 Â± 0.40, 0.80 Â± 0.75, 0.00 Â± 0.00]] |
| LOG-NPN-Z | 12427 | 6 | RF | 101.295 | 0.394  ±0.058 | 0.440  ±0.050 | 0.554  ±0.052 | 0.645  ±0.057 | 0.590  ±0.049 | 0.563  ±0.066 | 0.821  ±0.028 | [[18.50 Â± 0.43, 0.40 Â± 0.49, 1.40 Â± 0.00, 0.00 Â± 0.00, 0.00 Â± 0.00],  [0.40 Â± 0.29, 9.40 Â± 3.01, 1.20 Â± 1.20, 1.80 Â± 2.23, 0.00 Â± 0.00],  [0.40 Â± 0.49, 8.20 Â± 8.45, 8.20 Â± 5.12, 12.20 Â± 4.45, 0.00 Â± 0.00],  [0.40 Â± 0.49, 7.80 Â± 10.46, 1.40 Â± 1.50, 41.20 Â± 8.17, 0.00 Â± 0.00],  [0.20 Â± 0.40, 0.40 Â± 0.49, 0.20 Â± 0.41, 0.80 Â± 0.75, 0.00 Â± 0.00]] |
| LOG-RQN | 11408 | 6 | RF | 109.358 | 0.425  ±0.066 | 0.437  ±0.042 | 0.623  ±0.040 | 0.600  ±0.049 | 0.616  ±0.043 | 0.585  ±0.050 | 0.793  ±0.028 | [[19.00 Â± 2.19, 1.00 Â± 1.67, 0.20 Â± 0.45, 1.60 Â± 1.52, 0.00 Â± 0.00],  [0.80 Â± 1.17, 5.20 Â± 1.64, 5.20 Â± 0.84, 2.20 Â± 1.10, 0.00 Â± 0.00],  [0.60 Â± 1.34, 2.60 Â± 2.70, 18.20 Â± 3.43, 8.60 Â± 5.77, 0.20 Â± 0.45],  [10.00 Â± 8.48, 13.00 Â± 8.72, 17.20 Â± 8.47, 15.40 Â± 8.80, 1.40 Â±1.14],  [1.00 Â± 0.63, 0.20 Â± 0.45, 0.20 Â± 0.45, 0.40 Â± 0.55, 0.00 Â± 0.00]] |
| LOG-RQN-Z | 13744 | 6 | RF | 133.644 | 0.432  ±0.047 | 0.447  ±0.021 | 0.632  ±0.052 | 0.613  ±0.045 | 0.620  ±0.041 | 0.603  ±0.045 | 0.790  ±0.038 | [[19.60 Â± 0.55, 0.40 Â± 0.49, 1.40 Â± 0.00, 0.00 Â± 0.00, 0.00 Â± 0.00],  [0.40 Â± 0.49, 9.40 Â± 3.05, 1.20 Â± 1.20, 1.80 Â± 2.23, 0.00 Â± 0.00],  [0.40 Â± 0.49, 8.20 Â± 8.45, 8.20 Â± 3.41, 12.20 Â± 4.45, 0.00 Â± 0.00],  [0.40 Â± 0.49, 7.80 Â± 7.46, 1.20 Â± 1.50, 41.20 Â± 8.43, 0.00 Â± 0.00],  [0.20 Â± 0.40, 0.40 Â± 0.49, 0.20 Â± 0.40, 0.80 Â± 0.75, 0.00 Â± 0.00]] |
| LOG-NICG-Z | 15672 | 22 | RF | 59.882 | 0.297  ±0.070 | 0.376  ±0.067 | 0.562  ±0.025 | 0.572  ±0.108 | 0.536  ±0.027 | 0.494  ±0.022 | 0.755  ±0.065 | [[18.20 Â± 2.71, 0.80 Â± 0.98, 2.20 Â± 1.04, 0.20 Â± 0.40, 0.00 Â± 0.00],  [2.60 Â± 2.87, 1.60 Â± 1.20, 7.80 Â± 3.97, 1.60 Â± 3.20, 0.00 Â± 0.00],  [1.60 Â± 2.33, 0.40 Â± 0.80, 23.80 Â± 1.89, 3.00 Â± 0.98, 0.40 Â± 0.80],  [11.60 Â± 10.42, 0.80 Â± 0.75, 34.20 Â± 5.98, 5.00 Â± 1.67, 0.20 Â± 0.40],  [1.20 Â± 0.75, 0.00 Â± 0.00, 0.80 Â± 0.75, 0.00 Â± 0.00, 0.00 Â± 0.00]] |
| LOG-NPN-Z | 12303 | 97 | LR | 46.588 | 0.208  ±0.052 | 0.367  ±0.043 | 0.386  ±0.033 | 0.243  ±0.007 | 0.392  ±0.039 | 0.266  ±0.031 | 0.870  ±0.032 | [[17.60 Â± 2.24, 0.00 Â± 0.00, 3.60 Â± 3.14, 0.00 Â± 0.00, 0.00 Â± 0.00],  [0.20 Â± 0.40, 0.00 Â± 0.00, 13.60 Â± 1.02, 0.00 Â± 0.00, 0.00 Â± 0.00],  [0.00 Â± 0.00, 0.00 Â± 0.00, 28.00 Â± 1.41, 0.00 Â± 0.00, 0.00 Â± 0.00],  [0.60 Â± 0.49, 0.00 Â± 0.00, 52.40 Â± 2.06, 0.00 Â± 0.00, 0.00 Â± 0.00],  [0.20 Â± 0.40, 0.00 Â± 0.00, 1.80 Â± 0.40, 0.00 Â± 0.00, 0.00 Â± 0.00]] |
| LOG-RQN | 12621 | 6 | LR | 260.144 | 0.721  ±0.081 | 0.621  ±0.037 | 0.802  ±0.050 | 0.813  ±0.053 | 0.812  ±0.048 | 0.796  ±0.058 | 0.936  ±0.022 | [[21.20 Â± 1.17, 0.00 Â± 0.00, 0.00 Â± 0.00, 0.00 Â± 0.00, 0.00 Â± 0.00],  [0.80 Â± 0.40, 10.00 Â± 0.89, 0.80 Â± 0.40, 2.20 Â± 0.40, 0.00 Â± 0.00],  [0.00 Â± 0.00, 2.40 Â± 1.36, 12.00 Â± 0.63, 14.40 Â± 1.36, 0.00 Â± 0.00],  [0.00 Â± 0.00, 0.00 Â± 0.00, 1.80 Â± 1.17, 50.40 Â± 1.74, 0.00 Â± 0.00],  [2.00 Â± 0.00, 0.00 Â± 0.00, 0.00 Â± 0.00, 0.00 Â± 0.00, 0.00 Â± 0.00]] |
| LOG-RQN-Z | 15672 | 6 | LR | 258.902 | 0.716  ±0.047 | 0.646  ±0.044 | 0.801  ±0.015 | 0.817  ±0.004 | 0.808  ±0.012 | 0.792  ±0.015 | 0.918  ±0.013 | [[21.20 Â± 1.17, 0.00 Â± 0.00, 0.00 Â± 0.00, 0.00 Â± 0.00, 0.00 Â± 0.40],  [0.40 Â± 0.49, 10.00 Â± 2.10, 0.80 Â± 0.75, 2.20 Â± 1.47, 0.20 Â± 0.40],  [0.00 Â± 0.00, 3.60 Â± 1.02, 14.20 Â± 1.47, 11.00 Â± 1.79, 0.00 Â± 0.00],  [0.00 Â± 0.00, 1.00 Â± 0.00, 3.60 Â± 0.49, 47.60 Â± 1.50, 0.20 Â± 0.40],  [1.60 Â± 0.49, 0.00 Â± 0.00, 0.00 Â± 0.00, 0.00 Â± 0.00, 0.20 Â± 0.40]] |
| LOG-NICG-Z | 12189 | 22 | LR | 47.318 | 0.216  ±0.057 | 0.363  ±0.042 | 0.426  ±0.042 | 0.251  ±0.007 | 0.406  ±0.048 | 0.278  ±0.037 | 0.889  ±0.009 | [[17.60 Â± 3.44, 0.00 Â± 0.00, 4.00 Â± 3.16, 0.00 Â± 0.00, 0.00 Â± 0.00],  [0.20 Â± 0.40, 0.00 Â± 0.00, 13.60 Â± 0.49, 0.00 Â± 0.00, 0.00 Â± 0.00],  [0.00 Â± 0.00, 0.00 Â± 0.00, 29.20 Â± 1.17, 0.00 Â± 0.00, 0.00 Â± 0.00],  [0.60 Â± 0.49, 0.00 Â± 0.00, 50.80 Â± 1.60, 0.00 Â± 0.00, 0.00 Â± 0.00],  [0.20 Â± 0.40, 0.00 Â± 0.00, 1.80 Â± 0.40, 0.00 Â± 0.00, 0.00 Â± 0.00]] |
| LOG-NPN-Z | 11408 | 6 | MLP | 73.820 | 0.297  ±0.063 | 0.463  ±0.074 | 0.491  ±0.041 | 0.306  ±0.048 | 0.453  ±0.046 | 0.336  ±0.049 | 0.858  ±0.014 | [[19.20 Â± 0.63, 0.40 Â± 0.43, 1.00 Â± 0.00, 0.00 Â± 0.00, 0.00 Â± 0.00],  [0.40 Â± 0.49, 9.60 Â± 1.99, 1.40 Â± 1.20, 1.80 Â± 2.23, 0.00 Â± 0.00],  [0.40 Â± 0.49, 8.20 Â± 6.41, 8.40 Â± 4.12, 12.20 Â± 4.45, 0.00 Â± 0.00],  [0.40 Â± 0.49, 7.80 Â± 10.46, 1.40 Â± 1.50, 43.20 Â±5.41, 0.00 Â± 0.00],  [0.20 Â± 0.40, 0.40 Â± 0.49, 0.20 Â± 0.40, 0.80 Â± 0.75, 0.00 Â± 0.00]] |
| LOG-RQN | 12303 | 58 | MLP | 311.003 | 0.734  ±0.048 | 0.707  ±0.052 | 0.812  ±0.041 | 0.825  ±0.046 | 0.818  ±0.040 | 0.808  ±0.037 | 0.931  ±0.027 | [[20.80 Â± 1.17, 0.60 Â± 0.49, 0.00 Â± 0.00, 0.20 Â± 0.40, 0.40 Â± 0.49],  [0.80 Â± 0.40, 12.00 Â± 0.63, 0.00 Â± 0.00, 1.00 Â± 0.63, 0.00 Â± 0.00],  [0.60 Â± 0.80, 4.80 Â± 1.26, 10.00 Â± 0.98, 11.80 Â± 1.47, 0.60 Â± 0.49],  [1.80 Â± 0.40, 6.80 Â± 1.47, 3.80 Â± 0.75, 40.00 Â± 0.75, 0.20 Â± 0.40],  [0.00 Â± 0.00, 0.00 Â± 0.00, 0.80 Â± 0.40, 0.00 Â± 0.00, 1.00 Â± 0.00]] |
| LOG-RQN-Z | 13409 | 97 | MLP | 252.866 | 0.725  ±0.103 | 0.649  ±0.045 | 0.816  ±0.068 | 0.823  ±0.060 | 0.811  ±0.059 | 0.801  ±0.075 | 0.923  ±0.021 | [[20.80 Â± 0.75, 0.00 Â± 0.00, 0.00 Â± 0.00, 0.20 Â± 0.40, 0.40 Â± 0.49],  [0.60 Â± 0.49, 10.60 Â± 1.36, 0.60 Â± 0.49, 1.00 Â± 0.63, 0.00 Â± 0.00],  [0.20 Â± 0.40, 5.00 Â± 5.59, 13.00 Â± 5.73, 11.60 Â± 3.32, 0.00 Â± 0.00],  [0.60 Â± 0.49, 2.60 Â± 3.38, 2.00 Â± 1.26, 47.20 Â± 2.79, 0.00 Â± 0.00],  [0.40 Â± 0.49, 0.20 Â± 0.40, 0.00 Â± 0.00, 0.60 Â± 0.49, 0.40 Â± 0.49]] |
| LOG-NICG-Z | 13155 | 6 | MLP | 46.659 | 0.246  ±0.098 | 0.417  ±0.108 | 0.470  ±0.082 | 0.269  ±0.062 | 0.415  ±0.073 | 0.297  ±0.074 | 0.817  ±0.027 | [[18.10 Â± 3.40, 0.40 Â± 0.55, 1.40 Â± 0.00, 0.00 Â± 0.00, 0.00 Â± 0.00],  [0.40 Â± 0.29, 9.40 Â± 3.01, 1.20 Â± 1.20, 1.80 Â± 2.23, 0.00 Â± 0.00],  [0.40 Â± 0.49, 8.20 Â± 8.45, 8.20 Â± 3.87, 12.20 Â± 4.45, 0.00 Â± 0.00],  [0.40 Â± 0.49, 7.80 Â± 10.46, 1.40 Â± 1.50, 41.40 Â± 4.37, 0.00 Â± 0.00],  [0.20 Â± 0.40, 0.40 Â± 0.49, 0.20 Â± 0.41, 0.80 Â± 0.75, 0.00 Â± 0.00]] |
| LOG-NPN-Z | 11408 | 6 | XGB | 108.177 | 0.492  ±0.185 | 0.514  ±0.075 | 0.612  ±0.170 | 0.682  ±0.158 | 0.615  ±0.175 | 0.601  ±0.187 | 0.826  ±0.044 | [[22.00 Â± 0.63, 0.00 Â± 0.00, 0.00 Â± 0.00, 0.00 Â± 0.00, 0.00 Â± 0.00],  [0.00 Â± 0.00, 11.80 Â± 1.10, 0.20 Â± 0.40, 1.40 Â± 0.80, 0.00 Â± 0.00],  [0.00 Â± 0.00, 3.40 Â± 0.49, 12.20 Â± 1.10, 12.80 Â± 1.17, 0.00 Â± 0.00],  [0.00 Â± 0.00, 0.80 Â± 0.40, 2.80 Â± 0.40, 49.00 Â± 1.10, 0.00 Â± 0.00],  [1.40 Â± 0.49, 0.00 Â± 0.00, 0.00 Â± 0.00, 0.20 Â± 0.40, 0.00 Â± 0.00]] |
| LOG-RQN | 13744 | 6 | XGB | 174.674 | 0.515  ±0.042 | 0.508  ±0.030 | 0.660  ±0.036 | 0.658  ±0.018 | 0.661  ±0.035 | 0.652  ±0.023 | 0.831  ±0.030 | [[21.00 Â± 0.89, 0.20 Â± 0.40, 0.00 Â± 0.00, 0.00 Â± 0.00, 0.00 Â± 0.00],  [0.40 Â± 0.49, 4.00 Â± 1.10, 3.60 Â± 1.62, 5.80 Â± 0.98, 0.00 Â± 0.00],  [0.40 Â± 0.49, 1.00 Â± 1.10, 17.60 Â± 2.65, 9.60 Â± 2.80, 0.00 Â± 0.00],  [5.00 Â± 1.79, 1.20 Â± 0.98, 12.20 Â± 3.71, 33.80 Â± 4.83, 0.40 Â± 0.49],  [0.80 Â± 0.40, 0.00 Â± 0.00, 1.00 Â± 0.00, 0.00 Â± 0.00, 0.00 Â± 0.00]] |
| LOG-RQN-Z | 12621 | 6 | XGB | 163.658 | 0.525  ±0.059 | 0.511  ±0.038 | 0.677  ±0.042 | 0.678  ±0.035 | 0.679  ±0.045 | 0.670  ±0.034 | 0.818  ±0.050 | [[22.60 Â± 0.49, 0.00 Â± 0.00, 0.00 Â± 0.00, 0.20 Â± 0.40, 0.00 Â± 0.00],  [1.20 Â± 0.98, 4.8 Â± 2.79, 4.40 Â± 2.50, 2.40 Â± 0.80, 0.20 Â± 0.40],  [0.60 Â± 0.80, 3.20 Â± 4.53, 17.00 Â± 5.46, 8.00 Â± 2.19, 0.00 Â± 0.00],  [4.60 Â± 1.85, 5.80 Â± 4.83, 13.20 Â± 7.19, 27.00 Â± 6.90, 1.20 Â± 1.17],  [1.00 Â± 0.89, 0.20 Â± 0.40, 0.00 Â± 0.00, 0.20 Â± 0.40, 0.20 Â± 0.40]] |
| LOG-NICG-Z | 12621 | 6 | XGB | 66.812 | 0.355  ±0.153 | 0.447  ±0.097 | 0.505  ±0.200 | 0.588  ±0.073 | 0.508  ±0.206 | 0.505  ±0.197 | 0.766  ±0.043 | [[21.20 Â± 1.17, 0.20 Â± 0.45, 0.00 Â± 0.00, 1.40 Â± 1.52, 0.00 Â± 0.00],  [0.60 Â± 0.89, 3.80 Â± 0.84, 4.60 Â± 1.14, 4.80 Â± 1.30, 0.20 Â± 0.45],  [1.20 Â± 1.64, 0.20 Â± 0.45, 19.60 Â± 1.67, 8.00 Â± 2.24, 0.00 Â± 0.00],  [4.20 Â± 2.17, 1.40 Â± 1.14, 14.40 Â± 2.30, 31.60 Â± 2.70, 0.80 Â± 0.84],  [1.20 Â± 0.84, 0.00 Â± 0.00, 0.00 Â± 0.00, 0.80 Â± 0.84, 0.20 Â± 0.45]] |
