# Supplementary material for: Normalization and Selecting Non-Differentially Expressed Genes Improve Machine Learning Modelling of Cross-Platform Transcriptomic Data
Source: Trans Artif Intell. Author manuscript; Available in PMC 2025 Jul 8. (PMC12235674; doi:10.53941/tai.2025.100005)
Supplement: Supplementary [file NIHMS2087281-supplement-Supplementary.zip › Supplementary table 11.docx]

| Supplementary table 11. Model performance metrics of the top-performing models (with the highest Balanced Accuracy) on data constructed using DEG and NDEG features selected via logistic regression (LR) with elastic net regularization. (**Model-S**) | | | | | | | | | | | |
| --- | --- | --- | --- | --- | --- | --- | --- | --- | --- | --- | --- |
| Normalization _Method | DEG_ number | NDEG_ number | Model | Kappa | Balanced _Accuracy | Accuracy | Precision | Recall | F1 | AUC | Confusion Matrix |
| LOG-NPN-Z | 46 | 55 | SVM | 0.334 | 0.564 | 0.563 | 0.528 | 0.563 | 0.502 | 0.760 | [[22 0 0 0 1]  [ 0 0 1 11 1]  [ 0 1 2 26 0]  [ 0 1 2 42 8]  [ 0 0 0 0 1]] |
| LOG-RQN | 3262 | 106 | SVM | 0.489 | 0.692 | 0.622 | 0.686 | 0.622 | 0.634 | 0.861 | [[22 0 0 0 1]  [ 3 8 1 1 0]  [ 0 17 6 4 0]  [ 0 8 10 36 0]  [ 0 0 0 0 2]] |
| LOG-RQN-Z | 4131 | 55 | SVM | 0.470 | 0.707 | 0.622 | 0.647 | 0.622 | 0.629 | 0.777 | [[18 3 0 1 0]  [ 1 7 1 3 0]  [ 0 4 18 10 0]  [ 0 0 21 29 1]  [ 0 0 0 0 2]] |
| LOG-NICG-Z | 1992 | 55 | SVM | 0.401 | 0.627 | 0.597 | 0.502 | 0.597 | 0.530 | 0.715 | [[18 4 0 2 0]  [ 0 7 0 6 0]  [ 0 4 0 24 0]  [ 0 3 0 44 5]  [ 0 0 0 0 2]] |
| LOG-NPN-Z | 152 | 106 | RF | 0.572 | 0.561 | 0.706 | 0.701 | 0.706 | 0.699 | 0.834 | [[22 0 0 1 0]  [ 0 7 0 7 0]  [ 0 0 18 11 0]  [ 1 2 11 37 0]  [ 0 0 1 1 0]] |
| LOG-RQN | 2513 | 7 | RF | 0.652 | 0.554 | 0.773 | 0.763 | 0.773 | 0.745 | 0.908 | [[20 0 0 1 0]  [ 1 4 4 5 0]  [ 0 1 16 11 0]  [ 1 0 1 52 0]  [ 0 0 1 1 0]] |
| LOG-RQN-Z | 861 | 55 | RF | 0.622 | 0.530 | 0.748 | 0.715 | 0.748 | 0.716 | 0.879 | [[21 0 0 0 0]  [ 1 2 7 4 0]  [ 0 2 18 10 0]  [ 2 0 3 48 0]  [ 0 0 1 0 0]] |
| LOG-NICG-Z | 1945 | 55 | RF | 0.496 | 0.553 | 0.639 | 0.637 | 0.639 | 0.628 | 0.793 | [[22 0 0 1 0]  [ 2 10 1 0 0]  [ 1 3 12 11 0]  [ 4 9 9 32 0]  [ 0 0 1 1 0]] |
| LOG-NPN-Z | 46 | 55 | LR | 0.413 | 0.605 | 0.630 | 0.634 | 0.630 | 0.553 | 0.788 | [[22 0 0 1 0]  [ 0 1 0 12 0]  [ 0 3 2 24 0]  [ 0 3 1 49 0]  [ 0 0 0 0 1]] |
| LOG-RQN | 3631 | 55 | LR | 0.573 | 0.676 | 0.697 | 0.711 | 0.697 | 0.700 | 0.897 | [[20 0 0 1 0]  [ 1 8 3 0 0]  [ 1 3 17 8 0]  [ 2 5 10 37 1]  [ 0 0 1 0 1]] |
| LOG-RQN-Z | 3051 | 1 | LR | 0.442 | 0.666 | 0.613 | 0.624 | 0.613 | 0.609 | 0.738 | [[18 0 1 4 1]  [ 0 7 3 4 0]  [ 1 3 10 16 1]  [ 1 0 10 37 1]  [ 0 0 0 0 1]] |
| LOG-NICG-Z | 326 | 106 | LR | 0.533 | 0.542 | 0.706 | 0.553 | 0.706 | 0.609 | 0.885 | [[24 0 0 0 0]  [ 0 8 0 3 0]  [ 0 0 0 29 0]  [ 0 1 0 52 0]  [ 0 0 0 2 0]] |
| LOG-NPN-Z | 1662 | 7 | MLP | 0.514 | 0.532 | 0.681 | 0.777 | 0.681 | 0.590 | 0.889 | [[22 0 0 1 0]  [ 0 9 0 4 0]  [ 0 2 1 28 0]  [ 0 1 0 49 0]  [ 0 1 0 1 0]] |
| LOG-RQN | 3631 | 55 | MLP | 0.734 | 0.618 | 0.815 | 0.796 | 0.815 | 0.801 | 0.939 | [[23 0 1 0 0]  [ 2 7 5 0 0]  [ 1 1 20 7 0]  [ 1 1 1 47 0]  [ 0 0 1 1 0]] |
| LOG-RQN-Z | 827 | 55 | MLP | 0.674 | 0.622 | 0.773 | 0.792 | 0.773 | 0.761 | 0.924 | [[22 1 0 0 0]  [ 0 10 0 2 0]  [ 0 10 12 7 0]  [ 1 2 2 48 0]  [ 0 0 1 1 0]] |
| LOG-NICG-Z | 282 | 12 | MLP | 0.562 | 0.574 | 0.706 | 0.705 | 0.706 | 0.644 | 0.882 | [[22 0 0 1 0]  [ 0 11 0 2 0]  [ 0 4 4 23 0]  [ 0 1 2 47 0]  [ 0 0 0 2 0]] |
| LOG-NPN-Z | 2775 | 7 | XGB | 0.326 | 0.634 | 0.487 | 0.489 | 0.487 | 0.469 | 0.753 | [[18 2 0 1 0]  [ 0 11 0 2 1]  [ 0 13 0 14 3]  [ 0 11 3 28 11]  [ 0 0 0 0 1]] |
| LOG-RQN | 4255 | 55 | XGB | 0.475 | 0.582 | 0.639 | 0.643 | 0.639 | 0.634 | 0.764 | [[22 2 0 0 0]  [ 1 5 3 5 0]  [ 0 1 12 15 0]  [ 1 0 14 36 0]  [ 0 0 0 1 1]] |
| LOG-RQN-Z | 834 | 7 | XGB | 0.443 | 0.581 | 0.622 | 0.633 | 0.622 | 0.613 | 0.759 | [[22 0 0 1 0]  [ 1 6 1 6 0]  [ 0 0 9 21 0]  [ 0 1 13 36 0]  [ 0 0 1 0 1]] |
| LOG-NICG-Z | 336 | 12 | XGB | 0.429 | 0.475 | 0.622 | 0.620 | 0.622 | 0.555 | 0.786 | [[19 1 0 4 0]  [ 0 10 0 5 0]  [ 0 2 1 25 0]  [ 0 5 1 44 0]  [ 0 0 0 2 0]] |
