# Supplementary material for: Normalization and Selecting Non-Differentially Expressed Genes Improve Machine Learning Modelling of Cross-Platform Transcriptomic Data
Source: Trans Artif Intell. Author manuscript; Available in PMC 2025 Jul 8. (PMC12235674; doi:10.53941/tai.2025.100005)
Supplement: Supplementary [file NIHMS2087281-supplement-Supplementary.zip › Supplementary table 12.docx]

| Supplementary table 12. Comparison of the numbers of DEGs and NDEGs obtained using one-way ANOVA and FDR-adjusted one-way ANOVA, respectively. (**Model-S**) | | | | |
| --- | --- | --- | --- | --- |
|  | Threshold  (P_value) | Selected feature number | Threshold  (FDR-corrected P_value) | Selected feature number |
| DEG | 0.001 | 10124 | 0.001 | 9864 |
|  | 0.002 | 10523 | 0.002 | 10291 |
|  | 0.003 | 10756 | 0.003 | 10545 |
|  | 0.004 | 10936 | 0.004 | 10706 |
|  | 0.005 | 11079 | 0.005 | 10856 |
|  | 0.006 | 11183 | 0.006 | 10976 |
|  | 0.007 | 11282 | 0.007 | 11084 |
|  | 0.008 | 11363 | 0.008 | 11169 |
|  | 0.009 | 11447 | 0.009 | 11243 |
|  | 0.01 | 11516 | 0.01 | 11311 |
|  | 0.02 | 12001 | 0.02 | 11820 |
|  | 0.03 | 12294 | 0.03 | 12117 |
|  | 0.05 | 12690 | 0.05 | 12341 |
|  | 0.07 | 12934 | 0.07 | 12546 |
|  | 0.1 | 13236 | 0.1 | 12796 |
| NDEG | 0.85 | 253 | 0.85 | 106 |
|  | 0.9 | 133 | 0.9 | 55 |
|  | 0.92 | 94 | 0.92 | 12 |
|  | 0.95 | 49 | 0.95 | 7 |
|  | 0.98 | 11 | 0.98 | 1 |
