# Supplementary material for: Normalization and Selecting Non-Differentially Expressed Genes Improve Machine Learning Modelling of Cross-Platform Transcriptomic Data
Source: Trans Artif Intell. Author manuscript; Available in PMC 2025 Jul 8. (PMC12235674; doi:10.53941/tai.2025.100005)
Supplement: Supplementary [file NIHMS2087281-supplement-Supplementary.zip › Supplementary table 13.docx]

| Supplementary table 13. Model performance metrics of the top-performing models (with the highest Balanced Accuracy) on data constructed using DEG and NDEG genes selected via FDR-corrected one-way ANOVA. (**Model-S**) | | | | | | | | | | | |
| --- | --- | --- | --- | --- | --- | --- | --- | --- | --- | --- | --- |
| Normalization _Method | DEG_ number | NDEG_ number | Model | Kappa | Balanced _Accuracy | Accuracy | Precision | Recall | F1 | AUC | Confusion Matrix |
| LOG-NPN-Z | 9864 | 55 | SVM | 0.371 | 0.599 | 0.605 | 0.626 | 0.605 | 0.521 | 0.831 | [[20 0 0 1 0]  [ 0 1 0 13 0]  [ 0 1 2 27 0]  [ 0 1 1 48 3]  [ 0 0 0 0 1]] |
| LOG-RQN | 10291 | 106 | SVM | 0.395 | 0.676 | 0.563 | 0.640 | 0.563 | 0.580 | 0.765 | [[18 0 0 1 0]  [ 0 7 5 3 0]  [ 0 0 14 14 1]  [ 1 0 18 26 9]  [ 0 0 0 0 2]] |
| LOG-RQN-Z | 12341 | 55 | SVM | 0.391 | 0.679 | 0.555 | 0.584 | 0.555 | 0.561 | 0.782 | [[18 2 0 0 0]  [ 1 9 2 3 0]  [ 0 4 13 13 0]  [ 1 0 21 24 6]  [ 0 0 0 0 2]] |
| LOG-NICG-Z | 10976 | 12 | SVM | 0.269 | 0.535 | 0.403 | 0.472 | 0.403 | 0.430 | 0.776 | [[21 0 0 1 1]  [ 0 5 0 1 9]  [ 0 5 0 16 10]  [ 0 1 0 21 27]  [ 0 0 0 0 1]] |
| LOG-NPN-Z | 10976 | 12 | RF | 0.519 | 0.503 | 0.681 | 0.662 | 0.681 | 0.654 | 0.811 | [[22 1 1 0 0]  [ 0 6 2 6 0]  [ 0 2 8 18 0]  [ 0 0 6 45 0]  [ 0 0 0 2 0]] |
| LOG-RQN | 10856 | 7 | RF | 0.721 | 0.562 | 0.815 | 0.776 | 0.815 | 0.783 | 0.937 | [[21 0 0 2 0]  [ 2 2 7 2 0]  [ 0 2 20 5 0]  [ 0 0 0 54 0]  [ 0 0 1 1 0]] |
| LOG-RQN-Z | 12796 | 7 | RF | 0.631 | 0.546 | 0.748 | 0.749 | 0.748 | 0.725 | 0.864 | [[19 0 1 1 0]  [ 1 3 9 2 0]  [ 0 1 22 6 0]  [ 1 0 6 45 0]  [ 0 0 1 1 0]] |
| LOG-NICG-Z | 11820 | 12 | RF | 0.388 | 0.449 | 0.597 | 0.626 | 0.597 | 0.559 | 0.753 | [[17 2 0 0 0]  [ 0 6 2 7 0]  [ 0 4 4 21 0]  [ 0 10 0 44 0]  [ 0 0 0 2 0]] |
| LOG-NPN-Z | 9864 | 55 | LR | 0.378 | 0.608 | 0.613 | 0.469 | 0.613 | 0.513 | 0.847 | [[20 0 0 1 0]  [ 0 2 0 12 0]  [ 0 2 0 28 0]  [ 0 2 1 50 0]  [ 0 0 0 0 1]] |
| LOG-RQN | 10856 | 55 | LR | 0.546 | 0.633 | 0.672 | 0.663 | 0.672 | 0.660 | 0.891 | [[23 0 1 0 0]  [ 3 8 2 1 0]  [ 5 4 12 10 0]  [ 1 4 6 36 1]  [ 0 0 1 0 1]] |
| LOG-RQN-Z | 11243 | 1 | LR | 0.493 | 0.674 | 0.639 | 0.679 | 0.639 | 0.655 | 0.764 | [[20 0 0 1 0]  [ 0 9 2 2 1]  [ 0 2 15 12 0]  [ 0 9 10 32 4]  [ 0 0 0 0 0]] |
| LOG-NICG-Z | 11820 | 12 | LR | 0.542 | 0.556 | 0.697 | 0.791 | 0.697 | 0.615 | 0.845 | [[22 1 0 0 0]  [ 0 11 0 2 0]  [ 0 3 1 26 0]  [ 0 2 0 49 1]  [ 0 0 0 1 0]] |
| LOG-NPN-Z | 12546 | 1 | MLP | 0.537 | 0.541 | 0.681 | 0.736 | 0.681 | 0.665 | 0.846 | [[21 2 0 0 0]  [ 0 9 0 3 0]  [ 0 8 6 13 0]  [ 0 8 2 45 0]  [ 0 2 0 0 0]] |
| LOG-RQN | 12546 | 106 | MLP | 0.724 | 0.614 | 0.815 | 0.800 | 0.815 | 0.802 | 0.928 | [[23 0 0 0 0]  [ 2 5 3 1 0]  [ 0 0 21 8 0]  [ 0 1 5 48 0]  [ 0 0 0 2 0]] |
| LOG-RQN-Z | 10856 | 12 | MLP | 0.698 | 0.599 | 0.790 | 0.767 | 0.790 | 0.774 | 0.939 | [[22 0 0 1 0]  [ 1 7 6 1 0]  [ 0 4 19 7 0]  [ 1 0 2 46 0]  [ 0 0 0 2 0]] |
| LOG-NICG-Z | 10706 | 55 | MLP | 0.560 | 0.540 | 0.706 | 0.560 | 0.706 | 0.619 | 0.890 | [[24 0 0 1 0]  [ 0 12 0 3 0]  [ 0 6 0 20 0]  [ 0 3 0 48 0]  [ 0 0 0 2 0]] |
| LOG-NPN-Z | 12341 | 55 | XGB | 0.299 | 0.569 | 0.555 | 0.618 | 0.555 | 0.505 | 0.742 | [[ 7 1 0 13 2]  [ 0 8 0 6 0]  [ 0 2 3 21 0]  [ 0 3 3 46 2]  [ 0 0 0 0 2]] |
| LOG-RQN | 10856 | 7 | XGB | 0.606 | 0.609 | 0.739 | 0.721 | 0.739 | 0.718 | 0.885 | [[20 0 1 2 0]  [ 2 3 4 3 1]  [ 0 2 14 11 0]  [ 1 1 2 50 0]  [ 0 0 0 1 1]] |
| LOG-RQN-Z | 11084 | 55 | XGB | 0.275 | 0.554 | 0.513 | 0.549 | 0.513 | 0.511 | 0.724 | [[15 2 0 5 1]  [ 0 3 3 7 0]  [ 0 2 6 21 0]  [ 0 1 9 36 7]  [ 0 0 0 0 1]] |
| LOG-NICG-Z | 12341 | 106 | XGB | 0.364 | 0.417 | 0.588 | 0.472 | 0.588 | 0.503 | 0.812 | [[12 3 0 6 0]  [ 1 8 0 5 0]  [ 0 8 0 22 0]  [ 0 3 0 50 0]  [ 0 1 0 0 0]] |
